# Chemical Barrier Proteins in Human Body Fluids

**DOI:** 10.3390/biomedicines10071472

**Published:** 2022-06-22

**Authors:** Gergő Kalló, Ajneesh Kumar, József Tőzsér, Éva Csősz

**Affiliations:** 1 Proteomics Core Facility, Department of Biochemistry and Molecular Biology, Faculty of Medicine, University of Debrecen, Egyetem tér 1, 4032 Debrecen, Hungary; kumar.ajneesh@med.unideb.hu (A.K.); tozser@med.unideb.hu (J.T.); cseva@med.unideb.hu (É.C.); 2 Biomarker Research Group, Department of Biochemistry and Molecular Biology, Faculty of Medicine, University of Debrecen, Egyetem tér 1, 4032 Debrecen, Hungary; 3 Doctoral School of Molecular Cell and Immune Biology, University of Debrecen, Egyetem tér 1, 4032 Debrecen, Hungary; 4 Laboratory of Retroviral Biochemistry, Department of Biochemistry and Molecular Biology, Faculty of Medicine, University of Debrecen, Egyetem tér 1, 4032 Debrecen, Hungary

**Keywords:** body fluid, AMP, interaction network, chemical barrier

## Abstract

Chemical barriers are composed of those sites of the human body where potential pathogens can contact the host cells. A chemical barrier is made up by different proteins that are part of the antimicrobial and immunomodulatory protein/peptide (AMP) family. Proteins of the AMP family exert antibacterial, antiviral, and/or antifungal activity and can modulate the immune system. Besides these proteins, a wide range of proteases and protease inhibitors can also be found in the chemical barriers maintaining a proteolytic balance in the host and/or the pathogens. In this review, we aimed to identify the chemical barrier components in nine human body fluids. The interaction networks of the chemical barrier proteins in each examined body fluid were generated as well.

## 1. Introduction

At those sites where the human body can make contact with potential pathogens, well-defined chemical barriers exist as part of the immune system. These chemical barriers provide passive protection against infections by diluting the colony number of pathogens and they can also actively inhibit bacterial growth due to the secretion of antimicrobial and immunomodulatory proteins/peptides (AMPs) [1]. The human body contains several contact sites: the eye, the oral cavity, the nose, the skin, the intestinal surface, and the urogenital tract. Each of these sites is protected by a chemical barrier maintained by different body fluids such as tears, sweat, saliva, nasal secretion, urine, intestinal mucus, and cervicovaginal fluid. These chemical barriers are made up of the secretion of various glands and epithelial cells and the characteristic composition of the chemical barrier makes the secreted AMP cocktail specific for a given body fluid [2]. The secreted AMP cocktail can adapt to various conditions [3,4]; therefore, the composition of the chemical barrier is continuously changing. Regarding the protein composition of the body fluids providing chemical barriers, it has been observed that the highly abundant proteins characteristic of each body fluid are part of the immune system and have protective roles. In the case of tears and sweat, it has been demonstrated that more than 90% of the secreted proteins have a role in the host defense mechanisms [5,6]. In this review, we aimed to examine the proteins identified in nine body fluids to identify the chemical barrier components. We also generated the interaction networks of these chemical barrier proteins in the examined body fluids.

## 2. Members of the Chemical Barriers

While some proteins/peptides such as defensins and LL-37 cathelicidin were first isolated due to their antimicrobial properties (so-called prototypic AMPs), other highly abundant proteins were initially recognized for their other functions, such as serum albumin [7], hemoglobin [8] or thrombin [9], and later, they or their peptides were found to have antimicrobial activity. While AMPs are parts of the innate immune system, members of the adaptive immune system, such as secreted immunoglobulin A (IgA), can be found in the chemical barriers as well. IgA is capable of blocking pathogens from attaching to intestinal epithelial cells [10], while the different AMPs are responsible for pathogen killing, by limiting their growth and modulating the immune reactions [2].

### 2.1. Prototypic AMPs in the Chemical Barriers

In the human body, the chemical barriers contain several prototypic AMPs, such as defensins, dermcidin and LL-37 cathelicidin [2]. Prototypic AMPs adsorb onto the bacterial cell membrane by electrostatic attraction or hydrophobic interactions [11] and insert into the membrane leading to the formation of channels and transmembrane pores or creating extensive membrane ruptures [2].

Defensins are small cationic peptides produced by epithelial cells forming a very stable 3D structure called defensin fold because of their cysteine-rich sequence [12]. The two subfamilies of human defensins (α- and β-defensins) show broad antimicrobial, antifungal, and antiviral activities [2,13]. Human α-defensins 1–4 are expressed by neutrophils and have been shown to protect against mycobacterium and various viruses including herpes simplex virus 1 and 2 (HSV-1, HSV2), cytomegalovirus (CMV), and also influenza virus; however, their activity may depend on the lipid composition of the viral envelope [12,14]. The α-defensins 1–3 also possess chemotactic activity for monocytes [12], α-defensins 5–6 are human enteric defensins constitutively expressed by Paneth cells and α-defensin 5 has been detected preferentially in the cervical mucosa and the oviduct [15]. Human β-defensins (hBDs) are coded on 11 genes but not all transcripts have been identified so far [16]. They can be considered as potential AMPs in epithelial cells providing a protective barrier against Gram-negative bacteria and *Candida* species [2]. Another important function of hBDs is their chemotactic activity toward various immune cells [17]. While some defensins, such as hBD1, show constitutive expression pattern, other members of this family have been found to be induced upon pathogenic or inflammatory stimuli [18]. In addition to their antimicrobial and immunomodulatory effects, some defensins have been identified as cancer-associated molecules with anti-tumor effects [19].

LL-37 cathelicidin is an α-helix type AMP of the body fluids mainly produced by epithelial cells and immune cells [2,20]. Similar to defensins, it exerts various antimicrobial activities against different types of pathogens, such as *Listeria monocytogenes*, *Staphylococcus aureus*, *Pseudomonas aeruginosa* or *Candida albicans* [21]. In addition, LL-37 cathelicidin has an important role in re-epithelialization during wound healing [22]. Besides the antimicrobial activity and wound healing, LL-37 cathelicidin has a neutralizing effect on lipopolysaccharide (LPS), it has chemotactic activity for different immune cells, modulates the transcriptional response of macrophages, stimulates vascularization, and exerts antitumor activity [21]. It should be noted that besides the LL-37 form, there are several cathelicidin-derived peptides with higher abundance in the different body fluids. These peptides are generated by proteolytic cleavage of the propeptide by proteases belonging to the kallikrein family [20]. These cathelicidin peptides exert various antimicrobial activity but they have no chemotactic activity [23].

### 2.2. Highly Abundant Body Fluid Proteins Are Constituents of the Chemical Barriers

Besides the prototypic AMPs, there are several proteins with much higher concentration as compared to those of prototypic AMPs. These proteins, e.g., lactotransferrin, lipocalins, lysozyme-C, extracellular glycoprotein lacritin, and prolactin-inducible protein are the highly abundant body fluid proteins with various defense functions.

It has been shown that lactotransferrin found in all body fluids is an active AMP against bacteria and parasites, and has been implicated in protection against cancer [2]. Because of its iron-sequestering activity, lactotransferrin has an important role in the prevention of bacterial colonization. Due to proteolytic cleavage, different lactotransferrin-derived peptides are produced such as Lf(1-11), lactoferricin, and lactoferrampin having stronger antimicrobial activity than the intact protein [24]. Besides its antimicrobial activity, lactotransferrin can promote autophagy [25] and it has also been shown that via its DNA-binding activity, the protein can alter the transcriptional machinery of different cells [26].

Lysozyme-C is a ubiquitous hydrolytic enzyme that exerts muraminidase activity, required for the peptidoglycan degradation of the bacterial cell wall [2]. Shimada et al., have shown that along with the degradation of the bacterial cell wall, the muraminidase activity is also important in the cleavage of pro-interleukin-1β (pro-IL-1β) to active IL-1β [27]. Besides its antibacterial activity, lysozyme-C has various functions including antifungal activity [28] and protection against HIV infection [29]. Lysozyme-C isolated from hen egg white also shows an immunomodulatory effect by suppressing the inflammatory response induced by LPS via inhibition of the phosphorylation of c-jun N-terminal kinase (JNK) in a murine model [30].

Lipocalins are a family of lipid and other small molecule binding proteins with protease inhibitor activity and with iron sequestrating capability that can limit the growth of pathogenic bacteria [31,32]. Lipocalins typically transport and/or store different small molecules, including vitamins, steroid hormones, and various secondary metabolites [33]. Studies have shown that lipocalins have roles in cancer development [33,34,35] and a study published by Mesquita et al. showed that the expression of lipocalin-2 is stimulated by amyloid-beta (Aβ) 1-42 in Alzheimer’s disease (AD) [36].

Lacritin is a secreted extracellular glycoprotein found in tears and saliva. The protein has various functions, including the modulation of lacrimal gland secretion [37], epithelial cell proliferation [38], and corneal wound healing [39]. Additionally, the C-terminal fragment of extracellular glycoprotein lacritin has proved bactericidal activity [40]. Regarding its effect on tear secretion rate, lacritin is a potent therapeutical target in dry eye disease [41].

Prolactin-inducible protein is an aspartyl protease [42] identified in various body fluids as part of the host defense system. Besides the protease activity, prolactin-inducible protein can modulate immune reaction by binding to immunoglobulin G and Zn-α-2-glycoprotein [43,44] and its elevated expression has been associated with breast cancer progression [42,45]. Interestingly, Edechi et al. proved that prolactin-inducible protein modulates antitumor immune response as well, suggesting a bifunctional role of this protein [46].

Dermcidin is the main skin AMP, which is also present in tears and exerts broad-spectrum antimicrobial activity [2,47]. Dermcidin is constitutively secreted by eccrine sweat glands and epithelial cells, and its secretion cannot be further induced either by skin injury or inflammation [48]. By post-secretory proteolytic processing, dermcidin is cleaved to several truncated dermcidin-derived peptides which differ in length and net charge. Reduced levels of dermcidin-derived peptides has been detected in the sweat of patients with atopic dermatitis in association with an impaired cutaneous antimicrobial defense [49], while the increased expression of dermcidin has been demonstrated in lung, prostate and pancreatic cancer cells [50,51].

### 2.3. AMPs with Lower Abundance in the Chemical Barriers

Besides the highly abundant AMPs, the different body fluids contain various proteins with antimicrobial activity in a lower concentration, such as members of the S100 family, RNase7, bactericidal/permeability increasing protein, azurocidin, cathepsin G, histatins, and hemocidins.

The members of the S100 family are Ca^2+^-binding proteins with EF-hand domains [52]. The family consists of 24 members with various intracellular and extracellular functions [53]. In the intracellular space, they can regulate cell proliferation, differentiation and apoptosis, cytoskeletal organization and Ca^2+^ homeostasis [53,54]. The secreted forms of S100 proteins have a paracrine effect on the nearby cells by receptor activation and regulation of different cell types such as immune cells, endothelial cells, and muscle cells [53,54]. S100A7 or psoriasin can be found on the surface of the skin secreted by the sweat and is a potent AMP against different pathogenic bacteria [2].

RNase7 mainly produced by keratinocytes and also released to the skin surface exerts a broad range of antimicrobial activity [55]. RNase7 is an inducible AMP, its expression can be enhanced by different stimuli such as inflammatory cytokines, growth factors, or pathogenic bacteria [56]. Besides the skin surface, RNase7 was also identified in the urinary tract acting as a potent AMP in the protection against different pathogens [57].

There are several AMPs, such as bactericidal/permeability increasing protein (BPI), azurocidin, myeloperoxidase or cathepsin G that are stored in the granules of neutrophil granulocytes and released upon the activation of the cell triggered by different pathogenic stimuli. BPI can be released to various sites of the human body and can initiate the permeabilization of the bacterial cell wall; therefore, it promotes the lysis of the bacteria [2,58]. Azurocidin acts against Gram-positive and negative bacterial strains and fungi as well [2] and it has been shown to have binding affinity to heparin [59]. Cathepsin G is a serine protease involved in the innate immunity, regulation of inflammatory pathways, degradation of extracellular matrix components and also in antigen presentation [60].

Histatins represent a group of 12 different histidine-rich peptides with antimicrobial activity in saliva [2]. Along with the defensins and LL-37 cathelicidin, histatins exert a broad range of antibacterial and antifungal activity but on the other hand, histatins can also act as protease inhibitors and they are involved in wound healing [61]. Moreover, these proteins can bind metal ions such as Cu^2+^ and Zn^2+^ that can modulate the activity of these proteins [61].

Hemocidins are a recently discovered group of AMPs that are derived from heme-binding proteins, such as hemoglobin and myoglobin [62]. Hemoglobin is the major protein of the red blood cells and has a crucial role in the oxygen transport via the cardiovascular system. Hemocidins are released from the hemoglobin α and β chains by limited proteolysis and exert antimicrobial activity against various pathogens: α (1–32), α (33–76), α (1–76), α (77–144), β (1–55), β (56–146), and β (116–146) hemocidins showing an antimicrobial effect against *Staphylococcus aureus*, *Escherichia coli, Streptococcus faecalis*, and *Candida albicans*, while β (56–72) hemocidin shows antimicrobial effect against *Escherichia coli* and *Streptococcus faecalis* [8]. It was recently demonstrated that hemoglobin-derived peptides can be generated by oxidative stress in atherosclerotic regions and intraventricular hemorrhage [63]. Myoglobin is an oxygen-binding protein located primarily in muscles serving as a local oxygen reservoir that can temporarily provide oxygen when blood oxygen delivery is insufficient during periods of intense muscular activity. Similar to hemoglobin, hemocidins can also be released from myoglobin: (1–55), (56–131), and (132–153) hemocidins exert antimicrobial activity against the above-mentioned pathogens [64].

### 2.4. Proteases and Protease Inhibitors

Proteases and their inhibitors are constitutive parts of the chemical barriers. A variety of cells involved in the defense against pathogenic microorganisms are expressing and secreting a wide range of proteolytic enzymes in order to degrade the bacterial/viral/fungal proteins involved in the life cycle of the microorganism. The proteolytic activity of different aminopeptidases, carboxypeptidases, and endopeptidases such as serine proteases, aspartyl proteases, and metalloproteases [65] as part of the chemical barriers, can modulate the homeostatic functions and control the microorganisms entering the human body [66,67,68].

Since the proteolytic activity is a double-edge sword capable of the degradation of the host proteins as well, the presence of protease inhibitors is crucial for the host. The human body expresses many different protease inhibitors with different specificity. Several protease inhibitors such as alpha-2-macroglobulin [69] and Kunitz-type protease inhibitors [70] have a broad inhibitory effect, while specific inhibitors such as alpha-1-antitrypsin [71] and antithrombin-III [72] act only on well-defined proteases. While the protease inhibitors of the host provide the defense against their own proteases, they can also inhibit the proteases secreted by pathogenic microorganisms [73].

### 2.5. Role of AMPs in Nosocomial Infections

Nosocomial infection, also known as a hospital-acquired infection, is contracted from the environment or staff of a healthcare facility affecting nearly 9% of patients according to the WHO [74]. The most common nosocomial infections are caused by common bacteria such as *Pseudomonas aeruginosa*, *Staphylococcus aureus, Acinetobacter baumannii*, and *Enterococci* species [75,76,77] usually leading to milder diseases. Bacterial infections are treated with antibiotics that kill or suppress the bacterial species sensitive to the antibiotic, while the resistant strains survive and may spread in the hospital [78,79]. Based on the literature data, the major mechanisms of antibiotic resistance include the hydrolysis of antibiotics, avoiding antibiotics targeting, prevention of antibiotics permeation, and the active efflux of antibiotics from bacteria [74]. The development of new antibiotics is far behind the increasing emergence of drug-resistant bacteria; therefore, alternative treatment for the infection of multidrug-resistant bacteria is an urgent task of biomedical research.

AMPs are promising candidates due to their fast killing kinetics, pharmacodynamic properties, and mechanisms of killing that overcome the common resistance mechanisms of pathogens [75]. The biofilm-disrupting properties of AMPs may also confer efficacy against multidrug-resistant bacterial infections associated with wounds and/or medical implants [75]. While preclinical studies were promising for numerous AMPs, most of the investigated AMPs failed in clinical studies [80,81,82]. Combination therapies of AMPs with antibiotics have also been investigated. The synergistic effect of proline-rich peptides with polymixin E was revealed in in vitro and in *Klebsiella pneumonia* infected mice models [83]. Besides the combination therapies, many drug delivery systems are investigated to deliver AMPs, such as nanocarriers and star polymers [84].

Wounds and other lesions are highly exposed to nosocomial infections due to the possible contamination with bacteria from the surface of the body or the environment. The wound healing process involves the activation of a variety of protease enzymes that can be altered by invading pathogens [85]. The analysis of wound fluids from the normal healing process and infected wounds revealed differences in the secreted proteins and processed peptides between the studied groups [85,86] that can lead to deteriorated healing. Since the proteolytic events are also crucial for the processing of AMPs [9,87], nosocomial wound infections may alter the host defense system by altering the production of AMPs in the wound fluid.

## 3. Chemical Barrier Proteins in Human Body Fluids

### 3.1. The Composition of the Chemical Barrier in Serum

Serum is the most widely used body fluid in biomedical sciences and contains various proteins, lipids, and small molecules. Serum is often used for the diagnosis of different pathological conditions and also for monitoring applied therapies. The serum proteins mainly originate from the liver and other tissues but the secretory activity of blood cells can also increase the number of serum proteins [88]. The protein concentration of healthy human serum varies between 60–80 mg/mL [89]. To date, more than 12,000 proteins have been found in human serum [90] with several highly abundant proteins such as albumin, immunoglobulins, transferrin, haptoglobin, and apoproteins that constitute approximately 90% of the total protein content [91].

Albumin is one of the most important transport proteins in the human body that can bind a variety of ligands such as ions, fatty acids, vitamins, hormones, and has a role in the maintenance of blood viscosity, in the regulation of cholesterol transport and coagulation events [92]. As a negative acute-phase protein, albumin has a role in the inflammatory response of the body [93] and Gum et al., have shown that albumin has antioxidant activity due to its N-terminal DAHK tetrapeptide [94]. Moreover, albumin exerts antimicrobial effect against *Candida albicans*, *Cryptococcus neoformans*, *Escherichia coli*, and *Staphylococcus aureus* [7].

Transferrin is the major iron transport protein having a role in cell growth and differentiation [95]. As an iron-binding molecule, transferrin can act against bacterial infections by sequestering the free iron from the serum, therefore, inhibits bacterial growth [96]. Furthermore, Ardehali. et al. proved that apotransferrin can reduce the surface adhesion of both Gram-positive and Gram-negative bacteria [97].

Haptoglobin can bind to hemoglobin thus preventing the iron loss from the free hemoglobin. The hemoglobin–haptoglobin complex can be removed via the reticuloendothelial system and by receptor-mediated endocytosis [98,99]. Haptoglobin has been shown to have immunoregulatory function via the regulation of T-cells and the expression of IL-6 and IL-10 cytokines [100]. The binding of hemoglobin can be considered as an iron-sequestering effect; thus, haptoglobin has a role in the defense against reactive oxygen species (ROS), that can be formed due to the free iron [100].

The major function of apoproteins is the construction of lipoproteins, such as chylomicron, very low density lipoprotein (VLDL), low-density lipoprotein (LDL), or high-density lipoprotein (HDL) that carry triglycerides, cholesterol, cholesterol esters, and other type of lipids in the circulation system [101]. Apoproteins also have a role in the host defense mechanisms; antimicrobial activity of Apo A1 against *Staphylococcus epidermidis* has been described [102] and antimicrobial peptides derived from Apo B acting against *Salmonella* strains have also identified [103].

**Figure 1:**
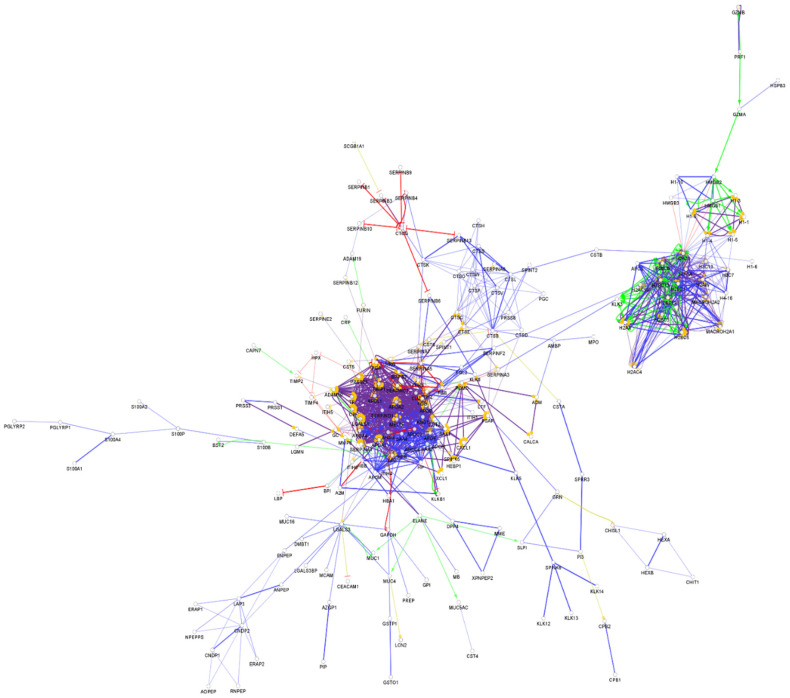
Partial network view of the interaction network of the chemical barrier proteins in serum. Each circle represents a protein and the lines indicate interactions. The lines with an arrow represent activation, blocking lines represent inhibition, and simple lines represent protein–protein interaction. Line color indicates the type of interaction: green color refers to activation, red color to inhibition, blue color to binding, yellow color to co-expression, and purple color to catalysis. The proteins are labeled with their gene name. The high resolution and complete network image of this network is presented in Figure S1.

From the approximately 12,000 identified serum proteins, 422 can act in the first line of host defense (Table S1). The identified chemical barrier proteins were subjected to network analysis, and the interaction network of these proteins was generated by CluePedia v.1.5.7 as we described earlier by our group [104]. The interaction network of the chemical barrier proteins in serum is visualized in Figure 1.

The network analysis revealed that most of the chemical barrier proteins are part of two core clusters that interact with each other. The identified histone proteins form one of the clusters, while more than 80% of the other identified chemical barrier proteins form the other highly interconnected cluster. In this cluster, we can find many different hub proteins such as alpha-1-antitrypsin, metalloproteinase inhibitor 1, apolipoprotein AI, apolipoprotein E, serotransferrin, and cystatin-C with a wide range of interactions. On the other hand, we identified seven additional clusters with a small number of proteins without interactions with the core hubs (Figure S1). The network architecture is serum showed the highest complexity among the examined body fluids.

### 3.2. Tears, the Chemical Barrier of the Eye

Tear is a protein-, metabolite-, and salt-rich fluid produced by the lacrimal glands, Meibomian glands, and conjunctival goblet cells. The normal tear production rate is approximately 2 μL/min [105] and its typical protein concentration is 5–7 μg/μL [106]. The functions of the tear film are the lubrication of the eye, delivery of nutrients, and maintaining the refractivity of the cornea [107]. Besides these roles, tears create an effective chemical barrier on the surface of the eye via secreted AMPs, which provide protection against pathogens [108].

Currently, more than 1800 proteins have been identified in tears by state-of-the-art proteomics techniques [109]. The major tear proteins, such as lysozyme-C, prolactin-inducible protein, lactotransferrin, and lacritin, have antimicrobial activity; therefore, they are involved in the defense against pathogens [5]. While many of the tear proteins are produced by the lacrimal glands, some of them originate from epithelial cells, such as dermcidin and defensins, and there are also proteins, such as albumin, originating from the blood [110].

**Figure 2:**
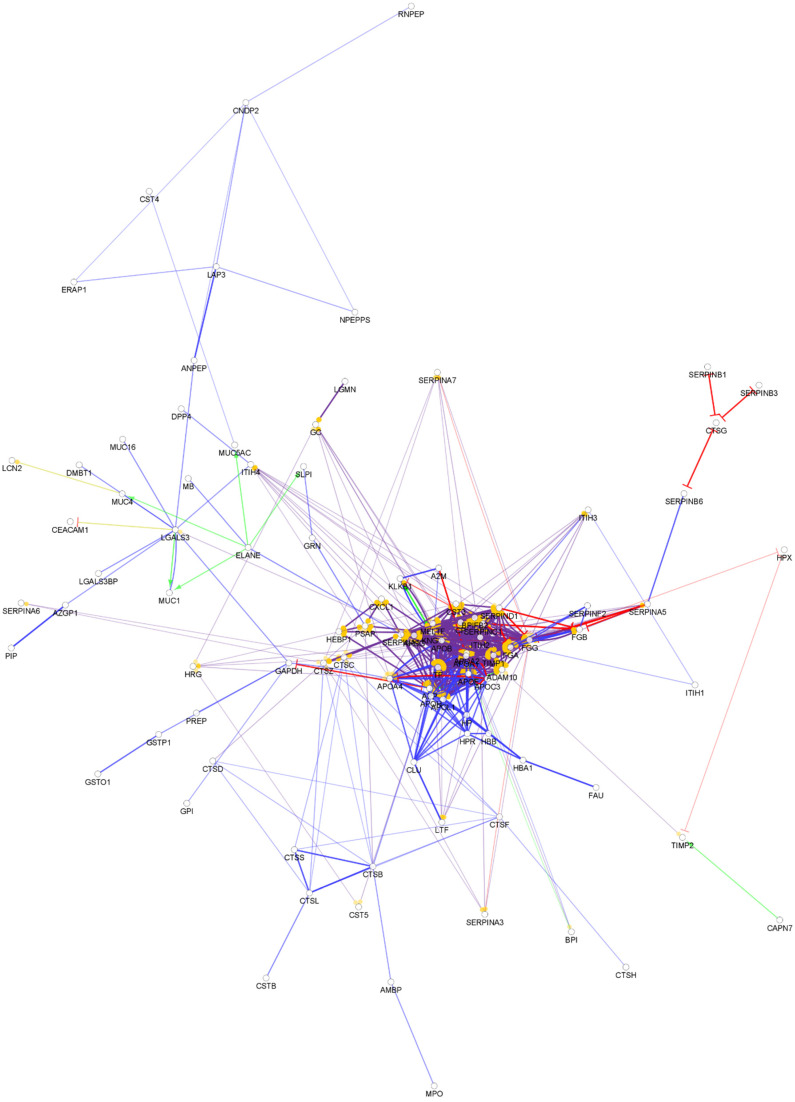
Partial network view of the interaction network of the chemical barrier proteins in tears. Each circle represents a protein and the lines indicate interactions. The lines with an arrow represent activation, blocking lines represent inhibition, and simple lines represent protein–protein interaction. Line color indicates the type of interaction: green color refers to activation, red color to inhibition, blue color to binding, yellow color to co-expression, and purple color to catalysis. The proteins are labeled with their gene name. The high resolution and complete network image of this network is presented in Figure S2.

The examination of the tear proteome revealed 200 proteins secreted to the surface of the eye and building the chemical barrier against pathogens (Table S2). Overall, 97% of the AMPs found in tears are also components of the chemical barrier of the serum. Regarding the protein–protein interaction network of the chemical barrier proteins in tears (Figure 2), we can say that there are similarities at the level of small, unrelated clusters, but in case of the tears, the majority of AMPs are organized only into one core cluster. Most of the histone-derived AMPs found in serum are missing from tears.

Compared to serum, the number of hub proteins such as protease inhibitors, transferrin, kininogen-1, amyloid-beta precursor protein, and alpha-2-HS-glycoprotein were similar. We also identified three additional small clusters with no connections to the core cluster (Figure S2). Our results demonstrate that in spite of the similarities, the networks characteristic to serum and tear AMPs are different from each other.

### 3.3. Salivary Proteins in the Defense of the Oral Cavity

Saliva is a complex mixture of organic and inorganic compounds secreted from major and minor salivary glands and the gingival crevice [111]. Saliva is a very dilute body fluid composed of approximately 99% water with 0.7–2.4 µg/µL protein concentration [112,113]. The protein concentration shows high variability between the individuals depending on the age, sex, sample collection time, and the health status of the oral cavity. Saliva contains more than 2700 proteins [109] and the most abundant ones are α-amylase, mucins, cystatins, proline-rich peptides, and serum albumin [114]. Similar to tears, the abundant salivary proteins are part of the immune system due to their antimicrobial activity, antioxidant function, and protective role against microbial proteases [115].

Amylases are mostly known by their hydrolytic activity on polysaccharides, and due to their hydrolytic activity, amylases can inhibit biofilm formation by cleaving the polysaccharide backbone of extracellular polymeric substances [116]. At the same time, there is evidence showing that amylase can bind to the amylase-binding protein of *Streptococcus* species and induce biofilm formation [116]. Therefore, the function of amylase in the biofilm formation is still unclear.

Mucins are high-molecular-weight glycoproteins composed of a core protein coupled to carbohydrate chains and sulfate groups. They have characteristic amino acid repeats and a Cys-rich domain that has a key role in multimerization and mucus function by forming disulfide bridges [117]. Acting in the host defense, salivary MUC5B and MUC7 can interact with numerous bacteria such as the *Streptococcus* species or *Pseudomonas aeruginosa* and pathogenic fungi such as *Candida albicans* [118].

Cystatins represent a family of structurally conserved, low-molecular-weight cysteine protease inhibitors found in nearly everywhere in the human body and in body fluids. Secreted cystatins regulate extracellular proteases such as a papain-like protease and matrix metalloproteases, while intracellularly they can regulate the activity of cathepsin C [119]. Along with the regulation of human proteases, cystatins can also inhibit the proteases secreted by pathogenic microorganisms, being in this way important members of the chemical barrier.

Proline-rich proteins (PRPs) and their peptide fragments represent a major fraction of salivary proteome produced by the parotid and submandibular glands [120]. PRPs can be divided into two subgroups: acidic PRPs that can adhere to the surface of the teeth and basic PRPs that cannot adhere to teeth but can bind polyphenols and bacteria [121]. By binding specific bacterial strains such as *Streptococci*, PRPs play an important role in preventing caries formation [121].

**Figure 3:**
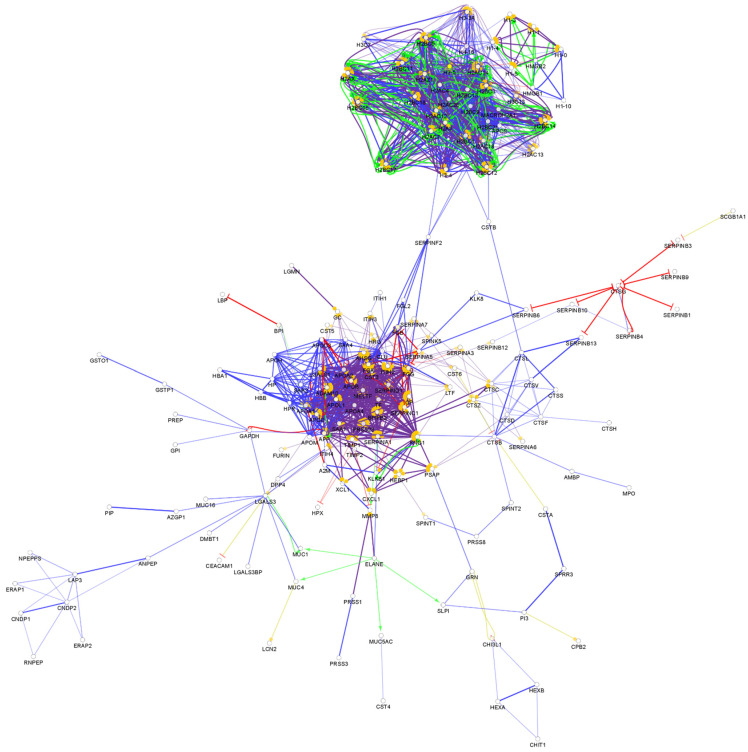
Partial network view of the interaction network of the chemical barrier proteins in saliva. Each circle represents a protein and the lines indicate interactions. The lines with an arrow represent activation, blocking lines represent inhibition, and simple lines represent protein–protein interaction. Line color indicates the type of interaction: green color refers to activation, red color to inhibition, blue color to binding, yellow color to co-expression, and purple color to catalysis. The proteins are labeled with their gene name. The high resolution and complete network image of this network is presented in Figure S3.

Among the more than 2700 so far identified salivary proteins, 319 proteins take part in the composition of the chemical barrier of the oral cavity (Table S3). The interaction network of the salivary chemical barrier proteins was generated (Figure 3).

Based on the network analysis, we can observe that the chemical barrier proteins in saliva are organized in two core clusters interacting with each other. Similar to the interaction network of serum AMPs, the histones are organized in one core cluster and the majority of the other chemical barrier proteins form the other core cluster with multiple hub proteins such as alpha-1-antitrypsin, metalloproteinase inhibitor 1, fibrinogen alpha and gamma chains, apolipoprotein AI, apolipoprotein E, and serotransferrin. Those proteins which are not part of these two core clusters are organized in five additional small clusters (Figure S3). The data indicate that the chemical barrier of saliva shows high similarity to the chemical barrier of serum and some similarity to the AMPs characteristic for tears.

### 3.4. Sweat–The Chemical Barrier of the Skin

The human skin creates an effective barrier against pathogens as a first line of host defense of the body. Besides the provided physical barrier by the cornified envelope, the skin also creates a chemical barrier via AMPs secreted by epithelial cells, sebocytes, and keratinocytes [122]. Sweat is composed of more than 99% water, making it a very dilute body fluid [123]. To date, more than 1200 sweat proteins have been identified by proteomic analyses [109]. Along with the saliva and tear fluid, the abundant sweat proteins are part of the innate immune system. Our workgroup demonstrated that 91% of the secreted sweat proteome is made up of six highly abundant proteins which are dermcidin, clusterin, ApoD, prolactin-inducible protein, and serum albumin [6]. Sweat protein content provides an effective defense against pathogens, and is involved in tissue regeneration after injury [2]. Some AMPs have been shown to be expressed constitutively (e.g., dermcidin) while others have been found to be inducible upon pathogenic stimuli (e.g., LL-37 cathelicidin, hBD2, and hBD3) [2,122,124]. Besides these prototypic AMPs, the presence of lysozyme-C and lactotransferrin in the sweat has been reported as well [2,125].

The secreted form of clusterin belongs to the family of extracellular chaperones and can be found in almost all body fluids. The major functions of these proteins are the maintenance of fluid–epithelial interface homeostasis, and the prevention of the onset of inflammation [126,127]. The study of Jeong et al. suggests that clusterin can interact with matrix metalloproteinases inhibiting their enzymatic activity and it is also suggested that the protein is able to reduce keratinocyte damage and inflammation of the skin [126].

**Figure 4:**
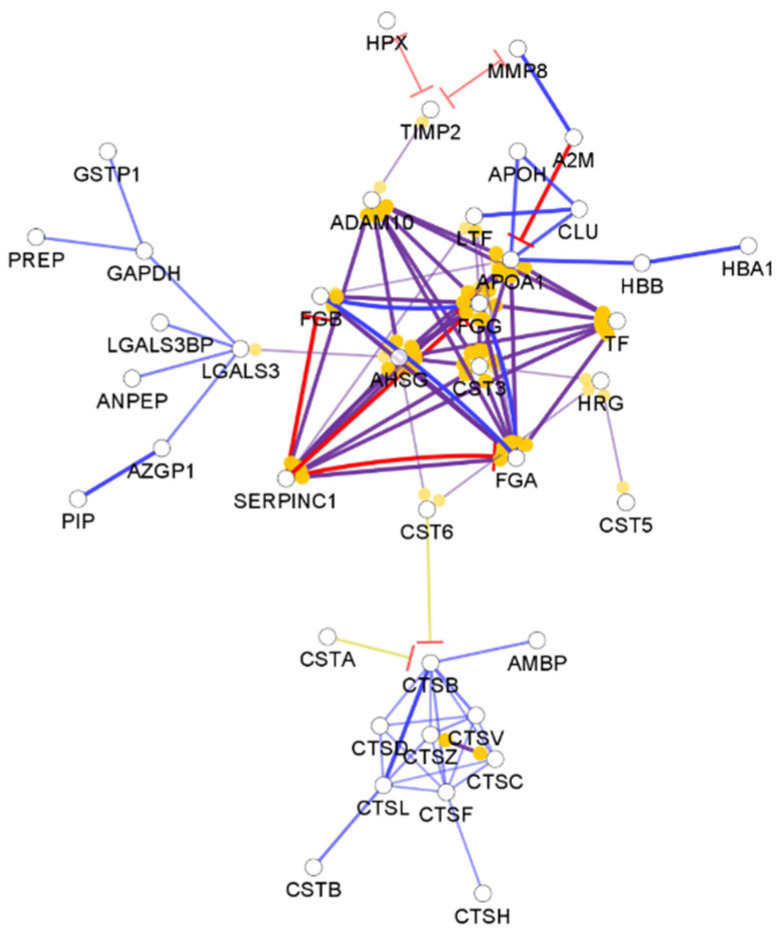
Partial network view of the interaction network of the chemical barrier proteins in sweat. Each circle represents a protein and the lines indicate interactions. The lines with an arrow represent activation, blocking lines represent inhibition, and simple lines represent protein–protein interaction. Line color indicates the type of interaction: green color refers to activation, red color to inhibition, blue color to binding, yellow color to co-expression, and purple color to catalysis. The proteins are labeled with their gene name. The high resolution and complete network image of this network is presented in Figure S4.

By checking the function of the identified sweat proteins, 128 sweat AMPs were identified (Table S4). The interaction network of the sweat chemical barrier proteins shows a distinct feature compared to the networks observed in the case of other body fluids (Figure 4).

Nearly half of the identified chemical barrier proteins did not interact with each other (Figure 4). Unlike in the other networks, few hub proteins such as antithrombin-III, disintegrin, and metalloproteinase domain-containing protein 10, fibrinogen alpha and gamma chains, serotransferrin, and lactotransferrin are holding most of the interactions. Besides the core cluster, the interacting AMPs formed three additional small clusters (Figure S4). The data indicate that the sweat chemical barrier is formed in a completely different way than the other examined body fluids highlighting the uniqueness of sweat.

### 3.5. The Chemical Barrier of the Nasal Secretion

The nasal secretion provides protection in the upper respiratory tract by creating an effective chemical barrier in the nasal cavity. The production rate and the protein concentration of nasal secretion show high variability. The protein concentration varies between 0.8 mg/mL and 32.7 mg/mL [128,129]. Nasal secretion contains AMPs such as lactotransferrin, lysozyme-C [130], and immunoglobulin molecules [131]. As a result of various studies, more than 2000 proteins have been identified in the nasal mucus and many of them are related to the host defense system [132,133,134,135].

**Figure 5:**
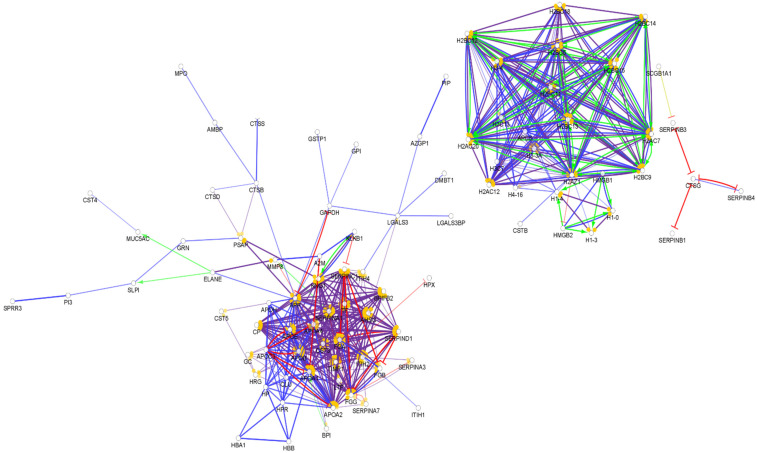
Partial network view of the interaction network of the chemical barrier proteins in the nasal secretion. Each circle represents a protein and the lines indicate interactions. The lines with an arrow represent activation, blocking lines represent inhibition, and simple lines represent protein–protein interaction. Line color indicates the type of interaction: green color refers to activation, red color to inhibition, blue color to binding, yellow color to co-expression, and purple color to catalysis. The proteins are labeled with their gene name. The high resolution and complete network image of this network is presented in Figure S5.

The analysis of the available data showed that 164 AMPs are present in the nasal secretion (Table S5). The protein–protein interactions among the AMPs are mainly organized into two core clusters (Figure 5).

Our analysis of the interactions among the nasal AMPs highlighted a similar pattern to the interactions found in saliva. The majority of the chemical barrier proteins in the nasal secretion were organized into two core clusters, one core cluster composed of the histone proteins and the other containing the majority of the other chemical barrier proteins. The hub proteins (alpha-1-antitrypsin, fibrinogen alpha and gamma chain, apolipoprotein AI, apolipoprotein E, and serotransferrin) were similar in the above-mentioned body fluids. We also observed that there is no interaction between the core clusters in the nasal secretion. We also identified four small clusters (Figure S5). The nasal and oral cavity are connected to each other and in some cases similar factors may influence the composition of the secreted AMP cocktail, the interaction pattern of the AMPs observed in these two body fluids show similarities but at the same time clearly indicate the differences as well.

### 3.6. AMPs Secreted into the Urine

Urine is formed by the kidneys as a result of ultrafiltration of the plasma to eliminate waste products such as urea, metabolites, or xenobiotics from the body. The protein concentration in urine under physiological conditions is very low, nearly 1000 times less compared to other body fluids such as plasma [136]. Urine contains more than 7000 proteins; unsurprisingly, many of these proteins are part of the defense system in the body [109]. The most abundant urine proteins have been found to be serum albumin, uromodulin, α-1-microglobulin, kininogen and various immunoglobulin chains [137].

Uromodulin (or Tamm-Horsfall protein) is produced by the renal epithelial cells and has various roles in the body, such as the regulation of salt transport, protection against kidney stones, and an antimicrobial effect against pathogens, as well as immunomodulatory functions [138]. It has been shown that uromodulin can form filaments that act as a multivalent decoy for pathogenic bacteria [139]. The resulting uromodulin pathogen aggregates prevent bacterial adhesion to epithelial glycoproteins and promote pathogen clearance as they are excreted with urine [140]. Uromodulin also shows immunomodulatory effects by binding to EGF-like receptors, cathepsin G and lactoferrin, to enhance the phagocytic activity of polymorphonuclear leukocytes, production of proinflammatory cytokines by monocytes/macrophages, and lymphocyte proliferation via the activation of mitogen-activated protein kinase signaling pathways [141].

A-1-microglobulin is a member of the lipocalin family responsible for the protection against free radicals and is involved in natural tissue repair [142]. The protein is expressed by the liver and after secretion into the blood, the protein can form complexes with other macromolecules or can exist in a free form. The free form can pass through the glomeruli filter to the urine [142]. As an antioxidant molecule, α-1-microglobulin has reductase activity [143], exerts free radical scavenging activity [144], and can bind to free hem [145].

Kininogens represent a group of multifunctional glycoproteins synthesized by the liver and predominantly circulate in the blood but they can also be found in other body fluids such as urine [146]; however, the function of the urinary kininogens are still unclear. In the blood, kininogen can interact with plasma kallikrein to produce bradykinin in the contact activation of the coagulation system [147]. It was demonstrated in the early 1980s that kininogen has a role in the regulation of blood pressure and in the modulation of water and salt transport in the kidney [148]. Besides the above-mentioned functions, kininogen is able to bind to the surface of a variety of pathogen microorganisms such as *Streptococcus pyogenes* [149] or *Candida albicans* [150] leading to activation of the contact system in the blood. In addition, Sonesson et al. demonstrated that due to the proteolytic process of kininogen, peptide fragments with antifungal activity are generated [151].

**Figure 6:**
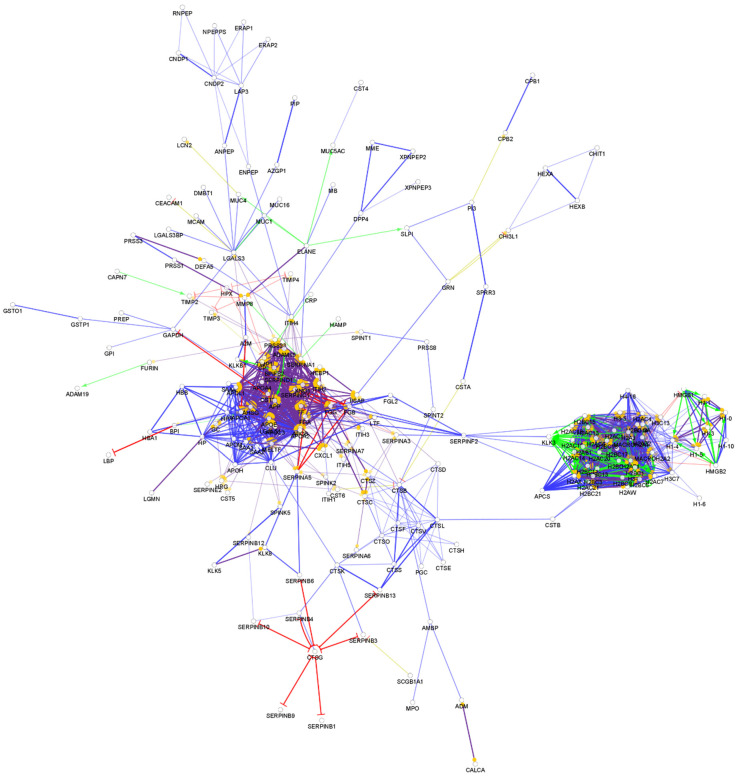
Partial network view of the interaction network of the chemical barrier proteins in urine. Each circle represents a protein and the lines indicate interactions. The lines with an arrow represent activation, blocking lines represent inhibition, and simple lines represent protein–protein interaction. Line color indicates the type of interaction: green color refers to activation, red color to inhibition, blue color to binding, yellow color to co-expression, and purple color to catalysis. The proteins are labeled with their gene name. The high resolution and complete network image of this network is presented in Figure S6.

From the so-far identified urine proteins, 396 have antimicrobial and/or immunomodulatory activity (Table S6). The interaction network of the identified AMPs in urine was generated and visualized, as can be seen in Figure 6.

The network analysis of the urine chemical barrier proteins indicates a similar pattern to the one identified in serum: one core cluster with the histone proteins and another one with the majority of other AMPs. Similar to saliva and serum, in the case of urine, there are connections between the two major clusters. In the case of saliva and urine, the SERPINF2 provides a hub linking the two clusters. Six small clusters were observed in case of urine as well (Figure S6). Since the urine is filtered from the serum and low-molecular-weight proteins can be transferred through the glomeruli, the similarity between the interaction network of serum and urine is not surprising. However, differences between the organization of the connections and hub proteins are also observed, indicating that the urine has a distinct chemical barrier.

### 3.7. Antimicrobial and Immunomodulatory Properties of Cervicovaginal Fluid

Cervicovaginal fluid is constitutively secreted from the vagina, cervix, and the upper genital tract [152] containing water, nutrients, electrolytes, proteins, and different cells [153]. The mucosa creates an effective physical barrier against invading microorganisms due to the hindrance of the adherence of bacteria to the surface of epithelial cells [154]. The protein composition of the vaginal fluid can be influenced by various factors, such as the varying ratio of estrogens and progesterone that can cause changes in the amount of secreted proteins [154]; thus, the determination of the normal protein concentration is practically impossible. To date, more than 900 proteins have been identified in the cervicovaginal fluid [109]. The majority of the proteins are proteases including complement factors, coagulation components and members of the kallikrein family, and protease inhibitors such as SERPIN and Kazal type (SPINK) serine protease inhibitor proteins [155,156]. Besides the proteases and protease inhibitors, various AMPs are also found in the vaginal fluid such as lysozyme-C, lactotransferrin, LL-37 cathelicidin, S100A7, BPI, and α-defensins [156].

**Figure 7:**
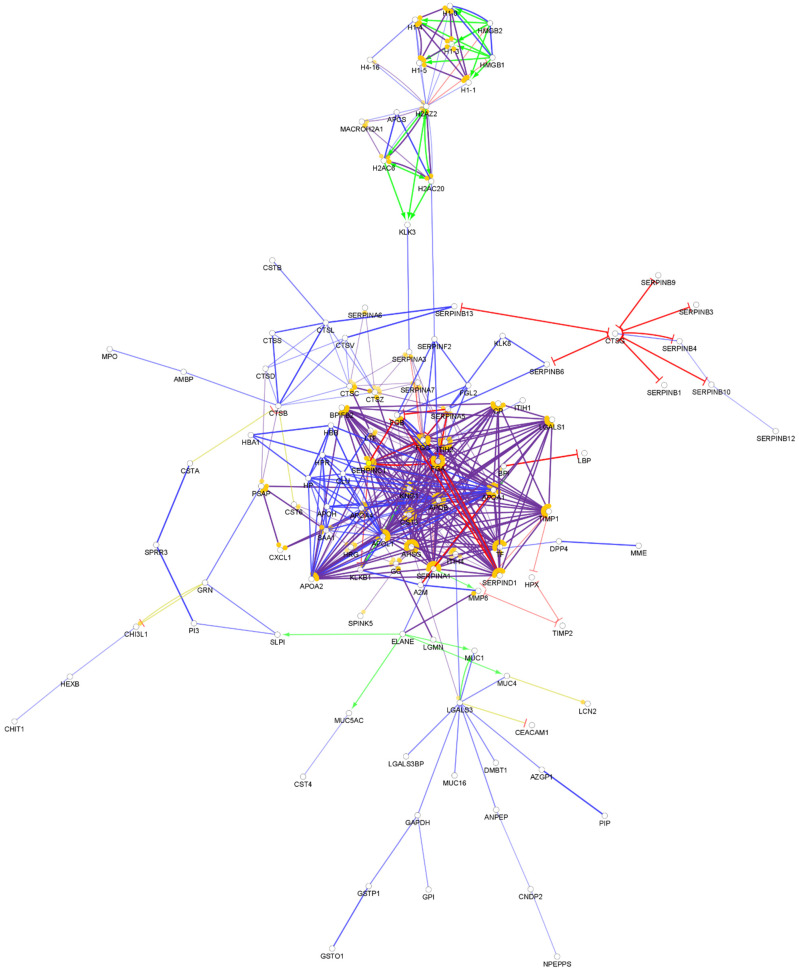
Partial network view of the interaction network of the chemical barrier proteins in cervicovaginal fluid. Each circle represents a protein and the lines indicate interactions. The lines with an arrow represent activation, blocking lines represent inhibition, and simple lines represent protein–protein interaction. Line color indicates the type of interaction: green color refers to activation, red color to inhibition, blue color to binding, yellow color to co-expression, and purple color to catalysis. The proteins are labeled with their gene name. The high resolution and complete network image of this network is presented in Figure S7.

By examining the identified vaginal fluid proteins, we identified 224 AMPs among them (Table S7). The interaction network of the identified AMPs in cervicovaginal fluid was generated (Figure 7).

**Figure 8:**
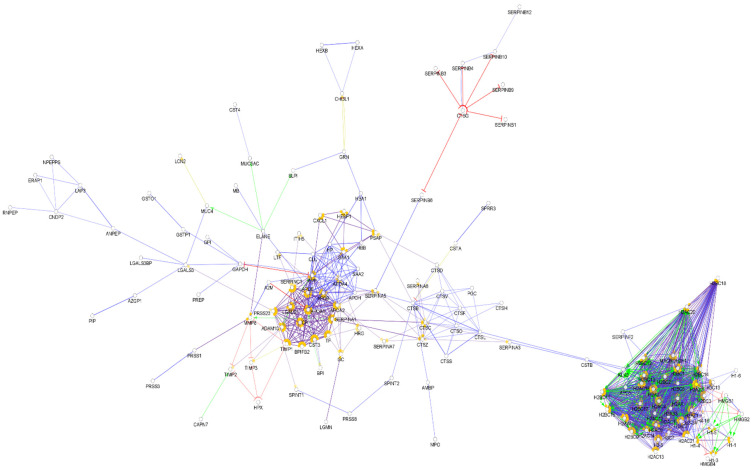
Partial network view of the interaction network of the chemical barrier proteins in the seminal fluid. Each circle represents a protein and the lines indicate interactions. The lines with an arrow represent activation, blocking lines represent inhibition, and simple lines represent protein–protein interaction. Line color indicates the type of interaction: green color refers to activation, red color to inhibition, blue color to binding, yellow color to co-expression, and purple color to catalysis. The proteins are labeled with their gene name. The high resolution and complete network image of this network is presented in Figure S8.

Since only a few histone proteins were identified in the cervicovaginal fluid, the pattern of this interaction network was different from those of the so far-mentioned body fluids (Figure 8). Most of the AMPs are clustered to one core cluster, while the histones form a smaller cluster. Several major hub proteins were identified such as alpha-1 antitrypsin, kininogen-1, serotransferrin, apolipoprotein AI apolipoprotein AIV, and fibrinogen alpha and gamma chain. In addition, five small clusters were identified (Figure S7). This network was only slightly similar to the network of AMPs observed in serum, saliva, nasal secretion, and urine.

### 3.8. Chemical Barrier Proteins in the Seminal Fluid

The seminal fluid contains spermatozoa and seminal plasma, and its primary function is the fertilization of the oocyte. It contains many different bioactive signaling factors, including cytokines, prostaglandins, sex hormones, glycans, nucleic acids, and other small molecules that elicit molecular and cellular changes in the female reproductive tract [157]. These bioactive molecules exist either in soluble form, encapsulated within seminal plasma extracellular vesicles, or associated with spermatozoa [157]. Besides the interaction with the female reproductive tract, seminal fluid has also been found to interact with the female immune system [158]. Currently, more than 4000 proteins have been identified in human seminal plasma [109] and the most abundant proteins are fibronectin, semenogelins, lactotransferrin, laminin, and serum albumin [159].

Fibronectin is an extracellular glycoprotein ubiquitously found in the extracellular matrix and in biological fluids with a major function to connect cells to the extracellular matrix [160]. Bacteria can also bind to fibronectin through their receptors; therefore, fibronectin may play an important role in the attachment of bacteria and the infection of the host cells [161]. Laminin is also a part of the extracellular matrix and is a major component of the basal membrane [162], participating in the adhesion of sperms to the oocyte [163].

Semenogelins (semenogelin I and semenogelin II) together with fibronectin, are maintaining the gel-like coagulum of newly ejaculated semen [164]. Semenogelins have been found to inhibit the motility of the sperms but due to their proteolytic processing by prostate-specific antigen, the movement of sperms can be initiated [165]. In addition, semenogelins and their processed forms participate in Zn^2+^ shuttling, hyaluronidase activation, and exert antimicrobial activity [165]. Peptides derived from semenogelin I can act against various pathogenic bacteria, such as *Escherichia coli* and *Pseudomonas aeruginosa* [166,167].

Among the more than 4000 seminal fluid proteins, 284 are AMPs (Table S8).

The interaction network of the proteins identified as chemical barrier components in the seminal fluid shows a similar pattern to the protein–protein interaction network observed in the urine (Figure 8).

We observed the two major clusters composed of the histone proteins and the majority of the remaining AMPs, but this later one did not contain that many interactions as in the case of urine. We also observed that the core clusters were interacting with each other. The hub proteins identified were very similar in the examined body fluids. Besides the proteins part of the core clusters, we identified proteins forming six small additional clusters (Figure S8).

### 3.9. AMPs in the Cerebrospinal Fluid

Cerebrospinal fluid (CSF) is secreted into the brain ventricles and the cranial and spinal subarachnoid spaces predominantly by the choroid plexus [168]. The secretion rate of CSF varies between 400 and 600 mL/day with a protein concentration of 15–45 mg/dL [169]. More than 4000 proteins have been identified in the human CSF so far [109]. Approximately 80% of the total proteins in CSF originate from the blood by size-dependent filtration across the blood–brain barrier, the other proteins originate from the drainage of interstitial fluid from the central nervous system [170]. The most abundant blood-derived proteins in CSF are serum albumin, immunoglobulins, transthyretin, transferrin, and α1-antitrypsin [171,172]. Several blood-derived proteins, such as prostaglandin D2 synthase, S-100B, tau protein, and cystatin C have higher concentration in the CSF than in serum [173,174].

Transthyretin is a transport protein in the plasma and in CSF transporting thyroxine and retinol to various parts of the body [175]. Studies from animal models and human samples also suggest the importance of transthyretin in the preservation and regulation of memory function and behavior, protection against neurodegeneration, neuroprotection in response to ischemic injury, and nerve regeneration [175]. Sharma et al. also suggested the role of this protein in the oxidative stress response [176]. It has been described that transthyretin inhibits the biofilm formation of *Escherichia coli* [177].

α1-antitrypsin is one of the most abundant serine protease inhibitors in the human body and is a well-known acute phase protein [178]. The protein is mainly synthesized in the liver, but it is also synthesized by monocytes, macrophages, pulmonary alveolar cells, and by intestinal and corneal epithelium [179]. As a potent protease inhibitor, α1-antitrypsin protects the host cells from the activity of proteases during inflammation, and by inhibiting proteases released from pathogenic bacteria, the protein has an important role in the inhibition of bacterial colonization [178,179].

Prostaglandin D2 synthase catalyzes the conversion of prostaglandin H_2_ (PGH_2_) generated by cyclooxygenases to PGD_2_. PGD_2_ stimulates G-protein coupled receptors leading to the regulation of the circadian rhythm, food intake, pain perception, myelination, and adipocyte differentiation [180]. PGD_2_ has been recognized as a proinflammatory molecule that can initiate IgE-mediated type I acute allergic response [181]. On the contrary, the anti-inflammatory effects of PGD_2_ have also been described [181]. PGD_2_ can also induce the expression of hBD3; thus, it can initiate the antimicrobial response against pathogenic microorganisms [182].

Tau protein is a microtubule-associated protein in the nervous system regulating the axonal transport and signaling pathways within and between neurons [183]. In addition, tau protein has an important role in the maintenance of the blood-brain barrier, since tauopathies can disrupt this barrier [184]. Interestingly, tau protein can serve as a scaffold for the binding of peptides that exert antimicrobial properties [185].

**Figure 9:**
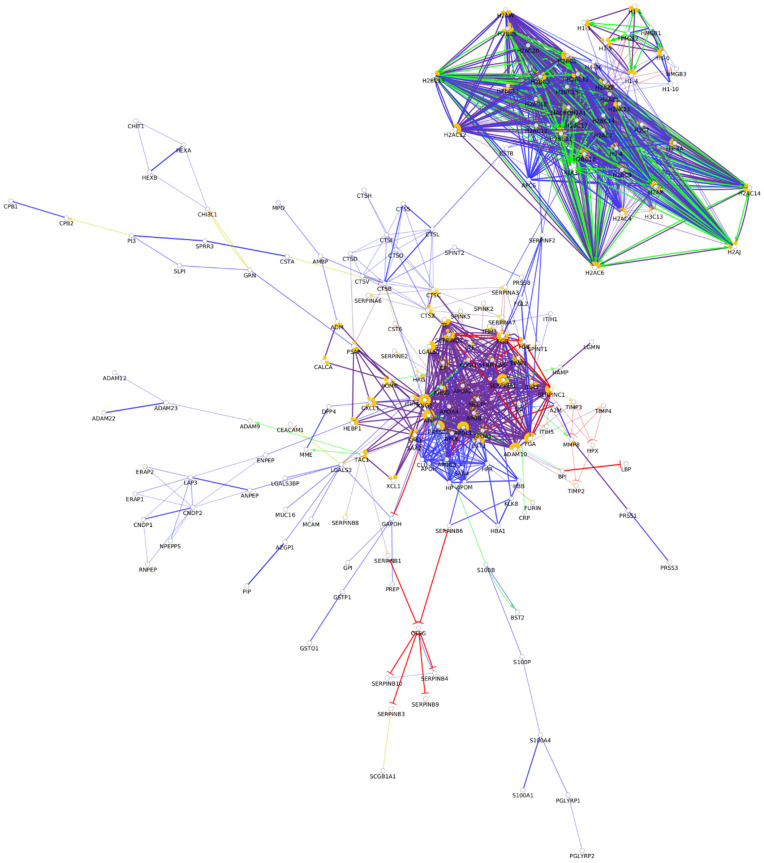
Partial network view of the interaction network of the chemical barrier proteins in CSF. Each circle represents a protein and the lines indicate interactions. The lines with an arrow represent activation, blocking lines represent inhibition, and simple lines represent protein–protein interaction. Line color indicates the type of interaction: green color refers to activation, red color to inhibition, blue color to binding, yellow color to co-expression, and purple color to catalysis. The proteins are labeled with their gene name. The high resolution and complete network image of this network is presented in Figure S9.

The examination of the identified CSF proteins revealed that 338 take part in the chemical barrier (Table S9) and their protein–protein interaction shows a similar architecture to the ones observed in serum, saliva, nasal secretion, urine, and seminal fluid (Figure 9).

The majority of the CSF chemical barrier proteins are organized into two core clusters, one cluster of the histones and related proteins and another cluster for the majority of the remaining chemical barrier proteins with several hubs, such as apolipoprotein AII, apolipoprotein L1, alpha-1 antitrypsin, Disintegrin and metalloproteinase domain-containing protein 10, serotransferrin and fibrinogen alpha and gamma chains. We also observed that the core clusters are interacting with each other. Compared to the serum, the pattern of the network is slightly different, and different connections and sub-clusters can be observed. Besides the core clusters, five additional small clusters were identified (Figure S9).

## 4. Comparison of the Protein–Protein Interaction Network in the Examined Body Fluids

The examination of the interaction networks revealed similar network architecture in the case of the different body fluids. Most of the proteins were organized into two core clusters: the cluster of histone proteins and the cluster of the other AMPs in networks except the one characteristic to sweat. These two clusters were connected to each other in all of the examined body fluids except the nasal secretion. The network generated from the sweat chemical barrier proteins was different from all the other networks highlighting the distinct feature of sweat.

**Figure 10:**
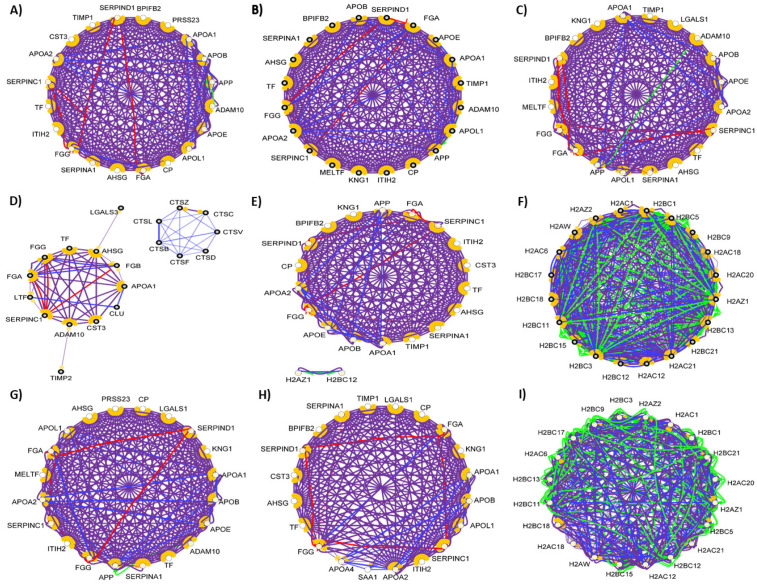
Interaction between the top 20 hub proteins in (**A**) serum, (**B**) tears, (**C**) saliva, (**D**) sweat, (**E**) nasal secretion, (**F**) urine, (**G**) CSF, (**H**) cervicovaginal fluid, and (**I**) seminal fluid. The blue lines represent binging, green lines represent activation, red lines represent inhibition, and purple lines represent catalysis. The proteins are labeled with their gene name.

In order to examine the hub proteins, we identified the top 20 hub proteins [104] in all of the examined networks (Figure 10).

Our analysis revealed that there is no major difference between the 20 hub proteins with the highest number of connections in serum, tears, saliva, nasal secretion, CSF, and cervicovaginal fluid. The chemical barrier of the sweat was different from the others regarding the top hub proteins, while in case of urine and seminal fluid, the histone proteins were found to be the proteins with most of the interactions. Considering the anatomical and physiological characteristics, the similarity between these two body fluids regarding chemical barrier is expected. Very likely, both body fluids have to cope with similar challenges regarding the antimicrobial protection, similarity reflected at the level of AMPs and their interaction networks.

Our results indicate that the composition and the interaction network of the examined human body fluids show high similarity. There is a considerable overlap between the protein composition of the chemical barrier, indicating the importance of the secreted AMPs and the proteases and protease inhibitors at the contact sites of the human body. We identified that approximately 9% of the AMPs can be found in all of the examined body fluids composing the core protein content of the chemical barriers and the other proteins make the body fluids specific for their localization and function.

## 5. Conclusions

In this review, we collected information about proteins and peptides taking part in the formation of chemical barriers of nine human body fluids and we present a comprehensive list of these components. We also generated the protein–protein interaction networks of the chemical barrier proteins and we highlighted that the interaction pattern is similar to most of the body fluids with slight differences. The network analysis proved that in case of saliva, nasal secretion, urine, and CSF, the interaction pattern is similar to the one observed in the case of serum. Regarding the networks of tears and cervicovaginal fluid, differences were observed compared to the other networks, while the network of the sweat proteins was completely different from the others. The networks of the urine and seminal fluid proteins, respectively, show high similarity in the organization of the connections and in the top hub proteins, indicating a strong relationship between these body fluids.

## Data Availability

Data sharing not applicable.

## Supplementary Materials

The following supporting information can be downloaded at: https://www.mdpi.com/article/10.3390/biomedicines10071472/s1, Figure S1: Interaction network of the chemical barrier proteins in serum; Figure S2: Interaction network of the chemical barrier proteins in tears; Figure S3: Interaction network of the chemical barrier proteins in saliva; Figure S4: Interaction network of the chemical barrier proteins in nasal secretion; Figure S5: Interaction network of the chemical barrier proteins in sweat; Figure S6: Interaction network of the chemical barrier proteins in urine; Figure S7: Interaction network of the chemical barrier proteins in cervicovaginal fluid; Figure S8: Interaction network of the chemical barrier proteins in seminal fluid; Figure S9: Interaction network of the chemical barrier proteins in the CSF; Table S1: Proteins involved in the first line of host defense in serum; Table S2: Proteins involved in the first line of host defense in tears; Table S3: Proteins involved in the first line of host defense in saliva; Table S4: Proteins involved in the first line of host defense in the nasal secretion; Table S5: Proteins involved in the first line of host defense in sweat; Table S6: Proteins involved in the first line of host defense in urine; Table S7: Proteins involved in the first line of host defense in CSF; Table S8: Proteins involved in the first line of host defense in cervicovaginal fluid; Table S9: Proteins involved in the first line of host defense in the seminal fluid.
