# Supplementary material for: Chemical Barrier Proteins in Human Body Fluids
Source: Biomedicines. 2022 Jun 22;10(7):1472. doi: 10.3390/biomedicines10071472 (PMC9312486; doi:10.3390/biomedicines10071472)
Supplement: Supplementary file 1 [file biomedicines-10-01472-s001.zip › biomedicines-1773728-supplementary/Supplementary/Supplementary/Supplementary.pdf]

Supplementary material

Supplementary figure captions:

Figure S1: Interaction network of the chemical barrier proteins in serum. Each circle represents a protein and the lines indicate interactions. The lines with an arrow represent activation, blocking lines represent inhibition, and simple lines represent protein–protein interaction. Line color indicates the type of interaction: green color refers to activation, red color to inhibition, blue color to binding, yellow color to co-expression and purple color to catalysis. The proteins are labeled with their gene name.

Figure S2: Interaction network of the chemical barrier proteins in tears. Each circle represents a protein and the lines indicate interactions. The lines with an arrow represent activation, blocking lines represent inhibition, and simple lines represent protein–protein interaction. Line color indicates the type of interaction: green color refers to activation, red color to inhibition, blue color to binding, yellow color to co-expression and purple color to catalysis. The proteins are labeled with their gene name.

Figure S3: Interaction network of the chemical barrier proteins in saliva. Each circle represents a protein and the lines indicate interactions. The lines with an arrow represent activation, blocking lines represent inhibition, and simple lines represent protein–protein interaction. Line color indicates the type of interaction: green color refers to activation, red color to inhibition, blue color to binding, yellow color to co-expression and purple color to catalysis. The proteins are labeled with their gene name.

Figure S4: Interaction network of the chemical barrier proteins in nasal secretion. Each circle represents a protein and the lines indicate interactions. The lines with an arrow represent activation, blocking lines represent inhibition, and simple lines represent protein–protein interaction. Line color indicates the type of interaction: green color refers to activation, red color to inhibition, blue color to binding, yellow color to co-expression and purple color to catalysis. The proteins are labeled with their gene name.

Figure S5: Interaction network of the chemical barrier proteins in sweat. Each circle represents a protein and the lines indicate interactions. The lines with an arrow represent activation, blocking lines represent inhibition, and simple lines represent protein–protein interaction. Line color indicates the type of interaction: green color refers to activation, red color to inhibition, blue color to binding, yellow color to co-expression and purple color to catalysis. The proteins are labeled with their gene name.

Figure S6: Interaction network of the chemical barrier proteins in urine. Each circle represents a protein and the lines indicate interactions. The lines with an arrow represent activation, blocking lines represent inhibition, and simple lines represent protein–protein interaction. Line color indicates the type of interaction: green color refers to activation, red color to inhibition, blue color to binding, yellow color to co-expression and purple color to catalysis. The proteins are labeled with their gene name.

Figure S7: Interaction network of the chemical barrier proteins in cervicovaginal fluid. Each circle represents a protein and the lines indicate interactions. The lines with an arrow represent activation, blocking lines represent inhibition, and simple lines represent protein–protein interaction. Line color indicates the type of interaction: green color refers to activation, red color to inhibition, blue color to binding, yellow color to co-expression and purple color to catalysis. The proteins are labeled with their gene name.

Figure S8: Interaction network of the chemical barrier proteins in the seminal fluid. Each circle represents a protein and the lines indicate interactions. The lines with an arrow represent activation, blocking lines represent inhibition, and simple lines represent protein–protein interaction. Line color indicates the type of interaction: green color refers to activation, red color to inhibition, blue color to binding, yellow color to co-expression and purple color to catalysis. The proteins are labeled with their gene name.

Figure S9: Interaction network of the chemical barrier proteins in CSF. Each circle represents a protein and the lines indicate interactions. The lines with an arrow represent activation, blocking lines represent inhibition, and simple lines represent protein–protein interaction. Line color indicates the type of interaction: green color refers to activation, red color to inhibition, blue color to binding, yellow color to co-expression and purple color to catalysis. The proteins are labeled with their gene name.

Supplementary tables:

Table S1. Proteins involved in the first line of host defense in serum.

Protein name	UniProt entry	Function	Reference
Acrosin	P10323	Serine protease activity	[1]
ADAM DEC1	O15204	Immunomodulatory effect	[2]
Alpha-1-acid glycoprotein 1	P02763	Immunomodulatory effect	[3]
Alpha-1-acid glycoprotein 2	P19652	Immunomodulatory effect	[3]
Alpha-1-antichymotrypsin	P01011	Protease inhibitor	[4]
Alpha-1-antitrypsin	P01009	Protease inhibitor	[5]
Alpha-1B-glycoprotein	P04217	Immunomodulatory effect	[6]
Alpha-2-antiplasmin	P08697	Protease inhibitor	[7]
Alpha-2-HS-glycoprotein	P02765	Anti-inflammatory effect	[8]
Alpha-2-macroglobulin	P01023	Protease inhibitor	[9]
Alpha-2-macroglobulin-like protein 1	A8K2U0	Protease inhibitor	[10]
Alpha-amylase 1A	P0DUB6	Regulation of biofilm formation	[11]
Alpha-amylase 1B	P0DTE7	Regulation of biofilm formation	[11]
Alpha-amylase 1C	P0DTE8	Regulation of biofilm formation	[11]
Alpha-amylase 2B	P19961	Regulation of biofilm formation	[11]
Aminopeptidase B	Q9H4A4	Exopeptidase activity	[12]
Aminopeptidase N	P15144	Exopeptidase activity	[13]
Aminopeptidase O	Q8N6M6	Exopeptidase activity	[14]
Amyloid-beta precursor protein	P05067	Antimicrobial activity	[15]
Angiogenin	P03950	Antimicrobial activity	[16]
Antileukoproteinase	P03973	Protease inhibitor<br>Immunomodulatory effect	[17,18]
Antithrombin-III	P01008	Protease inhibitor	[19]
Apolipoprotein A-I	P02647	Antimicrobial activity	[20]
Apolipoprotein A-II	P02652	Immunomodulatory effect	[21]
Apolipoprotein A-IV	P06727	Immunomodulatory effect	[22]
Apolipoprotein B-100	P04114	Antimicrobial activity	[23]
Apolipoprotein C-III	P02656	Immunomodulatory effect	[24]
Apolipoprotein C-IV	P55056	Immunomodulatory effect	[25]
Apolipoprotein D	P05090	Immunomodulatory effect	[26]
Apolipoprotein E	P02649	Immunomodulatory effect	[27]
Apolipoprotein L1	O14791	Immunomodulatory effect	[28]
Apolipoprotein M	O95445	Immunomodulatory effect	[29]
Arginase-1	P05089	Antifungal activity<br>Immunomodulatory effect	[30,31]
Aspartyl aminopeptidase	Q9ULA0	Exopeptidase activity	[32]
Azurocidin	P20160	Antimicrobial activity	[33]

Bactericidal permeability-increasing protein	P17213	Antimicrobial activity	[33,34]
Beta-2-glycoprotein 1	P02749	Immunomodulatory effect	[35]
Beta-2-microglobulin	P61769	Antimicrobial activity<br>Immunomodulatory effect	[36,37]
Beta-Ala-His dipeptidase	Q96KN2	Carboxypeptidase activity	[38]
Beta-defensin 1	P60022	Antimicrobial activity	[33]
Beta-defensin 118	Q96PH6	Antimicrobial activity	[33]
Beta-defensin 126	Q9BYW3	Antimicrobial activity	[33]
Beta-defensin 129	Q9H1M3	Antimicrobial activity	[33]
Beta-defensin 132	Q7Z7B7	Antimicrobial activity	[33]
Beta-defensin 4A	O15263	Antimicrobial activity	[33]
Beta-hexosaminidase subunit alpha	P06865	Antimicrobial activity	[39]
Beta-hexosaminidase subunit beta	P07686	Antimicrobial activity	[39]
Bone marrow stromal antigen 2	Q10589	Antiviral effect	[40]
BPI fold-containing family A member 1	Q9NP55	Antimicrobial activity	[41]
BPI fold-containing family A member 2	Q96DR5	Antimicrobial activity	[42]
BPI fold-containing family A member 3	Q9BQP9	Antimicrobial activity	[43]
BPI fold-containing family B member 1	Q8TDL5	Antimicrobial activity	[44]
BPI fold-containing family B member 2	Q8N4F0	Antimicrobial activity	[44]
BPI fold-containing family B member 3	P59826	Antimicrobial activity	[45]
BPI fold-containing family B member 4	P59827	Antimicrobial activity	[46]
Brain-specific serine protease 4	Q9GZN4	Serine protease activity	[47]
Calcitonin gene-related peptide 1	P06881	Antimicrobial activity	[48]
Calpain-1 catalytic subunit	P07384	Endopeptidase activity<br>Immunomodulatory effect	[49]
Calpain-10	Q9HC96	Endopeptidase activity<br>Immunomodulatory effect	[49]
Calpain-11	Q9UMQ6	Endopeptidase activity<br>Immunomodulatory effect	[49]
Calpain-12	Q6ZSI9	Endopeptidase activity<br>Immunomodulatory effect	[49]
Calpain-13	Q6MZZ7	Endopeptidase activity<br>Immunomodulatory effect	[49]
Calpain-2 catalytic subunit	P17655	Endopeptidase activity<br>Immunomodulatory effect	[49]
Calpain-3	P20807	Endopeptidase activity<br>Immunomodulatory effect	[49]
Calpain-5	O15484	Endopeptidase activity<br>Immunomodulatory effect	[49]
Calpain-6	Q9Y6Q1	Endopeptidase activity<br>Immunomodulatory effect	[49]
Calpain-7	Q9Y6W3	Endopeptidase activity<br>Immunomodulatory effect	[49]
Calpain-8	A6NHC0	Endopeptidase activity<br>Immunomodulatory effect	[49]
Calpain-9	O14815	Endopeptidase activity<br>Immunomodulatory effect	[49]
Calpastatin	P20810	Protease inhibitor	[50]

Carboxypeptidase A1	P15085	Carboxypeptidase activity	[51]
Carboxypeptidase A2	P48052	Carboxypeptidase activity	[51]
Carboxypeptidase A4	Q9UI42	Carboxypeptidase activity	[51]
Carboxypeptidase A5	Q8WXQ8	Carboxypeptidase activity	[51]
Carboxypeptidase A6	Q8N4T0	Carboxypeptidase activity	[51]
Carboxypeptidase B	P15086	Carboxypeptidase activity	[52]
Carboxypeptidase B2	Q96IY4	Carboxypeptidase activity	[52]
Carboxypeptidase D	O75976	Carboxypeptidase activity	[53]
Carboxypeptidase E	P16870	Carboxypeptidase activity	[53]
Carboxypeptidase M	P14384	Carboxypeptidase activity	[54]
Carboxypeptidase N catalytic chain	P15169	Carboxypeptidase activity	[55]
Carboxypeptidase Q	Q9Y646	Carboxypeptidase activity	[56]
Carcinoembryonic antigen-related cell adhesion molecule 1	P13688	Immunomodulatory effect	[57]
Carcinoembryonic antigen-related cell adhesion molecule 3	P40198	Immunomodulatory effect	[58]
Catalase	P04040	Antimicrobial activity	[59]
Cathelicidin antimicrobial peptide	P49913	Antimicrobial activity	[33]
Cathepsin B	P07858	Endopeptidase activity	[60]
Cathepsin D	P07339	Endopeptidase activity	[60]
Cathepsin F	Q9UBX1	Endopeptidase activity	[60]
Cathepsin G	P08311	Endopeptidase activity	[60]
Cathepsin K	P43235	Endopeptidase activity	[60]
Cathepsin L2	O60911	Endopeptidase activity	[60]
Cathepsin O	P43234	Endopeptidase activity	[60]
Cathepsin S	P25774	Endopeptidase activity	[60]
Cathepsin W	P56202	Endopeptidase activity	[60]
Cathepsin Z	Q9UBR2	Endopeptidase activity	[60]
Cell surface glycoprotein MUC18	P43121	Immunomodulatory effect	[61]
Ceruloplasmin	P00450	Cu^{2+} sequestering activity	[62]
Chitinase-3-like protein 1	P36222	Antimicrobial activity	[63]
Chitotriosidase-1	Q13231	Antifungal activity	[64]
Chromogranin-A	P10645	Processed forms have anti-microbial activity	[65]
Clusterin	P10909	Immunomodulatory effect	[66]
Collagen alpha-1(XII) chain	Q99715	Immunomodulatory effect	[67]
Core histone macro-H2A.1	O75367	Antimicrobial activity	[68]
Core histone macro-H2A.2	Q9P0M6	Antimicrobial activity	[68]
Corticosteroid-binding globulin	P08185	Protease inhibitor	[69]
C-reactive protein	P02741	Acute phase protein	[70]
Cystatin-A	P01040	Protease inhibitor	[71]
Cystatin-B	P04080	Protease inhibitor	[71]
Cystatin-C	P01034	Protease inhibitor	[71]
Cystatin-D	P28325	Protease inhibitor	[71]

Cystatin-F	O76096	Protease inhibitor	[71]
Cystatin-M	Q15828	Protease inhibitor	[71]
Cystatin-S	P01036	Protease inhibitor	[71]
Cystatin-SA	P09228	Protease inhibitor	[71]
Cystatin-SN	P01037	Protease inhibitor	[71]
Cytosol aminopeptidase	P28838	Aminopeptidase activity	[72]
Cytosolic carboxypeptidase 1	Q9UPW5	Carboxypeptidase activity	[73]
Cytosolic carboxypeptidase 3	Q8NEM8	Carboxypeptidase activity	[74]
Cytosolic non-specific dipeptidase	Q96KP4	Carboxypeptidase activity	[75]
Defensin-5	Q01523	Antimicrobial activity	[33]
Defensin-6	Q01524	Antimicrobial activity	[33]
Deleted in malignant brain tumors 1 protein	Q9UGM3	Immunomodulatory effect Antimicrobial activity	[76,77]
Deoxyribonuclease-1	P24855	Endonuclease activity	[78]
Dermcidin	P81605	Antimicrobial activity	[33]
Dipeptidase 1	P16444	Carboxypeptidase activity	[79]
Dipeptidase 2	Q9H4A9	Carboxypeptidase activity	[80]
Dipeptidase 3	Q9H4B8	Carboxypeptidase activity	[80]
Dipeptidyl peptidase 1	P53634	Carboxypeptidase activity	[81]
Dipeptidyl peptidase 2	Q9UHL4	Carboxypeptidase activity	[82]
Dipeptidyl peptidase 3	Q9NY33	Carboxypeptidase activity	[83]
Dipeptidyl peptidase 4	P27487	Carboxypeptidase activity	[84]
Dipeptidyl peptidase 9	Q86TI2	Carboxypeptidase activity	[85]
Disintegrin and metalloproteinase domain-containing protein 10	O14672	Metalloendopeptidase activity	[86]
Disintegrin and metalloproteinase domain-containing protein 11	O75078	Metalloendopeptidase activity	[86]
Disintegrin and metalloproteinase domain-containing protein 12	O43184	Metalloendopeptidase activity	[86]
Disintegrin and metalloproteinase domain-containing protein 15	Q13444	Metalloendopeptidase activity	[86]
Disintegrin and metalloproteinase domain-containing protein 17	P78536	Metalloendopeptidase activity	[86]
Disintegrin and metalloproteinase domain-containing protein 18	Q9Y3Q7	Metalloendopeptidase activity	[86]
Disintegrin and metalloproteinase domain-containing protein 19	Q9H013	Metalloendopeptidase activity	[86]
Disintegrin and metalloproteinase domain-containing protein 2	Q99965	Metalloendopeptidase activity	[86]
Disintegrin and metalloproteinase domain-containing protein 20	O43506	Metalloendopeptidase activity	[86]
Disintegrin and metalloproteinase domain-containing protein 22	Q9P0K1	Metalloendopeptidase activity	[86]
Disintegrin and metalloproteinase	O75077	Metalloendopeptidase	[86]

domain-containing protein 23		activity	
Disintegrin and metalloproteinase domain-containing protein 28	Q9UKQ2	Metalloendopeptidase activity	[86]
Disintegrin and metalloproteinase domain-containing protein 29	Q9UKF5	Metalloendopeptidase activity	[86]
Disintegrin and metalloproteinase domain-containing protein 30	Q9UKF2	Metalloendopeptidase activity	[86]
Disintegrin and metalloproteinase domain-containing protein 32	Q8TC27	Metalloendopeptidase activity	[86]
Disintegrin and metalloproteinase domain-containing protein 33	Q9BZ11	Metalloendopeptidase activity	[86]
Disintegrin and metalloproteinase domain-containing protein 7	Q9H2U9	Metalloendopeptidase activity	[86]
Disintegrin and metalloproteinase domain-containing protein 8	P78325	Metalloendopeptidase activity	[86]
Disintegrin and metalloproteinase domain-containing protein 9	Q13443	Metalloendopeptidase activity	[86]
Drebrin-like protein	Q9UJU6	Immunomodulatory effect	[87]
Elafin	P19957	Protease inhibitor	[88]
Endoplasmic reticulum aminopeptidase 1	Q9NZ08	Aminopeptidase activity	[89]
Endoplasmic reticulum aminopeptidase 2	Q6P179	Aminopeptidase activity	[89]
Eosinophil cationic protein	P12724	Antimicrobial activity	[90]
Eosinophil peroxidase	P11678	Antimicrobial activity	[91]
Extracellular glycoprotein lacritin	Q9GZZ8	Antimicrobial activity	[92]
Fatty acid-binding protein 4	P15090	Immunomodulatory effect	[93]
Fatty acid-binding protein 5	Q01469	Immunomodulatory effect	[94]
Fibrinogen alpha chain	P02671	Immunomodulatory effect	[95]
Fibrinogen beta chain	P02675	Immunomodulatory effect	[95]
Fibrinogen gamma chain	P02679	Immunomodulatory effect	[95]
Fibrocystin-L	Q86WI1	Immunomodulatory effect	[96]
Fibroleukin	Q14314	Immunomodulatory effect	[97]
Folliculin-interacting protein 1	Q8TF40	Immunomodulatory effect	[98]
Furin	P09958	Serine protease activity	[99]
FYN-binding protein 1	O15117	Immunomodulatory effect	[100]
Galectin-1	P09382	Immunomodulatory effect	[101]
Galectin-10	Q05315	Immunomodulatory effect	[102]
Galectin-3	P17931	Immunomodulatory effect	[103]
Galectin-3-binding protein	Q08380	Antimicrobial activity Immunomodulatory effect	[104,105]
Galectin-7	P47929	Immunomodulatory effect	[106]
Galectin-9	O00182	Immunomodulatory effect	[107]
Gastricsin	P20142	Aspartic-type endopeptidase activity	[108]
Gelsolin	P06396	Processed from has antimi-	[109]

		crobial activity	
Glia-derived nexin	P07093	Protease inhibitor	[110]
Glucose-6-phosphate isomerase	P06744	Induces immunoglobulin secretion	[111]
Glutamate carboxypeptidase 2	Q04609	Carboxypeptidase activity	[112]
Glutamyl aminopeptidase	Q07075	Aminopeptidase activity	[113]
Glutathione S-transferase omega-1	P78417	Immunomodulatory effect	[114]
Glutathione S-transferase P	P09211	Immunomodulatory effect	[115]
Glyceraldehyde-3-phosphate dehydrogenase	P04406	Immunomodulatory effect	[116]
Granzyme A	P12544	Serine protease activity	[117]
Granzyme B	P10144	Serine protease activity	[117]
Granzyme H	P20718	Serine protease activity	[117]
Granzyme K	P49863	Serine protease activity	[117]
Granzyme M	P51124	Serine protease activity	[117]
Growth-regulated alpha protein	P09341	Antimicrobial activity	[118]
Guanylate-binding protein 1	P32455	Immunomodulatory effect	[119]
Guanylate-binding protein 2	P32456	Antiviral effect	[120]
Guanylate-binding protein 4	Q96PP9	Immunomodulatory effect	[119]
Guanylate-binding protein 5	Q96PP8	Immunomodulatory effect	[121]
Haptoglobin	P00738	Immunomodulatory effect<br>Iron sequestrating	[122]
Haptoglobin-related protein	P00739	Anti-parasitic effect	[123]
Heme-binding protein 1	Q9NRV9	Heme/iron sequestration	[124]
Heme-binding protein 2	Q9Y5Z4	Heme/iron sequestration	[124]
Hemoglobin subunit alpha	P69905	Processed forms (hemocidins) have antimicrobial activity	[125]
Hemoglobin subunit beta	P68871	Processed forms (hemocidins) have antimicrobial activity	[125]
Hemopexin	P02790	Antibacterail effect<br>Anti-inflammatory effect	[126]
Heparin cofactor 2	P05546	Protease inhibitor	[127]
Hepcidin	P81172	Antimicrobial activity<br>Iron sequestration	[128,129]
High mobility group protein B1	P09429	Immunomodulatory effect	[130]
High mobility group protein B2	P26583	Antimicrobial activity	[131]
High mobility group protein B3	O15347	Immunomodulatory effect	[132]
Histidine-rich glycoprotein	P04196	Antimicrobial activity	[133]
Histone H1.0	P07305	Antimicrobial activity	[134]
Histone H1.1	Q02539	Antimicrobial activity	[134]
Histone H1.10	Q92522	Antimicrobial activity	[134]
Histone H1.2	P16403	Antimicrobial activity	[134]
Histone H1.3	P16402	Antimicrobial activity	[134]
Histone H1.4	P10412	Antimicrobial activity	[134]

Histone H1.5	P16401	Antimicrobial activity	[134]
Histone H1t	P22492	Antimicrobial activity	[134]
Histone H2A type 1-B/E	P04908	Antimicrobial activity	[134]
Histone H2A type 2-A	Q6FI13	Antimicrobial activity	[134]
Histone H2A type 3	Q7L7L0	Antimicrobial activity	[134]
Histone H2A.Z	P0C0S5	Antimicrobial activity	[134]
Histone H2AX	P16104	Antimicrobial activity	[134]
Histone H2B type 1-A	Q96A08	Antimicrobial activity	[134]
Histone H2B type 1-B	P33778	Antimicrobial activity	[134]
Histone H2B type 1-C/E/F/G/I	P62807	Antimicrobial activity	[134]
Histone H2B type 1-H	Q93079	Antimicrobial activity	[134]
Histone H2B type 1-K	O60814	Antimicrobial activity	[134]
Histone H2B type 1-L	Q99880	Antimicrobial activity	[134]
Histone H3.1	P68431	Antimicrobial activity	[134]
Histone H3.2	Q71DI3	Antimicrobial activity	[134]
Histone H3.3	P84243	Antimicrobial activity	[134]
Histone H3-7	Q5TEC6	Antimicrobial activity	[134]
Histone H4	P62805	Antimicrobial activity	[134]
Inter-alpha-trypsin inhibitor heavy chain H1	P19827	Protease inhibitor	[135]
Inter-alpha-trypsin inhibitor heavy chain H2	P19823	Protease inhibitor	[135]
Inter-alpha-trypsin inhibitor heavy chain H3	Q06033	Protease inhibitor	[135]
Inter-alpha-trypsin inhibitor heavy chain H4	Q14624	Protease inhibitor	[135]
Inter-alpha-trypsin inhibitor heavy chain H5	Q86UX2	Protease inhibitor	[135]
Inter-alpha-trypsin inhibitor heavy chain H6	Q6UXX5	Protease inhibitor	[135]
Interferon-induced 35 kDa protein	P80217	Immunomodulatory effect	[136]
Interferon-stimulated gene 20 kDa protein	Q96AZ6	Antiviral effect	[137]
Kallikrein-11	Q9UBX7	Serine protease activity	[138]
Kallikrein-12	Q9UKR0	Serine protease activity	[138]
Kallikrein-13	Q9UKR3	Serine protease activity	[138]
Kallikrein-14	Q9P0G3	Serine protease activity	[138]
Kallikrein-15	Q9H2R5	Serine protease activity	[138]
Kallikrein-2	P20151	Serine protease activity	[138]
Kallikrein-3	P07288	Serine protease activity	[138]
Kallikrein-5	Q9Y337	Serine protease activity<br>Processing the maturation of LL-37 cathelicidin	[138,139]
Kallikrein-6	Q92876	Serine protease activity	[138]
Kallikrein-7	P49862	Serine protease activity<br>Processing the maturation of LL-37 cathelicidin	[138]
Kallikrein-8	O60259	Serine protease activity	[138]
Kininogen-1	P01042	Antimicrobial activity	[140,141]
Kunitz-type protease inhibitor 1	O43278	Protease inhibitor	[142]

Kunitz-type protease inhibitor 2	O43291	Protease inhibitor	[142]
Lactoperoxidase	P22079	Antimicrobial activity	[143]
Lactotransferrin	P02788	Antimicrobial activity<br>Iron sequestration	[33]
Legumain	Q99538	Endopeptidase activity	[144]
Leukocyte elastase inhibitor	P30740	Protease inhibitor	[145]
Lipocalin-1	P31025	Immunomodulatory effect<br>Iron sequestration	[146,147]
Lipocalin-2	P80188	Immunomodulatory effect<br>Iron sequestration	[146,147]
Lipopolysaccharide-binding protein	P18428	Immunomodulatory effect	[148]
Liver-expressed antimicrobial peptide 2	Q969E1	Antimicrobial activity	[149]
Lymphotactin	P47992	Antimicrobial activity	[150]
Lysozyme C	P61626	Antimicrobial activity	[33]
Macrophage migration inhibitory factor	P14174	Antimicrobial activity	[151]
Major vault protein	Q14764	Immunomodulatory effect	[152]
Mammaglobin-B	O75556	Immunomodulatory effect	[153]
Mast cell carboxypeptidase A	P15088	Carboxypeptidase activity	[154]
Matrix metalloproteinase-9	P14780	Metalloprotease activity	[155]
Melanotransferrin	P08582	Iron sequestration	[156]
Metalloproteinase inhibitor 1	P01033	Protease Inhibitor	[157]
Metalloproteinase inhibitor 2	P16035	Protease Inhibitor	[157]
Metalloproteinase inhibitor 4	Q99727	Protease Inhibitor	[157]
Midkine	P21741	Immunomodulatory effect	[158]
Moesin	P26038	Immunomodulatory effect	[159]
Mucin-1	P15941	Antimicrobial activity	[160]
Mucin-13	Q9H3R2	Antimicrobial activity	[160]
Mucin-15	Q8N387	Antimicrobial activity	[160]
Mucin-16	Q8WXI7	Antimicrobial activity	[160]
Mucin-17	Q685J3	Antimicrobial activity	[160]
Mucin-2	Q02817	Antimicrobial activity	[160]
Mucin-4	Q99102	Antimicrobial activity	[160]
Mucin-5AC	P98088	Antimicrobial activity	[160]
Mucin-5B	Q9HC84	Antimicrobial activity	[160]
Mucin-6	Q6W4X9	Antimicrobial activity	[160]
Mucin-7	Q8TAX7	Antimicrobial activity	[160]
Myeloblastin	P24158	Serine protease activity	[161]
Myeloperoxidase	P05164	Antimicrobial activity	[162]
Myoglobin	P02144	Processed forms (hemocidins) have antimicrobial activity	[163]
N-acetylmuramoyl-L-alanine amidase	Q96PD5	Antimicrobial activity	[164]
Neprilysin	P08473	Endopeptidase activity	[165]
Neutrophil collagenase	P22894	Endopeptidase activity<br>Immunomodulatory effect	[166]

Neutrophil defensin 1	P59665	Antimicrobial activity	[33]
Neutrophil defensin 3	P59666	Antimicrobial activity	[33]
Neutrophil defensin 4	P12838	Antimicrobial activity	[33]
Neutrophil elastase	P08246	Serine protease activity	[167]
Nicotinamide phosphoribosyltransferase	P43490	Immunomodulatory effect	[168]
Non-histone chromosomal protein HMG-17	P05204	Antimicrobial activity	[169]
Non-secretory ribonuclease	P10153	Ribonuclease activity	[170]
Opiorphin prepropeptide	Q99935	Protease inhibitor	[171]
Peptidase inhibitor 16	Q6UXB8	Protease inhibitor	[172]
Peptidoglycan recognition protein 1	O75594	Antimicrobial activity	[173]
Perforin-1	P14222	Antimicrobial activity	[174]
Phospholipase B-like 1	Q6P4A8	Suggested antimicrobial activity	[175]
Pigment epithelium-derived factor	P36955	Protease inhibitor	[176]
Plasma kallikrein	P03952	Serine protease activity	[138]
Plasma serine protease inhibitor	P05154	Protease inhibitor	[177]
Plastin-2	P13796	Immunomodulatory effect	[178]
Poly(rC)-binding protein 1	Q15365	Antiviral effect	[179]
Poly(rC)-binding protein 2	Q15366	Antiviral effect	[180]
Pregnancy zone protein	P20742	Protease inhibitor	[181]
Pro-adrenomedullin	P35318	Antimicrobial activity<br>Immunomodulatory effect	[182,183]
Pro-cathepsin H	P09668	Endopeptidase activity	[60]
Procathepsin L	P07711	Endopeptidase activity	[60]
Progranulin	P28799	Immunomodulatory effect	[184]
Prolactin-inducible protein	P12273	Aspartic-type endopeptidase activity<br>Modulates the activity of Zn-α2 glycoprotein	[185,186]
Proline-rich protein 11	Q96HE9	Antimicrobial activity	[187]
Proline-rich protein 14	Q9BWN1	Antimicrobial activity	[187]
Proline-rich protein 18	Q8N4B5	Antimicrobial activity	[187]
Proline-rich protein 30	Q53SZ7	Antimicrobial activity	[187]
Proline-rich protein 4	Q16378	Antimicrobial activity	[187]
Proline-rich protein 5	P85299	Antimicrobial activity	[187]
Prolyl endopeptidase	P48147	Endopeptidase activity	[188]
Pro-opiomelanocortin	P01189	Antimicrobial activity	[189]
Prosalusin	Q8N2E6	Antimicrobial activity	[190]
Prosaposin	P07602	Processed forms has antimicrobial effect	[191]
Prostasin	Q16651	Serine protease activity	[192]
Protein AMBP	P02760	Protease inhibitor	[193]
Protein FAM3A	P98173	Antifungal effect	[194]
Protein S100-A1	P23297	Immunomodulatory effect	[195]
Protein S100-A10	P60903	Immunomodulatory effect	[196]

Protein S100-A11	P31949	Immunomodulatory effect	[197]
Protein S100-A12	P80511	Immunomodulatory effect	[196]
Protein S100-A13	Q99584	Immunomodulatory effect	[198]
Protein S100-A14	Q9HCY8	Immunomodulatory effect	[199]
Protein S100-A2	P29034	Immunomodulatory effect	[200]
Protein S100-A4	P26447	Immunomodulatory effect	[196]
Protein S100-A6	P06703	Immunomodulatory effect	[201]
Protein S100-A7	P31151	Immunomodulatory effect	[196]
Protein S100-A8	P05109	Immunomodulatory effect	[196]
Protein S100-A9	P06702	Immunomodulatory effect	[196]
Protein S100-B	P04271	Immunomodulatory effect	[196]
Protein S100-P	P25815	Immunomodulatory effect	[196]
Protein WFDC9	Q8NEX5	Protease inhibitor	[202]
Puromycin-sensitive aminopeptidase	P55786	Aminopeptidase activity	[203]
RelA-associated inhibitor	Q8WUF5	Antiviral effect Immunomodulatory effect	[204]
Retroviral-like aspartic protease 1	Q53RT3	Aspartic-type endopeptidase activity	[205]
Ribonuclease 4	P34096	Ribonuclease activity	[170]
Ribonuclease 8	Q8TDE3	Ribonuclease activity	[170]
Ribonuclease K6	Q93091	Ribonuclease activity	[170]
Ribonuclease pancreatic	P07998	Ribonuclease activity	[170]
Ribonuclease T2	O00584	Ribonuclease activity	[170]
Secreted Ly-6/uPAR-related protein 1	P55000	Immunomodulatory effect	[206]
Secretoglobin family 1D member 2	O95969	Immunomodulatory effect	[207]
Secretoglobin family 3A member 1	Q96QR1	Immunomodulatory effect	[208]
Secretoglobin family 3A member 2	Q96PL1	Immunomodulatory effect	[207]
Semenogelin-1	P04279	Processed forms has antimicrobial activity	[209–211]
Semenogelin-2	Q02383	Processed forms has antimicrobial activity	[209–211]
Serine protease 1	P07477	Serine protease activity	[212]
Serine protease 23	O95084	Serine protease activity	[212]
Serine protease 27	Q9BQR3	Serine protease activity	[212]
Serine protease 57	Q6UWY2	Serine protease activity	[212]
Serine protease HTRA1	Q92743	Serine protease activity	[212]
Serine protease HTRA2	O43464	Serine protease activity	[212]
Serine protease HTRA3	P83110	Serine protease activity	[212]
Serine protease HTRA4	P83105	Serine protease activity	[212]
Serine protease inhibitor Kazal-type 1	P00995	Protease inhibitor	[213]
Serine protease inhibitor Kazal-type 5	Q9NQ38	Protease inhibitor	[213]
Serine protease inhibitor Kazal-type 6	Q6UWN8	Protease inhibitor	[213]
Serine protease inhibitor Kazal-type 7	P58062	Protease inhibitor	[213]
Serotransferrin	P02787	Iron sequestration	[214]
Serpin B10	P48595	Protease inhibitor	[215]

Serpin B11	Q96P15	Protease inhibitor	[215]
Serpin B12	Q96P63	Protease inhibitor	[215]
Serpin B13	Q9UIV8	Protease inhibitor	[215]
Serpin B3	P29508	Protease inhibitor	[215]
Serpin B4	P48594	Protease inhibitor	[215]
Serpin B5	P36952	Protease inhibitor	[215]
Serpin B6	P35237	Protease inhibitor	[215]
Serpin B7	O75635	Protease inhibitor	[215]
Serpin B8	P50452	Protease inhibitor	[215]
Serpin B9	P50453	Protease inhibitor	[215]
Serum amyloid A-1 protein	P0DJI8	Immunomodulatory effect	[216]
Serum amyloid A-2 protein	P0DJI9	Immunomodulatory effect	[216]
Serum amyloid A-4 protein	P35542	Immunomodulatory effect	[216]
Serum amyloid P-component	P02743	Antiviral effect	[217]
Sialomucin core protein 24	Q04900	Immunomodulatory effect	[218]
Small proline-rich protein 3	Q9UBC9	Antimicrobial effect	[187]
Syntenin-1	O00560	Immunomodulatory effect	[219]
T-cell immunomodulatory protein	Q8TB96	Immunomodulatory effect	[220]
Thioredoxin domain-containing protein 17	Q9BRA2	Immunomodulatory effect	[221]
Thymosin beta-10	P63313	Antimicrobial activity	[222]
Thymosin beta-4	P62328	Antimicrobial activity	[222]
Thyroxine-binding globulin	P05543	Protease inhibitor	[223]
Toll-interacting protein	Q9H0E2	Immunomodulatory effect	[224]
Transgelin	Q01995	Immunomodulatory effect	[225]
Transgelin-2	P37802	Immunomodulatory effect	[226]
Transmembrane protease serine 11A	Q6ZMR5	Serine protease activity	[227]
Transmembrane protease serine 11D	O60235	Serine protease activity	[228]
Transmembrane protease serine 11E	Q9UL52	Serine protease activity	[229]
Triokinase/FMN cyclase	Q3LXA3	Immunomodulatory effect	[230]
Tripeptidyl-peptidase 1	O14773	Serine protease activity	[231]
Tripeptidyl-peptidase 2	P29144	Serine protease activity	[232]
Trypsin-2	P07478	Serine protease activity	[233]
Trypsin-3	P35030	Serine protease activity	[234]
Tryptase alpha/beta-1	Q15661	Serine protease activity	[235]
Tryptase beta-2	P20231	Serine protease activity	[234]
Tryptase delta	Q9BZJ3	Serine protease activity	[234]
Uromodulin	P07911	Antimicrobial activity	[236,237]
Uteroglobin	P11684	Immunomodulatory effect	[238]
Vitamin D-binding protein	P02774	Immunomodulatory effect	[239]
WAP four-disulfide core domain protein 1	Q9HC57	Protease inhibitor	[240]
WAP four-disulfide core domain protein 2	Q14508	Protease inhibitor	[240]
WAP four-disulfide core domain protein 6	Q9BQY6	Protease inhibitor	[240]
WAP four-disulfide core domain protein 8	Q8IUA0	Protease inhibitor	[240]

WAP, Kazal, immunoglobulin, Kunitz and NTR domain-containing protein 2	Q8TEU8	Protease inhibitor	[240]
Xaa-Pro aminopeptidase 1	Q9NQW7	Aminopeptidase activity	[241]
Xaa-Pro aminopeptidase 2	O43895	Aminopeptidase activity	[241]
Xaa-Pro dipeptidase	P12955	Carboxypeptidase activity	[242]
Zinc-alpha-2-glycoprotein	P25311	Immunomodulatory effect	[243]
Zymogen granule membrane protein 16	O60844	Antimicrobial activity	[244]
Zymogen granule protein 16 homolog B	Q96DA0	Antimicrobial activity	[245]

Table S2. Proteins involved in the first line of host defense in tears.

Protein name	UniProt entry	Function	Reference
Alpha-1-acid glycoprotein 1	P02763	Immunomodulatory effect	[3]
Alpha-1-acid glycoprotein 2	P19652	Immunomodulatory effect	[3]
Alpha-1-antichymotrypsin	P01011	Protease inhibitor	[4]
Alpha-1-antitrypsin	P01009	Protease inhibitor	[5]
Alpha-1B-glycoprotein	P04217	Immunomodulatory effect	[6]
Alpha-2-antiplasmin	P08697	Protease inhibitor	[7]
Alpha-2-HS-glycoprotein	P02765	Anti-inflammatory effect	[8]
Alpha-amylase 1A	P0DUB6	Regulation of biofilm formation	[11]
Alpha-amylase 1B	P0DTE7	Regulation of biofilm formation	[11]
Alpha-amylase 1C	P0DTE8	Regulation of biofilm formation	[11]
Aminopeptidase B	Q9H4A4	Exopeptidase activity	[12]
Aminopeptidase N	P15144	Exopeptidase activity	[13]
Amyloid-beta precursor protein	P05067	Antimicrobial activity	[15]
Angiogenin	P03950	Antimicrobial activity	[16]
Antileukoproteinase	P03973	Protease inhibitor<br>Immunomodulatory effect	[17,18]
Antithrombin-III	P01008	Protease inhibitor	[19]
Apolipoprotein A-I	P02647	Antimicrobial activity	[20]
Apolipoprotein A-II	P02652	Immunomodulatory effect	[21]
Apolipoprotein A-IV	P06727	Immunomodulatory effect	[22]
Apolipoprotein B-100	P04114	Antimicrobial activity	[23]
Apolipoprotein C-III	P02656	Immunomodulatory effect	[24]
Apolipoprotein D	P05090	Immunomodulatory effect	[26]
Apolipoprotein E	P02649	Immunomodulatory effect	[27]
Apolipoprotein L1	O14791	Immunomodulatory effect	[28]
Arginase-1	P05089	Antifungal activity<br>Immunomodulatory effect	[30,31]
Aspartyl aminopeptidase	Q9ULA0	Exopeptidase activity	[32]
Azurocidin	P20160	Antimicrobial activity	[33]

Bactericidal permeability-increasing protein	P17213	Antimicrobial activity	[33,34]
Beta-2-glycoprotein 1	P02749	Immunomodulatory effect	[35]
Beta-2-microglobulin	P61769	Antimicrobial activity<br>Immunomodulatory effect	[36,37]
Beta-hexosaminidase subunit alpha	P06865	Antimicrobial activity	[39]
Beta-hexosaminidase subunit beta	P07686	Antimicrobial activity	[39]
BPI fold-containing family B member 2	Q8N4F0	Antimicrobial activity	[44]
Calcitonin gene-related peptide 1	P06881	Antimicrobial activity	[48]
Calpain-1 catalytic subunit	P07384	Endopeptidase activity<br>Immunomodulatory effect	[49]
Calpain-13	Q6MZZ7	Endopeptidase activity<br>Immunomodulatory effect	[49]
Calpain-2 catalytic subunit	P17655	Endopeptidase activity<br>Immunomodulatory effect	[49]
Calpain-7	Q9Y6W3	Endopeptidase activity<br>Immunomodulatory effect	[49]
Calpastatin	P20810	Protease inhibitor	[50]
Carboxypeptidase B2	Q96IY4	Carboxypeptidase activity	[52]
Carboxypeptidase D	O75976	Carboxypeptidase activity	[53]
Carboxypeptidase Q	Q9Y646	Carboxypeptidase activity	[56]
Carcinoembryonic antigen-related cell adhesion molecule 1	P13688	Immunomodulatory effect	[57]
Catalase	P04040	Antimicrobial activity	[59]
Cathelicidin antimicrobial peptide	P49913	Antimicrobial activity	[33]
Cathepsin B	P07858	Endopeptidase activity	[60]
Cathepsin D	P07339	Endopeptidase activity	[60]
Cathepsin F	Q9UBX1	Endopeptidase activity	[60]
Cathepsin G	P08311	Endopeptidase activity	[60]
Cathepsin S	P25774	Endopeptidase activity	[60]
Cathepsin Z	Q9UBR2	Endopeptidase activity	[60]
Ceruloplasmin	P00450	Cu^{2+} sequestering activity	[62]
Clusterin	P10909	Immunomodulatory effect	[66]
Corticosteroid-binding globulin	P08185	Protease inhibitor	[69]
Cystatin-B	P04080	Protease inhibitor	[71]
Cystatin-C	P01034	Protease inhibitor	[71]
Cystatin-D	P28325	Protease inhibitor	[71]
Cystatin-S	P01036	Protease inhibitor	[71]
Cystatin-SA	P09228	Protease inhibitor	[71]
Cystatin-SN	P01037	Protease inhibitor	[71]
Cytosol aminopeptidase	P28838	Aminopeptidase activity	[72]
Cytosolic non-specific dipeptidase	Q96KP4	Carboxypeptidase activity	[75]
Deleted in malignant brain tumors 1 protein	Q9UGM3	Immunomodulatory effect<br>Antimicrobial activity	[76,77]
Dermcidin	P81605	Antimicrobial activity	[33]
Dipeptidyl peptidase 1	P53634	Carboxypeptidase activity	[81]

Dipeptidyl peptidase 2	Q9UHL4	Carboxypeptidase activity	[82]
Dipeptidyl peptidase 3	Q9NY33	Carboxypeptidase activity	[83]
Dipeptidyl peptidase 4	P27487	Carboxypeptidase activity	[84]
Disintegrin and metalloproteinase domain-containing protein 10	O14672	Metalloendopeptidase activity	[86]
Drebrin-like protein	Q9UJU6	Immunomodulatory effect	[87]
Endoplasmic reticulum aminopeptidase 1	Q9NZ08	Aminopeptidase activity	[89]
Eosinophil cationic protein	P12724	Antimicrobial activity	[90]
Extracellular glycoprotein lacritin	Q9GZZ8	Antimicrobial activity	[92]
Fatty acid-binding protein 5	Q01469	Immunomodulatory effect	[94]
FAU ubiquitin-like and ribosomal protein S30	P62861	Antimicrobial activity	[95]
Fibrinogen alpha chain	P02671	Immunomodulatory effect	[95]
Fibrinogen beta chain	P02675	Immunomodulatory effect	[95]
Fibrinogen gamma chain	P02679	Immunomodulatory effect	[95]
Furin	P09958	Serine protease activity	[99]
Galectin-3	P17931	Immunomodulatory effect	[103]
Galectin-3-binding protein	Q08380	Antimicrobial activity Immunomodulatory effect	[104,105]
Galectin-7	P47929	Immunomodulatory effect	[106]
Gelsolin	P06396	Processed from has antimicrobial activity	[109]
Glucose-6-phosphate isomerase	P06744	Induces immunoglobulin secretion	[111]
Glutamate carboxypeptidase 2	Q04609	Carboxypeptidase activity	[112]
Glutathione S-transferase omega-1	P78417	Immunomodulatory effect	[114]
Glutathione S-transferase P	P09211	Immunomodulatory effect	[115]
Glyceraldehyde-3-phosphate dehydrogenase	P04406	Immunomodulatory effect	[116]
Growth-regulated alpha protein	P09341	Antimicrobial activity	[118]
Guanylate-binding protein 1	P32455	Immunomodulatory effect	[119]
Guanylate-binding protein 2	P32456	Antiviral effect	[120]
Haptoglobin	P00738	Immunomodulatory effect Iron sequestrating	[122]
Haptoglobin-related protein	P00739	Anti-parasitic effect	[123]
Heme-binding protein 1	Q9NRV9	Heme/iron sequestration	[124]
Heme-binding protein 2	Q9Y5Z4	Heme/iron sequestration	[124]
Hemoglobin subunit alpha	P69905	Processed forms (hemocidins) have antimicrobial activity	[125]
Hemoglobin subunit beta	P68871	Processed forms (hemocidins) have antimicrobial activity	[125]
Hemopexin	P02790	Antibacterail effect Anti-inflammatory effect	[126]
Heparin cofactor 2	P05546	Protease inhibitor	[127]

High mobility group protein B1	P09429	Immunomodulatory effect	[130]
Histatin-1	P15515	Antimicrobial activity	[246]
Histidine-rich glycoprotein	P04196	Antimicrobial activity	[133]
Histone H1.2	P16403	Antimicrobial activity	[134]
Histone H1.5	P16401	Antimicrobial activity	[134]
Histone H2B type 3-B	Q8N257	Antimicrobial activity	[134]
Histone H4	P62805	Antimicrobial activity	[134]
Inter-alpha-trypsin inhibitor heavy chain H1	P19827	Protease inhibitor	[135]
Inter-alpha-trypsin inhibitor heavy chain H2	P19823	Protease inhibitor	[135]
Inter-alpha-trypsin inhibitor heavy chain H3	Q06033	Protease inhibitor	[135]
Inter-alpha-trypsin inhibitor heavy chain H4	Q14624	Protease inhibitor	[135]
Interferon-stimulated gene 20 kDa protein	Q96AZ6	Antiviral effect	[137]
Kininogen-1	P01042	Antimicrobial activity	[140,141]
Kunitz-type protease inhibitor 1	O43278	Protease inhibitor	[142]
Lactoperoxidase	P22079	Antimicrobial activity	[143]
Lactotransferrin	P02788	Antimicrobial activity<br>Iron sequestration	[33]
Legumain	Q99538	Endopeptidase activity	[144]
Leukocyte elastase inhibitor	P30740	Protease inhibitor	[145]
Lipocalin-1	P31025	Immunomodulatory effect<br>Iron sequestration	[146,147]
Lipocalin-2	P80188	Immunomodulatory effect<br>Iron sequestration	[146,147]
Lysozyme C	P61626	Antimicrobial activity	[33]
Macrophage migration inhibitory factor	P14174	Antimicrobial activity	[151]
Major vault protein	Q14764	Immunomodulatory effect	[152]
Mammaglobin-B	O75556	Immunomodulatory effect	[153]
Matrix metalloproteinase-9	P14780	Metalloprotease activity	[155]
Melanotransferrin	P08582	Iron sequestration	[156]
Metalloproteinase inhibitor 1	P01033	Protease Inhibitor	[157]
Metalloproteinase inhibitor 2	P16035	Protease Inhibitor	[157]
Moesin	P26038	Immunomodulatory effect	[159]
Mucin-1	P15941	Antimicrobial activity	[160]
Mucin-16	Q8WXI7	Antimicrobial activity	[160]
Mucin-4	Q99102	Antimicrobial activity	[160]
Mucin-5AC	P98088	Antimicrobial activity	[160]
Mucin-7	Q8TAX7	Antimicrobial activity	[160]
Myeloblastin	P24158	Serine protease activity	[161]
Myeloperoxidase	P05164	Antimicrobial activity	[162]
Myoglobin	P02144	Processed forms (hemo-cidins) have antimicrobial activity	[163]
N-acetylmuramoyl-L-alanine amidase	Q96PD5	Antimicrobial activity	[164]
Neutrophil defensin 1	P59665	Antimicrobial activity	[33]
Neutrophil defensin 3	P59666	Antimicrobial activity	[33]

Neutrophil elastase	P08246	Serine protease activity	[167]
Nicotinamide phosphoribosyltransferase	P43490	Immunomodulatory effect	[168]
Opiorphin prepropeptide	Q99935	Protease inhibitor	[171]
Peptidoglycan recognition protein 1	O75594	Antimicrobial activity	[173]
Peptidoglycan recognition protein 3	Q96LB9	Antimicrobial activity	[173]
Pigment epithelium-derived factor	P36955	Protease inhibitor	[176]
Plasma kallikrein	P03952	Serine protease activity	[138]
Plasma serine protease inhibitor	P05154	Protease inhibitor	[177]
Plastin-2	P13796	Immunomodulatory effect	[178]
Poly(rC)-binding protein 1	Q15365	Antiviral effect	[179]
Poly(rC)-binding protein 2	Q15366	Antiviral effect	[180]
Pro-cathepsin H	P09668	Endopeptidase activity	[60]
Procathepsin L	P07711	Endopeptidase activity	[60]
Progranulin	P28799	Immunomodulatory effect	[184]
Prolactin-inducible protein	P12273	Aspartic-type endopeptidase activity<br>Modulates the activity of Zn-α2 glycoprotein	[185,186]
Proline-rich protein 27	Q6MZM9	Antimicrobial activity	[187]
Proline-rich protein 4	Q16378	Antimicrobial activity	[187]
Prolyl endopeptidase	P48147	Endopeptidase activity	[188]
Prosaposin	P07602	Processed forms has antimicrobial effect	[191]
Prostasin	Q16651	Serine protease activity	[192]
Protein AMBP	P02760	Protease inhibitor	[193]
Protein S100-A11	P31949	Immunomodulatory effect	[197]
Protein S100-A13	Q99584	Immunomodulatory effect	[198]
Protein S100-A4	P26447	Immunomodulatory effect	[196]
Protein S100-A6	P06703	Immunomodulatory effect	[201]
Protein S100-A7	P31151	Immunomodulatory effect	[196]
Protein S100-A8	P05109	Immunomodulatory effect	[196]
Protein S100-A9	P06702	Immunomodulatory effect	[196]
Protein S100-P	P25815	Immunomodulatory effect	[196]
Puromycin-sensitive aminopeptidase	P55786	Aminopeptidase activity	[203]
RelA-associated inhibitor	Q8WUF5	Antiviral effect<br>Immunomodulatory effect	[204]
Retroviral-like aspartic protease 1	Q53RT3	Aspartic-type endopeptidase activity	[205]
Ribonuclease 4	P34096	Ribonuclease activity	[170]
Ribonuclease T2	O00584	Ribonuclease activity	[170]
Secretoglobin family 1D member 1	O95968	Immunomodulatory effect	[207]
Secretoglobin family 1D member 2	O95969	Immunomodulatory effect	[207]
Serine protease 1	P07477	Serine protease activity	[212]
Serine protease HTRA1	Q92743	Serine protease activity	[212]
Serotransferrin	P02787	Iron sequestration	[214]

Serpin B3	P29508	Protease inhibitor	[215]
Serpin B5	P36952	Protease inhibitor	[215]
Serpin B6	P35237	Protease inhibitor	[215]
Serum amyloid P-component	P02743	Antiviral effect	[217]
Small proline-rich protein 3	Q9UBC9	Antimicrobial effect	[187]
Syntenin-1	O00560	Immunomodulatory effect	[219]
Thioredoxin domain-containing protein 17	Q9BRA2	Immunomodulatory effect	[221]
Thyroxine-binding globulin	P05543	Protease inhibitor	[223]
Toll-interacting protein	Q9H0E2	Immunomodulatory effect	[224]
Transgelin-2	P37802	Immunomodulatory effect	[226]
Triokinase/FMN cyclase	Q3LXA3	Immunomodulatory effect	[230]
Tripeptidyl-peptidase 1	O14773	Serine protease activity	[231]
Tripeptidyl-peptidase 2	P29144	Serine protease activity	[232]
Vitamin D-binding protein	P02774	Immunomodulatory effect	[239]
WAP four-disulfide core domain protein 2	Q14508	Protease inhibitor	[240]
Xaa-Pro aminopeptidase 1	Q9NQW7	Aminopeptidase activity	[241]
Xaa-Pro dipeptidase	P12955	Carboxypeptidase activity	[242]
Zinc-alpha-2-glycoprotein	P25311	Immunomodulatory effect	[243]
Zymogen granule membrane protein 16	O60844	Antimicrobial activity	[244]
Zymogen granule protein 16 homolog B	Q96DA0	Antimicrobial activity	[245]

Table S3. Proteins involved in the first line of host defense in saliva.

Protein name	UniProt entry	Function	Reference
Alpha-1-acid glycoprotein 1	P02763	Immunomodulatory effect	[3]
Alpha-1-acid glycoprotein 2	P19652	Immunomodulatory effect	[3]
Alpha-1-antichymotrypsin	P01011	Protease inhibitor	[4]
Alpha-1-antitrypsin	P01009	Protease inhibitor	[5]
Alpha-1B-glycoprotein	P04217	Immunomodulatory effect	[6]
Alpha-2-antiplasmin	P08697	Protease inhibitor	[7]
Alpha-2-HS-glycoprotein	P02765	Anti-inflammatory effect	[8]
Alpha-2-macroglobulin	P01023	Protease inhibitor	[9]
Alpha-2-macroglobulin-like protein 1	A8K2U0	Protease inhibitor	[10]
Alpha-amylase 1A	P0DUB6	Regulation of biofilm formation	[11]
Alpha-amylase 1B	P0DTE7	Regulation of biofilm formation	[11]
Alpha-amylase 1C	P0DTE8	Regulation of biofilm formation	[11]
Alpha-amylase 2B	P19961	Regulation of biofilm formation	[11]
Aminopeptidase B	Q9H4A4	Exopeptidase activity	[12]
Aminopeptidase N	P15144	Exopeptidase activity	[13]
Amyloid-beta precursor protein	P05067	Antimicrobial activity	[15]

Angiogenin	P03950	Antimicrobial activity	[16]
Antileukoproteinase	P03973	Protease inhibitor<br>Immunomodulatory effect	[17,18]
Antithrombin-III	P01008	Protease inhibitor	[19]
Apolipoprotein A-I	P02647	Antimicrobial activity	[20]
Apolipoprotein A-II	P02652	Immunomodulatory effect	[21]
Apolipoprotein A-IV	P06727	Immunomodulatory effect	[22]
Apolipoprotein B-100	P04114	Antimicrobial activity	[23]
Apolipoprotein C-III	P02656	Immunomodulatory effect	[24]
Apolipoprotein D	P05090	Immunomodulatory effect	[26]
Apolipoprotein E	P02649	Immunomodulatory effect	[27]
Apolipoprotein L1	O14791	Immunomodulatory effect	[28]
Apolipoprotein M	O95445	Immunomodulatory effect	[29]
Arginase-1	P05089	Antifungal activity<br>Immunomodulatory effect	[30,31]
Aspartyl aminopeptidase	Q9ULA0	Exopeptidase activity	[32]
Azurocidin	P20160	Antimicrobial activity	[33]
Bactericidal permeability-increasing protein	P17213	Antimicrobial activity	[33,34]
Beta-2-glycoprotein 1	P02749	Immunomodulatory effect	[35]
Beta-2-microglobulin	P61769	Antimicrobial activity<br>Immunomodulatory effect	[36,37]
Beta-Ala-His dipeptidase	Q96KN2	Carboxypeptidase activity	[38]
Beta-defensin 1	P60022	Antimicrobial activity	[33]
Beta-defensin 103	P81534	Antimicrobial activity	[33]
Beta-defensin 125	Q8N687	Antimicrobial activity	[33]
Beta-defensin 4A	O15263	Antimicrobial activity	[33]
Beta-hexosaminidase subunit alpha	P06865	Antimicrobial activity	[39]
Beta-hexosaminidase subunit beta	P07686	Antimicrobial activity	[39]
BPI fold-containing family A member 1	Q9NP55	Antimicrobial activity	[41]
BPI fold-containing family A member 2	Q96DR5	Antimicrobial activity	[42]
BPI fold-containing family B member 1	Q8TDL5	Antimicrobial activity	[44]
BPI fold-containing family B member 2	Q8N4F0	Antimicrobial activity	[44]
Brain-specific serine protease 4	Q9GZN4	Serine protease activity	[47]
Calcitonin gene-related peptide 1	P06881	Antimicrobial activity	[48]
Calpain-1 catalytic subunit	P07384	Endopeptidase activity<br>Immunomodulatory effect	[49]
Calpain-2 catalytic subunit	P17655	Endopeptidase activity<br>Immunomodulatory effect	[49]
Calpastatin	P20810	Protease inhibitor	[50]
Carboxypeptidase A4	Q9UI42	Carboxypeptidase activity	[51]
Carboxypeptidase B2	Q96IY4	Carboxypeptidase activity	[52]
Carboxypeptidase D	O75976	Carboxypeptidase activity	[53]
Carboxypeptidase E	P16870	Carboxypeptidase activity	[53]
Carboxypeptidase M	P14384	Carboxypeptidase activity	[54]
Carboxypeptidase N catalytic chain	P15169	Carboxypeptidase activity	[55]

Carboxypeptidase Q	Q9Y646	Carboxypeptidase activity	[56]
Carcinoembryonic antigen-related cell adhesion molecule 1	P13688	Immunomodulatory effect	[57]
Catalase	P04040	Antimicrobial activity	[59]
Cathelicidin antimicrobial peptide	P49913	Antimicrobial activity	[33]
Cathepsin B	P07858	Endopeptidase activity	[60]
Cathepsin D	P07339	Endopeptidase activity	[60]
Cathepsin F	Q9UBX1	Endopeptidase activity	[60]
Cathepsin G	P08311	Endopeptidase activity	[60]
Cathepsin L2	O60911	Endopeptidase activity	[60]
Cathepsin S	P25774	Endopeptidase activity	[60]
Cathepsin Z	Q9UBR2	Endopeptidase activity	[60]
Ceruloplasmin	P00450	Cu^{2+} sequestering activity	[62]
Chitinase-3-like protein 1	P36222	Antimicrobial activity	[63]
Chitotriosidase-1	Q13231	Antifungal activity	[64]
Chromogranin-A	P10645	Processed forms have anti-microbial activity	[65]
Clusterin	P10909	Immunomodulatory effect	[66]
Core histone macro-H2A.1	O75367	Antimicrobial activity	[68]
Corticosteroid-binding globulin	P08185	Protease inhibitor	[69]
Cystatin-A	P01040	Protease inhibitor	[71]
Cystatin-B	P04080	Protease inhibitor	[71]
Cystatin-C	P01034	Protease inhibitor	[71]
Cystatin-D	P28325	Protease inhibitor	[71]
Cystatin-F	O76096	Protease inhibitor	[71]
Cystatin-M	Q15828	Protease inhibitor	[71]
Cystatin-S	P01036	Protease inhibitor	[71]
Cystatin-SA	P09228	Protease inhibitor	[71]
Cystatin-SN	P01037	Protease inhibitor	[71]
Cytosol aminopeptidase	P28838	Aminopeptidase activity	[72]
Cytosolic non-specific dipeptidase	Q96KP4	Carboxypeptidase activity	[75]
Deleted in malignant brain tumors 1 protein	Q9UGM3	Immunomodulatory effect Antimicrobial activity	[76,77]
Deoxyribonuclease-1	P24855	Endonuclease activity	[78]
Dermcidin	P81605	Antimicrobial activity	[33]
Dipeptidyl peptidase 1	P53634	Carboxypeptidase activity	[81]
Dipeptidyl peptidase 2	Q9UHL4	Carboxypeptidase activity	[82]
Dipeptidyl peptidase 3	Q9NY33	Carboxypeptidase activity	[83]
Dipeptidyl peptidase 4	P27487	Carboxypeptidase activity	[84]
Disintegrin and metalloproteinase domain-containing protein 10	O14672	Metalloendopeptidase activity	[86]
Disintegrin and metalloproteinase domain-containing protein 15	Q13444	Metalloendopeptidase activity	[86]
Disintegrin and metalloproteinase	Q13443	Metalloendopeptidase	[86]

domain-containing protein 9		activity	
Drebrin-like protein	Q9UJU6	Immunomodulatory effect	[87]
Elafin	P19957	Protease inhibitor	[88]
Endoplasmic reticulum aminopeptidase 1	Q9NZ08	Aminopeptidase activity	[89]
Endoplasmic reticulum aminopeptidase 2	Q6P179	Aminopeptidase activity	[89]
Eosinophil cationic protein	P12724	Antimicrobial activity	[90]
Extracellular glycoprotein lacritin	Q9GZZ8	Antimicrobial activity	[92]
Fatty acid-binding protein 5	Q01469	Immunomodulatory effect	[94]
Fibrinogen alpha chain	P02671	Immunomodulatory effect	[95]
Fibrinogen beta chain	P02675	Immunomodulatory effect	[95]
Fibrinogen gamma chain	P02679	Immunomodulatory effect	[95]
Fibroleukin	Q14314	Immunomodulatory effect	[97]
Furin	P09958	Serine protease activity	[99]
Galectin-1	P09382	Immunomodulatory effect	[101]
Galectin-10	Q05315	Immunomodulatory effect	[102]
Galectin-3	P17931	Immunomodulatory effect	[103]
Galectin-3-binding protein	Q08380	Antimicrobial activity<br>Immunomodulatory effect	[104,105]
Galectin-7	P47929	Immunomodulatory effect	[106]
Gelsolin	P06396	Processed from has antimicrobial activity	[109]
Glucose-6-phosphate isomerase	P06744	Induces immunoglobulin secretion	[111]
Glutamate carboxypeptidase 2	Q04609	Carboxypeptidase activity	[112]
Glutathione S-transferase omega-1	P78417	Immunomodulatory effect	[114]
Glutathione S-transferase P	P09211	Immunomodulatory effect	[115]
Glyceraldehyde-3-phosphate dehydrogenase	P04406	Immunomodulatory effect	[116]
Growth-regulated alpha protein	P09341	Antimicrobial activity	[118]
Guanylate-binding protein 1	P32455	Immunomodulatory effect	[119]
Guanylate-binding protein 2	P32456	Antiviral effect	[120]
Guanylate-binding protein 4	Q96PP9	Immunomodulatory effect	[119]
Haptoglobin	P00738	Immunomodulatory effect<br>Iron sequestrating	[122]
Haptoglobin-related protein	P00739	Anti-parasitic effect	[123]
Heme-binding protein 1	Q9NRV9	Heme/iron sequestration	[124]
Heme-binding protein 2	Q9Y5Z4	Heme/iron sequestration	[124]
Hemoglobin subunit alpha	P69905	Processed forms (hemocidins) have antimicrobial activity	[125]
Hemoglobin subunit beta	P68871	Processed forms (hemocidins) have antimicrobial activity	[125]
Hemopexin	P02790	Antibacterail effect<br>Anti-inflammatory effect	[126]
Heparin cofactor 2	P05546	Protease inhibitor	[127]

High mobility group protein B1	P09429	Immunomodulatory effect	[130]
High mobility group protein B2	P26583	Antimicrobial activity	[131]
Histatin-1	P15515	Antimicrobial activity	[246]
Histatin-3	P15516	Antimicrobial activity	[246]
Histidine-rich glycoprotein	P04196	Antimicrobial activity	[133]
Histone H1.0	P07305	Antimicrobial activity	[134]
Histone H1.1	Q02539	Antimicrobial activity	[134]
Histone H1.10	Q92522	Antimicrobial activity	[134]
Histone H1.2	P16403	Antimicrobial activity	[134]
Histone H1.3	P16402	Antimicrobial activity	[134]
Histone H1.4	P10412	Antimicrobial activity	[134]
Histone H1.5	P16401	Antimicrobial activity	[134]
Histone H2A type 1	P0C0S8	Antimicrobial activity	[134]
Histone H2A type 1-B/E	P04908	Antimicrobial activity	[134]
Histone H2A type 1-D	P20671	Antimicrobial activity	[134]
Histone H2A type 1-H	Q96KK5	Antimicrobial activity	[134]
Histone H2A type 1-J	Q99878	Antimicrobial activity	[134]
Histone H2A type 2-A	Q6FI13	Antimicrobial activity	[134]
Histone H2A type 2-C	Q6FI13	Antimicrobial activity	[134]
Histone H2A type 3	Q7L7L0	Antimicrobial activity	[134]
Histone H2A.J	Q9BTM1	Antimicrobial activity	[134]
Histone H2A.Z	P0C0S5	Antimicrobial activity	[134]
Histone H2AX	P16104	Antimicrobial activity	[134]
Histone H2B type 1-A	Q96A08	Antimicrobial activity	[134]
Histone H2B type 1-B	P33778	Antimicrobial activity	[134]
Histone H2B type 1-C/E/F/G/I	P62807	Antimicrobial activity	[134]
Histone H2B type 1-D	P58876	Antimicrobial activity	[134]
Histone H2B type 1-H	Q93079	Antimicrobial activity	[134]
Histone H2B type 1-J	P06899	Antimicrobial activity	[134]
Histone H2B type 1-K	O60814	Antimicrobial activity	[134]
Histone H2B type 1-L	Q99880	Antimicrobial activity	[134]
Histone H2B type 1-M	Q99879	Antimicrobial activity	[134]
Histone H2B type 1-N	Q99877	Antimicrobial activity	[134]
Histone H2B type 1-O	P23527	Antimicrobial activity	[134]
Histone H2B type 2-E	Q16778	Antimicrobial activity	[134]
Histone H2B type 2-F	Q5QNW6	Antimicrobial activity	[134]
Histone H2B type 3-B	Q8N257	Antimicrobial activity	[134]
Histone H2B type F-S	P57053	Antimicrobial activity	[134]
Histone H3.1	P68431	Antimicrobial activity	[134]
Histone H3.1t	Q16695	Antimicrobial activity	[134]
Histone H3.2	Q71DI3	Antimicrobial activity	[134]
Histone H3.3	P84243	Antimicrobial activity	[134]
Histone H3.3C	Q6NXT2	Antimicrobial activity	[134]

Histone H4	P62805	Antimicrobial activity	[134]
Inter-alpha-trypsin inhibitor heavy chain H1	P19827	Protease inhibitor	[135]
Inter-alpha-trypsin inhibitor heavy chain H2	P19823	Protease inhibitor	[135]
Inter-alpha-trypsin inhibitor heavy chain H3	Q06033	Protease inhibitor	[135]
Inter-alpha-trypsin inhibitor heavy chain H4	Q14624	Protease inhibitor	[135]
Kallikrein-10	O43240	Serine protease activity	[138]
Kallikrein-11	Q9UBX7	Serine protease activity	[138]
Kallikrein-12	Q9UKR0	Serine protease activity	[138]
Kallikrein-13	Q9UKR3	Serine protease activity	[138]
Kallikrein-14	Q9P0G3	Serine protease activity	[138]
Kallikrein-6	Q92876	Serine protease activity	[138]
Kallikrein-7	P49862	Serine protease activity<br>Processing the maturation of LL-37 cathelicidin	[138,139]
Kallikrein-8	O60259	Serine protease activity	[138]
Kininogen-1	P01042	Antimicrobial activity	[140,141]
Kunitz-type protease inhibitor 1	O43278	Protease inhibitor	[142]
Kunitz-type protease inhibitor 2	O43291	Protease inhibitor	[142]
Lactoperoxidase	P22079	Antimicrobial activity	[143]
Lactotransferrin	P02788	Antimicrobial activity<br>Iron sequestration	[33]
Legumain	Q99538	Endopeptidase activity	[144]
Leukocyte elastase inhibitor	P30740	Protease inhibitor	[145]
Lipocalin-1	P31025	Immunomodulatory effect<br>Iron sequestration	[146,147]
Lipocalin-2	P80188	Immunomodulatory effect<br>Iron sequestration	[146,147]
Lipopolysaccharide-binding protein	P18428	Immunomodulatory effect	[148]
Lymphotactin	P47992	Antimicrobial activity	[150]
Lysozyme C	P61626	Antimicrobial activity	[33]
Macrophage migration inhibitory factor	P14174	Antimicrobial activity	[151]
Major vault protein	Q14764	Immunomodulatory effect	[152]
Mammaglobin-B	O75556	Immunomodulatory effect	[153]
Matrix metalloproteinase-9	P14780	Metalloprotease activity	[155]
Melanotransferrin	P08582	Iron sequestration	[156]
Metalloproteinase inhibitor 1	P01033	Protease Inhibitor	[157]
Metalloproteinase inhibitor 2	P16035	Protease Inhibitor	[157]
Midkine	P21741	Immunomodulatory effect	[158]
Moesin	P26038	Immunomodulatory effect	[159]
Mucin-1	P15941	Antimicrobial activity	[160]
Mucin-13	Q9H3R2	Antimicrobial activity	[160]
Mucin-15	Q8N387	Antimicrobial activity	[160]
Mucin-16	Q8WXI7	Antimicrobial activity	[160]
Mucin-4	Q99102	Antimicrobial activity	[160]

Mucin-5AC	P98088	Antimicrobial activity	[160]
Mucin-5B	Q9HC84	Antimicrobial activity	[160]
Mucin-6	Q6W4X9	Antimicrobial activity	[160]
Mucin-7	Q8TAX7	Antimicrobial activity	[160]
Myeloblastin	P24158	Serine protease activity	[161]
Myeloperoxidase	P05164	Antimicrobial activity	[162]
N-acetylmuramoyl-L-alanine amidase	Q96PD5	Antimicrobial activity	[164]
Neutrophil collagenase	P22894	Endopeptidase activity Immunomodulatory effect	[166]
Neutrophil defensin 1	P59665	Antimicrobial activity	[33]
Neutrophil defensin 3	P59666	Antimicrobial activity	[33]
Neutrophil defensin 4	P12838	Antimicrobial activity	[33]
Neutrophil elastase	P08246	Serine protease activity	[167]
Nicotinamide phosphoribosyltransferase	P43490	Immunomodulatory effect	[168]
Non-histone chromosomal protein HMG-17	P05204	Antimicrobial activity	[169]
Non-secretory ribonuclease	P10153	Ribonuclease activity	[170]
Opiorphin prepropeptide	Q99935	Protease inhibitor	[171]
Peptidase inhibitor 16	Q6UXB8	Protease inhibitor	[172]
Peptidoglycan recognition protein 1	O75594	Antimicrobial activity	[173]
Peptidoglycan recognition protein 3	Q96LB9	Antimicrobial activity	[173]
Phospholipase B-like 1	Q6P4A8	Suggested antimicrobial activity	[175]
Pigment epithelium-derived factor	P36955	Protease inhibitor	[176]
Plasma kallikrein	P03952	Serine protease activity	[138]
Plasma serine protease inhibitor	P05154	Protease inhibitor	[177]
Plastin-2	P13796	Immunomodulatory effect	[178]
Poly(rC)-binding protein 1	Q15365	Antiviral effect	[179]
Poly(rC)-binding protein 2	Q15366	Antiviral effect	[180]
Pregnancy zone protein	P20742	Protease inhibitor	[181]
Pro-cathepsin H	P09668	Endopeptidase activity	[60]
Procathepsin L	P07711	Endopeptidase activity	[60]
Progranulin	P28799	Immunomodulatory effect	[184]
Prolactin-inducible protein	P12273	Aspartic-type endopeptidase activity Modulates the activity of Zn-α2 glycoprotein	[185,186]
Proline-rich protein 27	Q16378	Antimicrobial activity	[187]
Proline-rich protein 4	Q16378	Antimicrobial activity	[187]
Prolyl endopeptidase	P48147	Endopeptidase activity	[188]
Prosaposin	P07602	Processed forms has antimicrobial effect	[191]
Prostasin	Q16651	Serine protease activity	[192]
Protein AMBP	P02760	Protease inhibitor	[193]
Protein S100-A10	P60903	Immunomodulatory effect	[196]
Protein S100-A11	P31949	Immunomodulatory effect	[197]

Protein S100-A12	P80511	Immunomodulatory effect	[196]
Protein S100-A13	Q99584	Immunomodulatory effect	[198]
Protein S100-A14	Q9HCY8	Immunomodulatory effect	[199]
Protein S100-A2	P29034	Immunomodulatory effect	[200]
Protein S100-A4	P26447	Immunomodulatory effect	[196]
Protein S100-A6	P06703	Immunomodulatory effect	[201]
Protein S100-A7	P31151	Immunomodulatory effect	[196]
Protein S100-A8	P05109	Immunomodulatory effect	[196]
Protein S100-A9	P06702	Immunomodulatory effect	[196]
Protein S100-P	P25815	Immunomodulatory effect	[196]
Puromycin-sensitive aminopeptidase	P55786	Aminopeptidase activity	[203]
Ribonuclease 4	P34096	Ribonuclease activity	[170]
Ribonuclease 7	Q9H1E1	Ribonuclease activity	[170]
Ribonuclease pancreatic	P07998	Ribonuclease activity	[170]
Ribonuclease T2	O00584	Ribonuclease activity	[170]
Secreted Ly-6/uPAR domain-containing protein 2	P0DP57	Immunomodulatory effect	[247]
Secreted Ly-6/uPAR-related protein 1	P55000	Immunomodulatory effect	[206]
Secretoglobin family 1D member 1	O95968	Immunomodulatory effect	[207]
Secretoglobin family 1D member 2	O95969	Immunomodulatory effect	[207]
Secretoglobin family 3A member 1	Q96QR1	Immunomodulatory effect	[208]
Semenogelin-1	P04279	Processed forms has antimicrobial activity	[209–211]
Semenogelin-2	Q02383	Processed forms has antimicrobial activity	[209–211]
Serine protease 1	P07477	Serine protease activity	[212]
Serine protease 23	O95084	Serine protease activity	[212]
Serine protease 27	Q9BQR3	Serine protease activity	[212]
Serine protease HTRA1	Q92743	Serine protease activity	[212]
Serine protease inhibitor Kazal-type 5	Q9NQ38	Protease inhibitor	[213]
Serine protease inhibitor Kazal-type 7	P58062	Protease inhibitor	[213]
Serotransferrin	P02787	Iron sequestration	[214]
Serpin B10	P48595	Protease inhibitor	[215]
Serpin B12	Q96P63	Protease inhibitor	[215]
Serpin B13	Q9UIV8	Protease inhibitor	[215]
Serpin B3	P29508	Protease inhibitor	[215]
Serpin B4	P48594	Protease inhibitor	[215]
Serpin B5	P36952	Protease inhibitor	[215]
Serpin B6	P35237	Protease inhibitor	[215]
Serpin B8	P50452	Protease inhibitor	[215]
Serpin B9	P50453	Protease inhibitor	[215]
Serum amyloid A-1 protein	P0DJI8	Immunomodulatory effect	[216]
Serum amyloid A-2 protein	P0DJI9	Immunomodulatory effect	[216]
Serum amyloid A-4 protein	P35542	Immunomodulatory effect	[216]

Serum amyloid P-component	P02743	Antiviral effect	[217]
Small proline-rich protein 3	Q9UBC9	Antimicrobial effect	[187]
Syntenin-1	O00560	Immunomodulatory effect	[219]
T-cell immunomodulatory protein	Q8TB96	Immunomodulatory effect	[220]
Thioredoxin domain-containing protein 17	Q9BRA2	Immunomodulatory effect	[221]
Thymosin beta-10	P63313	Antimicrobial activity	[222]
Thymosin beta-4	P62328	Antimicrobial activity	[222]
Thyroxine-binding globulin	P05543	Protease inhibitor	[223]
Toll-interacting protein	Q9H0E2	Immunomodulatory effect	[224]
Transgelin-2	P37802	Immunomodulatory effect	[226]
Transmembrane protease serine 11A	Q6ZMR5	Serine protease activity	[227]
Transmembrane protease serine 11B	Q86T26	Serine protease activity	[248]
Transmembrane protease serine 11D	O60235	Serine protease activity	[228]
Transmembrane protease serine 11E	Q9UL52	Serine protease activity	[229]
Triokinase/FMN cyclase	Q3LXA3	Immunomodulatory effect	[230]
Tripeptidyl-peptidase 1	O14773	Serine protease activity	[231]
Tripeptidyl-peptidase 2	P29144	Serine protease activity	[232]
Trypsin-3	P35030	Serine protease activity	[234]
Uromodulin	P07911	Antimicrobial activity	[236,237]
Uteroglobin	P11684	Immunomodulatory effect	[238]
Vitamin D-binding protein	P02774	Immunomodulatory effect	[239]
WAP four-disulfide core domain protein 12	Q8WWY7	Protease inhibitor	[240]
WAP four-disulfide core domain protein 2	Q14508	Protease inhibitor	[240]
Xaa-Pro aminopeptidase 1	Q9NQW7	Aminopeptidase activity	[241]
Xaa-Pro dipeptidase	P12955	Carboxypeptidase activity	[242]
Zinc-alpha-2-glycoprotein	P25311	Immunomodulatory effect	[243]
Zymogen granule membrane protein 16	O60844	Antimicrobial activity	[244]
Zymogen granule protein 16 homolog B	Q96DA0	Antimicrobial activity	[245]

Table S4. Proteins involved in the first line of host defense in sweat.

Protein name	UniProt entry	Function	Reference
Alpha-1-acid glycoprotein 1	P02763	Immunomodulatory effect	[3]
Alpha-1-acid glycoprotein 2	P19652	Immunomodulatory effect	[3]
Alpha-2-HS-glycoprotein	P02765	Anti-inflammatory effect	[8]
Alpha-2-macroglobulin	P01023	Protease inhibitor	[9]
Alpha-2-macroglobulin-like protein 1	A8K2U0	Protease inhibitor	[10]
Aminopeptidase N	P15144	Exopeptidase activity	[13]
Angiogenin	P03950	Antimicrobial activity	[16]
Antileukoproteinase	P03973	Protease inhibitor<br>Immunomodulatory effect	[17,18]
Antithrombin-III	P01008	Protease inhibitor	[19]
Apolipoprotein A-I	P02647	Antimicrobial activity	[20]
Apolipoprotein D	P05090	Immunomodulatory effect	[26]

Beta-2-glycoprotein 1	P02749	Immunomodulatory effect	[35]
Beta-2-microglobulin	P61769	Antimicrobial activity<br>Immunomodulatory effect	[36,37]
Beta-hexosaminidase subunit alpha	P06865	Antimicrobial activity	[39]
Beta-hexosaminidase subunit beta	P07686	Antimicrobial activity	[39]
BPI fold-containing family A member 1	Q9NP55	Antimicrobial activity	[41]
BPI fold-containing family A member 2	Q96DR5	Antimicrobial activity	[42]
BPI fold-containing family B member 4	P59827	Antimicrobial activity	[46]
Calpain-1 catalytic subunit	P07384	Endopeptidase activity<br>Immunomodulatory effect	[49]
Carboxypeptidase A2	P48052	Carboxypeptidase activity	[51]
Carboxypeptidase A4	Q9UI42	Carboxypeptidase activity	[51]
Carboxypeptidase M	P14384	Carboxypeptidase activity	[54]
Carboxypeptidase Q	Q9Y646	Carboxypeptidase activity	[56]
Catalase	P04040	Antimicrobial activity	[59]
Cathelicidin antimicrobial peptide	P49913	Antimicrobial activity	[33]
Cathepsin B	P07858	Endopeptidase activity	[60]
Cathepsin D	P07339	Endopeptidase activity	[60]
Cathepsin F	Q9UBX1	Endopeptidase activity	[60]
Cathepsin L2	O60911	Endopeptidase activity	[60]
Cathepsin Z	Q9UBR2	Endopeptidase activity	[60]
Chitinase-3-like protein 1	P36222	Antimicrobial activity	[63]
Clusterin	P10909	Immunomodulatory effect	[66]
Cystatin-A	P01040	Protease inhibitor	[71]
Cystatin-B	P04080	Protease inhibitor	[71]
Cystatin-C	P01034	Protease inhibitor	[71]
Cystatin-D	P28325	Protease inhibitor	[71]
Cystatin-M	Q15828	Protease inhibitor	[71]
Cystatin-S	P01036	Protease inhibitor	[71]
Cystatin-SA	P09228	Protease inhibitor	[71]
Cystatin-SN	P01037	Protease inhibitor	[71]
Deoxyribonuclease-1	P24855	Endonuclease activity	[78]
Dermcidin	P81605	Antimicrobial activity	[33]
Dipeptidyl peptidase 1	P53634	Carboxypeptidase activity	[81]
Dipeptidyl peptidase 2	Q9UHL4	Carboxypeptidase activity	[82]
Dipeptidyl peptidase 3	Q9NY33	Carboxypeptidase activity	[83]
Dipeptidyl peptidase 4	P27487	Carboxypeptidase activity	[84]
Disintegrin and metalloproteinase domain-containing protein 10	O14672	Metalloendopeptidase activity	[86]
Elafin	P19957	Protease inhibitor	[88]
Extracellular glycoprotein lacritin	Q9GZZ8	Antimicrobial activity	[92]
Fatty acid-binding protein 5	Q01469	Immunomodulatory effect	[94]
Fibrinogen alpha chain	P02671	Immunomodulatory effect	[95]
Fibrinogen beta chain	P02675	Immunomodulatory effect	[95]

Fibrinogen gamma chain	P02679	Immunomodulatory effect	[95]
Furin	P09958	Serine protease activity	[99]
Galectin-3	P17931	Immunomodulatory effect	[103]
Galectin-3-binding protein	Q08380	Antimicrobial activity<br>Immunomodulatory effect	[104,105]
Galectin-7	P47929	Immunomodulatory effect	[106]
Gelsolin	P06396	Processed from has antimicrobial activity	[109]
Glutathione S-transferase P	P09211	Immunomodulatory effect	[115]
Glyceraldehyde-3-phosphate dehydrogenase	P04406	Immunomodulatory effect	[116]
Guanylate-binding protein 1	P32455	Immunomodulatory effect	[119]
Hemoglobin subunit alpha	P69905	Processed forms (hemocidins) have antimicrobial activity	[125]
Hemoglobin subunit beta	P68871	Processed forms (hemocidins) have antimicrobial activity	[125]
Hemopexin	P02790	Antibacterail effect<br>Anti-inflammatory effect	[126]
Histatin-1	P15515	Antimicrobial activity	[246]
Histidine-rich glycoprotein	P04196	Antimicrobial activity	[133]
Histone H2A.J	Q9BTM1	Antimicrobial activity	[134]
Histone H2B type 3-B	Q8N257	Antimicrobial activity	[134]
Histone H4	P62805	Antimicrobial activity	[134]
Kallikrein-10	O43240	Serine protease activity	[138]
Kallikrein-13	Q9UKR3	Serine protease activity	[138]
Kallikrein-5	Q9Y337	Serine protease activity<br>Processing the maturation of LL-37 cathelicidin	[138,139]
Kallikrein-9	Q9UKQ9	Serine protease activity	[138]
Lactotransferrin	P02788	Antimicrobial activity<br>Iron sequestration	[33]
Legumain	Q99538	Endopeptidase activity	[144]
Lipocalin-1	P31025	Immunomodulatory effect<br>Iron sequestration	[146,147]
Lipocalin-2	P80188	Immunomodulatory effect<br>Iron sequestration	[146,147]
Lysozyme C	P61626	Antimicrobial activity	[33]
Major vault protein	Q14764	Immunomodulatory effect	[152]
Mammaglobin-B	O75556	Immunomodulatory effect	[153]
Matrix metalloproteinase-9	P14780	Metalloprotease activity	[155]
Metalloproteinase inhibitor 2	P16035	Protease Inhibitor	[157]
Moesin	P26038	Immunomodulatory effect	[159]
Mucin-5AC	P98088	Antimicrobial activity	[160]
Mucin-5B	Q9HC84	Antimicrobial activity	[160]

Mucin-7	Q8TAX7	Antimicrobial activity	[160]
Myeloblastin	P24158	Serine protease activity	[161]
Neutrophil collagenase	P22894	Endopeptidase activity Immunomodulatory effect	[166]
Neutrophil defensin 1	P59665	Antimicrobial activity	[33]
Non-secretory ribonuclease	P10153	Ribonuclease activity	[170]
Opiorphin prepropeptide	Q99935	Protease inhibitor	[171]
Peptidoglycan recognition protein 3	Q96LB9	Antimicrobial activity	[173]
Phospholipase B-like 1	Q6P4A8	Suggested antimicrobial activity	[175]
Pigment epithelium-derived factor	P36955	Protease inhibitor	[176]
Plastin-2	P13796	Immunomodulatory effect	[178]
Pro-cathepsin H	P09668	Endopeptidase activity	[60]
Procathepsin L	P07711	Endopeptidase activity	[60]
Prolactin-inducible protein	P12273	Aspartic-type endopeptidase activity Modulates the activity of Zn-α2 glycoprotein	[185,186]
Proline-rich protein 27	Q16378	Antimicrobial activity	[187]
Proline-rich protein 4	Q16378	Antimicrobial activity	[187]
Prolyl endopeptidase	P48147	Endopeptidase activity	[188]
Protein AMBP	P02760	Protease inhibitor	[193]
Protein S100-A10	P60903	Immunomodulatory effect	[196]
Protein S100-A11	P31949	Immunomodulatory effect	[197]
Protein S100-A12	P80511	Immunomodulatory effect	[196]
Protein S100-A2	P29034	Immunomodulatory effect	[200]
Protein S100-A7	P31151	Immunomodulatory effect	[196]
Protein S100-A8	P05109	Immunomodulatory effect	[196]
Protein S100-A9	P06702	Immunomodulatory effect	[196]
Protein S100-P	P25815	Immunomodulatory effect	[196]
Puromycin-sensitive aminopeptidase	P55786	Aminopeptidase activity	[203]
Ribonuclease 4	P34096	Ribonuclease activity	[170]
Ribonuclease 7	Q9H1E1	Ribonuclease activity	[170]
Secretoglobin family 1D member 1	O95968	Immunomodulatory effect	[207]
Secretoglobin family 1D member 2	O95969	Immunomodulatory effect	[207]
Serine protease inhibitor Kazal-type 7	P58062	Protease inhibitor	[213]
Serine protease inhibitor Kazal-type 9	Q5DT21	Protease inhibitor	[213]
Serotransferrin	P02787	Iron sequestration	[214]
Serpin B12	Q96P63	Protease inhibitor	[215]
Serpin B5	P36952	Protease inhibitor	[215]
Small proline-rich protein 4	Q96PI1	Antimicrobial effect	[187]
Thioredoxin domain-containing protein 17	Q9BRA2	Immunomodulatory effect	[221]
Thymosin beta-4	P62328	Antimicrobial activity	[222]
Toll-interacting protein	Q9H0E2	Immunomodulatory effect	[224]
WAP four-disulfide core domain protein 12	Q8WWY7	Protease inhibitor	[240]

WAP four-disulfide core domain protein 3	Q8IUB2	Protease inhibitor	[240]
Zinc-alpha-2-glycoprotein	P25311	Immunomodulatory effect	[243]
Zymogen granule protein 16 homolog B	Q96DA0	Antimicrobial activity	[245]

Table S5. Proteins involved in the first line of host defense in the nasal secretion.

Protein name	UniProt entry	Function	Reference
Alpha-1-acid glycoprotein 1	P02763	Immunomodulatory effect	[3]
Alpha-1-acid glycoprotein 2	P19652	Immunomodulatory effect	[3]
Alpha-1-antichymotrypsin	P01011	Protease inhibitor	[4]
Alpha-1-antitrypsin	P01009	Protease inhibitor	[5]
Alpha-1B-glycoprotein	P04217	Immunomodulatory effect	[6]
Alpha-2-HS-glycoprotein	P02765	Anti-inflammatory effect	[8]
Alpha-2-macroglobulin	P01023	Protease inhibitor	[9]
Alpha-amylase 1A	P0DUB6	Regulation of biofilm formation	[11]
Alpha-amylase 1B	P0DTE7	Regulation of biofilm formation	[11]
Alpha-amylase 1C	P0DTE8	Regulation of biofilm formation	[11]
Amyloid-beta precursor protein	P05067	Antimicrobial activity	[15]
Angiogenin	P03950	Antimicrobial activity	[16]
Antileukoproteinase	P03973	Protease inhibitor<br>Immunomodulatory effect	[17,18]
Antithrombin-III	P01008	Protease inhibitor	[19]
Apolipoprotein A-I	P02647	Antimicrobial activity	[20]
Apolipoprotein A-II	P02652	Immunomodulatory effect	[21]
Apolipoprotein A-IV	P06727	Immunomodulatory effect	[22]
Apolipoprotein B-100	P04114	Antimicrobial activity	[23]
Apolipoprotein C-III	P02656	Immunomodulatory effect	[24]
Apolipoprotein D	P05090	Immunomodulatory effect	[26]
Apolipoprotein E	P02649	Immunomodulatory effect	[27]
Azurocidin	P20160	Antimicrobial activity	[33]
Bactericidal permeability-increasing protein	P17213	Antimicrobial activity	[33,34]
Beta-2-glycoprotein 1	P02749	Immunomodulatory effect	[35]
Beta-2-microglobulin	P61769	Antimicrobial activity<br>Immunomodulatory effect	[36,37]
Beta-hexosaminidase subunit beta	P07686	Antimicrobial activity	[39]
BPI fold-containing family A member 1	Q9NP55	Antimicrobial activity	[41]
BPI fold-containing family B member 1	Q8TDL5	Antimicrobial activity	[44]
BPI fold-containing family B member 2	Q8N4F0	Antimicrobial activity	[44]
BPI fold-containing family B member 3	P59826	Antimicrobial activity	[45]
BPI fold-containing family B member 4	P59827	Antimicrobial activity	[46]
Calcitonin gene-related peptide 1	P06881	Antimicrobial activity	[48]
Calpain-1 catalytic subunit	P07384	Endopeptidase activity	[49]

Catalase	P04040	Immunomodulatory effect<br>Antimicrobial activity	[59]
Cathelicidin antimicrobial peptide	P49913	Antimicrobial activity	[33]
Cathepsin B	P07858	Endopeptidase activity	[60]
Cathepsin D	P07339	Endopeptidase activity	[60]
Cathepsin G	P08311	Endopeptidase activity	[60]
Cathepsin S	P25774	Endopeptidase activity	[60]
Ceruloplasmin	P00450	Cu^{2+} sequestration	[62]
Clusterin	P10909	Immunomodulatory effect	[66]
Corticosteroid-binding globulin	P08185	Protease inhibitor	[69]
Cystatin-B	P04080	Protease inhibitor	[71]
Cystatin-C	P01034	Protease inhibitor	[71]
Cystatin-D	P28325	Protease inhibitor	[71]
Cystatin-S	P01036	Protease inhibitor	[71]
Cystatin-SA	P09228	Protease inhibitor	[71]
Cystatin-SN	P01037	Protease inhibitor	[71]
Cytosol aminopeptidase	P28838	Aminopeptidase activity	[72]
Cytosolic non-specific dipeptidase	Q96KP4	Carboxypeptidase activity	[75]
Deleted in malignant brain tumors 1 protein	Q9UGM3	Immunomodulatory effect<br>Antimicrobial activity	[76,77]
Dermcidin	P81605	Antimicrobial activity	[33]
Elafin	P19957	Protease inhibitor	[88]
Eosinophil cationic protein	P12724	Antimicrobial activity	[90]
Eosinophil peroxidase	P11678	Antimicrobial activity	[91]
Extracellular glycoprotein lacritin	Q9GZZ8	Antimicrobial activity	[92]
Fatty acid-binding protein 5	Q01469	Immunomodulatory effect	[94]
Fibrinogen alpha chain	P02671	Immunomodulatory effect	[95]
Fibrinogen beta chain	P02675	Immunomodulatory effect	[95]
Fibrinogen gamma chain	P02679	Immunomodulatory effect	[95]
Galectin-10	Q05315	Immunomodulatory effect	[102]
Galectin-3	P17931	Immunomodulatory effect	[103]
Galectin-3-binding protein	Q08380	Antimicrobial activity<br>Immunomodulatory effect	[104,105]
Gelsolin	P06396	Processed from has antimicrobial activity	[109]
Glucose-6-phosphate isomerase	P06744	Induces immunoglobulin secretion	[111]
Glutathione S-transferase P	P09211	Immunomodulatory effect	[115]
Glyceraldehyde-3-phosphate dehydrogenase	P04406	Immunomodulatory effect	[116]
Haptoglobin	P00738	Immunomodulatory effect<br>Iron sequestration	[122]
Haptoglobin-related protein	P00739	Antiparasitic effect	[123]
Hemoglobin subunit alpha	P69905	Processed forms (hemocidins) have antimicrobial activity	[125]

Hemoglobin subunit beta	P68871	Processed forms (hemo-cidins) have antimicrobial activity	[125]
Hemopexin	P02790	Antibacterail effect<br>Anti-inflammatory effect	[126]
Heparin cofactor 2	P05546	Protease inhibitor	[127]
High mobility group protein B1	P09429	Immunomodulatory effect	[130]
High mobility group protein B2	P26583	Antimicrobial activity	[131]
Histidine-rich glycoprotein	P04196	Antimicrobial activity	[133]
Histone H1.0	P07305	Antimicrobial activity	[134]
Histone H1.3	P16402	Antimicrobial activity	[134]
Histone H1.4	P10412	Antimicrobial activity	[134]
Histone H2A type 1-D	P20671	Antimicrobial activity	[134]
Histone H2A type 1-H	Q96KK5	Antimicrobial activity	[134]
Histone H2A type 2-C	Q16777	Antimicrobial activity	[134]
Histone H2A.Z	P0C0S5	Antimicrobial activity	[134]
Histone H2B type 1-C/E/F/G/I	P62807	Antimicrobial activity	[134]
Histone H2B type 1-D	P58876	Antimicrobial activity	[134]
Histone H2B type 1-H	Q93079	Antimicrobial activity	[134]
Histone H2B type 1-J	P06899	Antimicrobial activity	[134]
Histone H2B type 1-K	O60814	Antimicrobial activity	[134]
Histone H2B type 1-L	Q99880	Antimicrobial activity	[134]
Histone H2B type 1-M	Q99879	Antimicrobial activity	[134]
Histone H2B type 1-N	Q99877	Antimicrobial activity	[134]
Histone H2B type 2-F	Q5QNW6	Antimicrobial activity	[134]
Histone H2B type F-S	P57053	Antimicrobial activity	[134]
Histone H3.1	P68431	Antimicrobial activity	[134]
Histone H3.1t	Q16695	Antimicrobial activity	[134]
Histone H3.2	Q71DI3	Antimicrobial activity	[134]
Histone H3.3	P84243	Antimicrobial activity	[134]
Histone H4	P62805	Antimicrobial activity	[134]
Inter-alpha-trypsin inhibitor heavy chain H1	P19827	Protease inhibitor	[135]
Inter-alpha-trypsin inhibitor heavy chain H2	P19823	Protease inhibitor	[135]
Inter-alpha-trypsin inhibitor heavy chain H4	Q14624	Protease inhibitor	[135]
Kallikrein-7	P49862	Serine protease activity<br>Processing of LL37 cathelicidn	[138]
Kininogen-1	P01042	Antimicrobial activity	[140,141]
Lactoperoxidase	P22079	Antimicrobial activity	[143]
Lactotransferrin	P02788	Antimicrobial activity<br>Iron sequestration	[33]
Leukocyte elastase inhibitor	P30740	Protease inhibitor	[145]
Lipocalin-1	P31025	Immunomodulatory effect<br>Iron sequestration	[146,147]
Lipocalin-2	P80188	Immunomodulatory effect	[146,147]

		Iron sequestration	
Lysozyme C	P61626	Antimicrobial activity	[33]
Macrophage migration inhibitory factor	P14174	Antimicrobial activity	[151]
Mammaglobin-B	O75556	Immunomodulatory effect	[153]
Matrix metalloproteinase-9	P14780	Metalloprotease activity	[155]
Metalloproteinase inhibitor 1	P01033	Protease Inhibitor	[157]
Moesin	P26038	Immunomodulatory effect	[159]
Mucin-5AC	P98088	Antimicrobial activity	[160]
Mucin-5B	Q9HC84	Antimicrobial activity	[160]
Mucin-7	Q8TAX7	Antimicrobial activity	[160]
Myeloblastin	P24158	Serine protease activity	[161]
Myeloperoxidase	P05164	Antimicrobial activity	[162]
N-acetylmuramoyl-L-alanine amidase	Q96PD5	Antimicrobial activity	[164]
Neutrophil collagenase	P22894	Endopeptidase activity<br>Immunomodulatory effect	[166]
Neutrophil defensin 1	P59665	Antimicrobial activity	[33]
Neutrophil defensin 3	P59666	Antimicrobial activity	[33]
Neutrophil elastase	P08246	Serine protease activity	[167]
Nicotinamide phosphoribosyltransferase	P43490	Immunomodulatory effect	[168]
Non-secretory ribonuclease	P10153	Ribonuclease activity	[170]
Opiorphin prepropeptide	Q99935	Protease inhibitor	[171]
Peptidoglycan recognition protein 1	O75594	Antimicrobial activity	[173]
Pigment epithelium-derived factor	P36955	Protease inhibitor	[176]
Plasma kallikrein	P03952	Serine protease activity	[138]
Plastin-2	P13796	Immunomodulatory effect	[178]
Progranulin	P28799	Immunomodulatory effect	[184]
Prolactin-inducible protein	P12273	Aspartic-type endopeptidase activity<br>Modulates the activity of Zn-α2 glycoprotein	[185,186]
Proline-rich protein 4	Q16378	Antimicrobial activity	[187]
Prosaposin	P07602	Processed forms have antimicrobial effect	[191]
Protein AMBP	P02760	Protease inhibitor	[193]
Protein S100-A11	P31949	Immunomodulatory effect	[197]
Protein S100-A12	P80511	Immunomodulatory effect	[196]
Protein S100-A4	P26447	Immunomodulatory effect	[196]
Protein S100-A6	P06703	Immunomodulatory effect	[201]
Protein S100-A7	P31151	Immunomodulatory effect	[196]
Protein S100-A8	P05109	Immunomodulatory effect	[196]
Protein S100-A9	P06702	Immunomodulatory effect	[196]
Protein S100-P	P25815	Immunomodulatory effect	[196]
Ribonuclease pancreatic	P07998	Ribonuclease activity	[170]
Secretoglobin family 1C member 1	Q8TD33	Immunomodulatory effect	[249]
Secretoglobin family 1D member 1	O95968	Immunomodulatory effect	[207]

Secretoglobin family 1D member 2	O95969	Immunomodulatory effect	[207]
Semenogelin-1	P04279	Processed forms have antimicrobial effect	[209–211]
Semenogelin-2	Q02383	Processed forms have antimicrobial effect	[209–211]
Serotransferrin	P02787	Iron sequestration	[214]
Serpin B3	P29508	Protease inhibitor	[215]
Serpin B4	P48594	Protease inhibitor	[215]
Serum amyloid P-component	P02743	Antiviral effect	[217]
Small proline-rich protein 3	Q9UBC9	Antimicrobial effect	[187]
Thymosin beta-10	P63313	Antimicrobial activity	[222]
Thymosin beta-4	P62328	Antimicrobial activity	[222]
Thyroxine-binding globulin	P05543	Protease inhibitor	[223]
Transgelin-2	P37802	Immunomodulatory effect	[226]
Uteroglobin	P11684	Immunomodulatory effect	[238]
Vitamin D-binding protein	P02774	Immunomodulatory effect	[239]
WAP four-disulfide core domain protein 2	Q14508	Protease inhibitor	[240]
Zinc-alpha-2-glycoprotein	P25311	Immunomodulatory effect	[243]
Zymogen granule membrane protein 16	O60844	Antimicrobial activity	[244]

Table S6. Proteins involved in the first line of host defense in urine.

Protein name	UniProt entry	Function	Reference
ADAM DEC1	O15204	Immunomodulatory effect	[2]
Alpha-1-acid glycoprotein 1	P02763	Immunomodulatory effect	[3]
Alpha-1-acid glycoprotein 2	P19652	Immunomodulatory effect	[3]
Alpha-1-antichymotrypsin	P01011	Protease inhibitor	[4]
Alpha-1-antitrypsin	P01009	Protease inhibitor	[5]
Alpha-1B-glycoprotein	P04217	Immunomodulatory effect	[6]
Alpha-2-antiplasmin	P08697	Protease inhibitor	[7]
Alpha-2-HS-glycoprotein	P02765	Anti-inflammatory effect	[8]
Alpha-2-macroglobulin	P01023	Protease inhibitor	[9]
Alpha-2-macroglobulin-like protein 1	A8K2U0	Protease inhibitor	[10]
Alpha-amylase 1A	P0DUB6	Regulation of biofilm formation	[11]
Alpha-amylase 1B	P0DTE7	Regulation of biofilm formation	[11]
Alpha-amylase 1C	P0DTE8	Regulation of biofilm formation	[11]
Alpha-amylase 2B	P19961	Regulation of biofilm formation	[11]
Aminopeptidase B	Q9H4A4	Exopeptidase activity	[12]
Aminopeptidase N	P15144	Exopeptidase activity	[13]
Amyloid-beta precursor protein	P05067	Antimicrobial activity	[15]

Angiogenin	P03950	Antimicrobial activity	[16]
Antileukoproteinase	P03973	Protease inhibitor<br>Immunomodulatory effect	[17,18]
Antithrombin-III	P01008	Protease inhibitor	[19]
Apolipoprotein A-I	P02647	Antimicrobial activity	[20]
Apolipoprotein A-II	P02652	Immunomodulatory effect	[21]
Apolipoprotein A-IV	P06727	Immunomodulatory effect	[22]
Apolipoprotein B-100	P04114	Antimicrobial activity	[23]
Apolipoprotein C-III	P02656	Immunomodulatory effect	[24]
Apolipoprotein D	P05090	Immunomodulatory effect	[26]
Apolipoprotein E	P02649	Immunomodulatory effect	[27]
Apolipoprotein L1	O14791	Immunomodulatory effect	[28]
Apolipoprotein M	O95445	Immunomodulatory effect	[29]
Arginase-1	P05089	Antifungal activity<br>Immunomodulatory effect	[30,31]
Aspartyl aminopeptidase	Q9ULA0	Exopeptidase activity	[32]
Azurocidin	P20160	Antimicrobial activity	[33]
Bactericidal permeability-increasing protein	P17213	Antimicrobial activity	[33,34]
Beta-2-glycoprotein 1	P02749	Immunomodulatory effect	[35]
Beta-2-microglobulin	P61769	Antimicrobial activity<br>Immunomodulatory effect	[36,37]
Beta-Ala-His dipeptidase	Q96KN2	Carboxypeptidase activity	[38]
Beta-defensin 1	P60022	Antimicrobial activity	[33]
Beta-defensin 4A	O15263	Antimicrobial activity	[33]
Beta-hexosaminidase subunit alpha	P06865	Antimicrobial activity	[39]
Beta-hexosaminidase subunit beta	P07686	Antimicrobial activity	[39]
Bone marrow stromal antigen 2	Q10589	Antiviral effect	[40]
BPI fold-containing family A member 1	Q9NP55	Antimicrobial activity	[41]
BPI fold-containing family A member 2	Q96DR5	Antimicrobial activity	[42]
BPI fold-containing family B member 1	Q8TDL5	Antimicrobial activity	[44]
BPI fold-containing family B member 2	Q8N4F0	Antimicrobial activity	[44]
Brain-specific serine protease 4	Q9GZN4	Serine protease activity	[47]
Cactin	Q8WUQ7	Immunomodulatory effect	[250]
Calcitonin gene-related peptide 1	P06881	Antimicrobial activity	[48]
Calpain-1 catalytic subunit	P07384	Endopeptidase activity<br>Immunomodulatory effect	[49]
Calpain-10	Q9HC96	Endopeptidase activity<br>Immunomodulatory effect	[49]
Calpain-12	Q6ZSI9	Endopeptidase activity<br>Immunomodulatory effect	[49]
Calpain-2 catalytic subunit	P17655	Endopeptidase activity<br>Immunomodulatory effect	[49]
Calpain-5	O15484	Endopeptidase activity<br>Immunomodulatory effect	[49]
Calpain-6	Q9Y6Q1	Endopeptidase activity<br>Immunomodulatory effect	[49]

Calpain-7	Q9Y6W3	Endopeptidase activity Immunomodulatory effect	[49]
Calpastatin	P20810	Protease inhibitor	[50]
Carboxypeptidase A1	P15085	Carboxypeptidase activity	[51]
Carboxypeptidase A4	Q9UI42	Carboxypeptidase activity	[51]
Carboxypeptidase A6	Q8N4T0	Carboxypeptidase activity	[51]
Carboxypeptidase B	P15086	Carboxypeptidase activity	[52]
Carboxypeptidase B2	Q96IY4	Carboxypeptidase activity	[52]
Carboxypeptidase D	O75976	Carboxypeptidase activity	[53]
Carboxypeptidase E	P16870	Carboxypeptidase activity	[53]
Carboxypeptidase M	P14384	Carboxypeptidase activity	[54]
Carboxypeptidase N catalytic chain	P15169	Carboxypeptidase activity	[55]
Carboxypeptidase Q	Q9Y646	Carboxypeptidase activity	[56]
Carboxypeptidase Z	Q66K79	Carboxypeptidase activity	[251]
Carcinoembryonic antigen-related cell adhesion molecule 1	P13688	Immunomodulatory effect	[57]
Carcinoembryonic antigen-related cell adhesion molecule 3	P40198	Immunomodulatory effect	[58]
Carcinoembryonic antigen-related cell adhesion molecule 4	O75871	Immunomodulatory effect	[252]
Catalase	P04040	Antimicrobial activity	[59]
Cathelicidin antimicrobial peptide	P49913	Antimicrobial activity	[33]
Cathepsin B	P07858	Endopeptidase activity	[60]
Cathepsin D	P07339	Endopeptidase activity	[60]
Cathepsin E	P14091	Endopeptidase activity	[60]
Cathepsin F	Q9UBX1	Endopeptidase activity	[60]
Cathepsin G	P08311	Endopeptidase activity	[60]
Cathepsin K	P43235	Endopeptidase activity	[60]
Cathepsin L2	O60911	Endopeptidase activity	[60]
Cathepsin O	P43234	Endopeptidase activity	[60]
Cathepsin S	P25774	Endopeptidase activity	[60]
Cathepsin Z	Q9UBR2	Endopeptidase activity	[60]
Cell surface glycoprotein MUC18	P43121	Immunomodulatory effect	[61]
Ceruloplasmin	P00450	Cu^{2+} sequestering activity	[62]
Chitinase-3-like protein 1	P36222	Antimicrobial activity	[63]
Chitotriosidase-1	Q13231	Antifungal activity	[64]
Chromogranin-A	P10645	Processed forms have anti-microbial activity	[65]
Clusterin	P10909	Immunomodulatory effect	[66]
Collagen alpha-1(XII) chain	Q99715	Immunomodulatory effect	[67]
Core histone macro-H2A.1	O75367	Antimicrobial activity	[68]
Core histone macro-H2A.2	Q9P0M6	Antimicrobial activity	[68]
Corticosteroid-binding globulin	P08185	Protease inhibitor	[69]
C-reactive protein	P02741	Acute phase protein	[70]

Cystatin-A	P01040	Protease inhibitor	[71]
Cystatin-B	P04080	Protease inhibitor	[71]
Cystatin-C	P01034	Protease inhibitor	[71]
Cystatin-D	P28325	Protease inhibitor	[71]
Cystatin-F	O76096	Protease inhibitor	[71]
Cystatin-M	Q15828	Protease inhibitor	[71]
Cystatin-S	P01036	Protease inhibitor	[71]
Cystatin-SA	P09228	Protease inhibitor	[71]
Cystatin-SN	P01037	Protease inhibitor	[71]
Cytosol aminopeptidase	P28838	Aminopeptidase activity	[72]
Cytosolic non-specific dipeptidase	Q96KP4	Carboxypeptidase activity	[75]
Defensin-5	Q01523	Antimicrobial activity	[33]
Defensin-6	Q01524	Antimicrobial activity	[33]
Deleted in malignant brain tumors 1 protein	Q9UGM3	Immunomodulatory effect Antimicrobial activity	[76,77]
Deoxyribonuclease-1	P24855	Endonuclease activity	[78]
Dermcidin	P81605	Antimicrobial activity	[33]
Dipeptidase 1	P16444	Carboxypeptidase activity	[79]
Dipeptidase 2	Q9H4A9	Carboxypeptidase activity	[80]
Dipeptidyl peptidase 1	P53634	Carboxypeptidase activity	[81]
Dipeptidyl peptidase 2	Q9UHL4	Carboxypeptidase activity	[82]
Dipeptidyl peptidase 3	Q9NY33	Carboxypeptidase activity	[83]
Dipeptidyl peptidase 4	P27487	Carboxypeptidase activity	[84]
Dipeptidyl peptidase 9	Q86TI2	Carboxypeptidase activity	[85]
Disintegrin and metalloproteinase domain-containing protein 10	O14672	Metalloendopeptidase activity	[86]
Disintegrin and metalloproteinase domain-containing protein 15	Q13444	Metalloendopeptidase activity	[86]
Disintegrin and metalloproteinase domain-containing protein 17	P78536	Metalloendopeptidase activity	[86]
Disintegrin and metalloproteinase domain-containing protein 19	Q9H013	Metalloendopeptidase activity	[86]
Disintegrin and metalloproteinase domain-containing protein 22	Q9P0K1	Metalloendopeptidase activity	[86]
Disintegrin and metalloproteinase domain-containing protein 23	O75077	Metalloendopeptidase activity	[86]
Disintegrin and metalloproteinase domain-containing protein 33	Q9BZ11	Metalloendopeptidase activity	[86]
Disintegrin and metalloproteinase domain-containing protein 8	P78325	Metalloendopeptidase activity	[86]
Disintegrin and metalloproteinase domain-containing protein 9	Q13443	Metalloendopeptidase activity	[86]
Drebrin-like protein	Q9UJU6	Immunomodulatory effect	[87]
Elafin	P19957	Protease inhibitor	[88]

Endoplasmic reticulum aminopeptidase 1	Q9NZ08	Aminopeptidase activity	[89]
Endoplasmic reticulum aminopeptidase 2	Q6P179	Aminopeptidase activity	[89]
Eosinophil cationic protein	P12724	Antimicrobial activity	[90]
Eosinophil peroxidase	P11678	Antimicrobial activity	[91]
Extracellular glycoprotein lacritin	Q9GZZ8	Antimicrobial activity	[92]
Fatty acid-binding protein 4	P15090	Immunomodulatory effect	[93]
Fatty acid-binding protein 5	Q01469	Immunomodulatory effect	[94]
Fibrinogen alpha chain	P02671	Immunomodulatory effect	[95]
Fibrinogen beta chain	P02675	Immunomodulatory effect	[95]
Fibrinogen gamma chain	P02679	Immunomodulatory effect	[95]
Fibroleukin	Q14314	Immunomodulatory effect	[97]
Furin	P09958	Serine protease activity	[99]
Galectin-1	P09382	Immunomodulatory effect	[101]
Galectin-10	Q05315	Immunomodulatory effect	[102]
Galectin-2	P09958	Immunomodulatory effect	[253]
Galectin-3	P17931	Immunomodulatory effect	[103]
Galectin-3-binding protein	Q08380	Antimicrobial activity Immunomodulatory effect	[104,105]
Galectin-7	P47929	Immunomodulatory effect	[106]
Galectin-9	O00182	Immunomodulatory effect	[107]
Gastricsin	P20142	Aspartic-type endopeptidase activity	[108]
Gelsolin	P06396	Processed from has antimicrobial activity	[109]
Glia-derived nexin	P07093	Protease inhibitor	[110]
Glucose-6-phosphate isomerase	P06744	Induces immunoglobulin secretion	[111]
Glutamate carboxypeptidase 2	Q04609	Carboxypeptidase activity	[112]
Glutamyl aminopeptidase	Q07075	Aminopeptidase activity	[113]
Glutathione S-transferase omega-1	P78417	Immunomodulatory effect	[114]
Glutathione S-transferase P	P09211	Immunomodulatory effect	[115]
Glyceraldehyde-3-phosphate dehydrogenase	P04406	Immunomodulatory effect	[116]
Growth-regulated alpha protein	P09341	Antimicrobial activity	[118]
Guanylate-binding protein 1	P32455	Immunomodulatory effect	[119]
Guanylate-binding protein 2	P32456	Antiviral effect	[120]
Guanylate-binding protein 4	Q96PP9	Immunomodulatory effect	[119]
Haptoglobin	P00738	Immunomodulatory effect Iron sequestrating	[122]
Haptoglobin-related protein	P00739	Anti-parasitic effect	[123]
Heme-binding protein 1	Q9NRV9	Heme/iron sequestration	[124]
Heme-binding protein 2	Q9Y5Z4	Heme/iron sequestration	[124]
Hemoglobin subunit alpha	P69905	Processed forms (hemocidins) have antimicrobial activity	[125]
Hemoglobin subunit beta	P68871	Processed forms (hemo-	[125]

		cidins) have antimicrobial activity	
Hemopexin	P02790	Antibacterail effect<br>Anti-inflammatory effect	[126]
Heparin cofactor 2	P05546	Protease inhibitor	[127]
Hepcidin	P81172	Antimicrobial activity<br>Iron sequestration	[128,129]
High mobility group protein B1	P09429	Immunomodulatory effect	[130]
High mobility group protein B2	P26583	Antimicrobial activity	[131]
Histidine-rich glycoprotein	P04196	Antimicrobial activity	[133]
Histone H1.0	P07305	Antimicrobial activity	[134]
Histone H1.1	Q02539	Antimicrobial activity	[134]
Histone H1.10	Q92522	Antimicrobial activity	[134]
Histone H1.2	P16403	Antimicrobial activity	[134]
Histone H1.3	P16402	Antimicrobial activity	[134]
Histone H1.4	P10412	Antimicrobial activity	[134]
Histone H1.5	P16401	Antimicrobial activity	[134]
Histone H1t	P22492	Antimicrobial activity	[134]
Histone H2A type 1	P0C0S8	Antimicrobial activity	[134]
Histone H2A type 1-A	Q96QV6	Antimicrobial activity	[134]
Histone H2A type 1-B/E	P04908	Antimicrobial activity	[134]
Histone H2A type 1-C	Q93077	Antimicrobial activity	[134]
Histone H2A type 1-D	P20671	Antimicrobial activity	[134]
Histone H2A type 1-H	Q96KK5	Antimicrobial activity	[134]
Histone H2A type 1-J	Q99878	Antimicrobial activity	[134]
Histone H2A type 2-A	Q6FI13	Antimicrobial activity	[134]
Histone H2A type 2-B	Q8IUE6	Antimicrobial activity	[134]
Histone H2A type 2-C	Q16777	Antimicrobial activity	[134]
Histone H2A type 3	Q7L7L0	Antimicrobial activity	[134]
Histone H2A.J	Q9BTM1	Antimicrobial activity	[134]
Histone H2A.V	Q71UI9	Antimicrobial activity	[134]
Histone H2A.Z	P0C0S5	Antimicrobial activity	[134]
Histone H2A-Bbd type 1	P0C5Y9	Antimicrobial activity	[134]
Histone H2AX	P16104	Antimicrobial activity	[134]
Histone H2B type 1-A	Q96A08	Antimicrobial activity	[134]
Histone H2B type 1-B	P33778	Antimicrobial activity	[134]
Histone H2B type 1-C/E/F/G/I	P62807	Antimicrobial activity	[134]
Histone H2B type 1-D	P58876	Antimicrobial activity	[134]
Histone H2B type 1-H	Q93079	Antimicrobial activity	[134]
Histone H2B type 1-J	P06899	Antimicrobial activity	[134]
Histone H2B type 1-K	O60814	Antimicrobial activity	[134]
Histone H2B type 1-L	Q99880	Antimicrobial activity	[134]
Histone H2B type 1-M	Q99879	Antimicrobial activity	[134]
Histone H2B type 1-N	Q99877	Antimicrobial activity	[134]

Histone H2B type 1-O	P23527	Antimicrobial activity	[134]
Histone H2B type 2-E	Q16778	Antimicrobial activity	[134]
Histone H2B type 2-F	Q5QNW6	Antimicrobial activity	[134]
Histone H2B type 3-B	Q8N257	Antimicrobial activity	[134]
Histone H2B type F-S	P57053	Antimicrobial activity	[134]
Histone H3.1	P68431	Antimicrobial activity	[134]
Histone H3.1t	Q16695	Antimicrobial activity	[134]
Histone H3.2	Q71DI3	Antimicrobial activity	[134]
Histone H3.3	P84243	Antimicrobial activity	[134]
Histone H3.3C	Q6NXT2	Antimicrobial activity	[134]
Histone H3-7	Q5TEC6	Antimicrobial activity	[134]
Histone H4	P62805	Antimicrobial activity	[134]
Inter-alpha-trypsin inhibitor heavy chain H1	P19827	Protease inhibitor	[135]
Inter-alpha-trypsin inhibitor heavy chain H2	P19823	Protease inhibitor	[135]
Inter-alpha-trypsin inhibitor heavy chain H3	Q06033	Protease inhibitor	[135]
Inter-alpha-trypsin inhibitor heavy chain H4	Q14624	Protease inhibitor	[135]
Inter-alpha-trypsin inhibitor heavy chain H5	Q86UX2	Protease inhibitor	[135]
Interferon-induced 35 kDa protein	P80217	Immunomodulatory effect	[136]
Kallikrein-10	O43240	Serine protease activity	[138]
Kallikrein-11	Q9UBX7	Serine protease activity	[138]
Kallikrein-12	Q9UKR0	Serine protease activity	[138]
Kallikrein-13	Q9UKR3	Serine protease activity	[138]
Kallikrein-14	Q9P0G3	Serine protease activity	[138]
Kallikrein-2	P20151	Serine protease activity	[138]
Kallikrein-3	P07288	Serine protease activity	[138]
Kallikrein-5	Q9Y337	Serine protease activity<br>Processing the maturation of LL-37 cathelicidin	[138,139]
Kallikrein-6	Q92876	Serine protease activity	[138]
Kallikrein-7	P49862	Serine protease activity<br>Processing the maturation of LL-37 cathelicidin	[138]
Kallikrein-8	O60259	Serine protease activity	[138]
Kininogen-1	P01042	Antimicrobial activity	[140,141]
Kunitz-type protease inhibitor 1	O43278	Protease inhibitor	[142]
Kunitz-type protease inhibitor 2	O43291	Protease inhibitor	[142]
Kunitz-type protease inhibitor 3	P49223	Protease inhibitor	[142]
Lactoperoxidase	P22079	Antimicrobial activity	[143]
Lactotransferrin	P02788	Antimicrobial activity<br>Iron sequestration	[33]
Legumain	Q99538	Endopeptidase activity	[144]
Leukocyte elastase inhibitor	P30740	Protease inhibitor	[145]
Lipocalin-1	P31025	Immunomodulatory effect<br>Iron sequestration	[146,147]

Lipocalin-2	P80188	Immunomodulatory effect<br>Iron sequestration	[146,147]
Lipopolysaccharide-binding protein	P18428	Immunomodulatory effect	[148]
Liver-expressed antimicrobial peptide 2	Q969E1	Antimicrobial activity	[149]
Lysozyme C	P61626	Antimicrobial activity	[33]
Macrophage migration inhibitory factor	P14174	Antimicrobial activity	[151]
Major vault protein	Q14764	Immunomodulatory effect	[152]
Mammaglobin-B	O75556	Immunomodulatory effect	[153]
Matrix metalloproteinase-9	P14780	Metalloprotease activity	[155]
Melanotransferrin	P08582	Iron sequestration	[156]
Metalloproteinase inhibitor 1	P01033	Protease Inhibitor	[157]
Metalloproteinase inhibitor 2	P16035	Protease Inhibitor	[157]
Metalloproteinase inhibitor 3	P35625	Protease Inhibitor	[157]
Metalloproteinase inhibitor 4	Q99727	Protease Inhibitor	[157]
Midkine	P21741	Immunomodulatory effect	[158]
Moesin	P26038	Immunomodulatory effect	[159]
Mucin-1	P15941	Antimicrobial activity	[160]
Mucin-13	Q9H3R2	Antimicrobial activity	[160]
Mucin-16	Q8WXI7	Antimicrobial activity	[160]
Mucin-2	Q02817	Antimicrobial activity	[160]
Mucin-4	Q99102	Antimicrobial activity	[160]
Mucin-5AC	P98088	Antimicrobial activity	[160]
Mucin-5B	Q9HC84	Antimicrobial activity	[160]
Mucin-6	Q6W4X9	Antimicrobial activity	[160]
Mucin-7	Q8TAX7	Antimicrobial activity	[160]
Myeloblastin	P24158	Serine protease activity	[161]
Myeloperoxidase	P05164	Antimicrobial activity	[162]
Myoglobin	P02144	Processed forms (hemocidins) have antimicrobial activity	[163]
N-acetylmuramoyl-L-alanine amidase	Q96PD5	Antimicrobial activity	[164]
Neprilysin	P08473	Endopeptidase activity	[165]
Neutrophil collagenase	P22894	Endopeptidase activity<br>Immunomodulatory effect	[166]
Neutrophil defensin 1	P59665	Antimicrobial activity	[33]
Neutrophil defensin 3	P59666	Antimicrobial activity	[33]
Neutrophil defensin 4	P12838	Antimicrobial activity	[33]
Neutrophil elastase	P08246	Serine protease activity	[167]
Nicotinamide phosphoribosyltransferase	P43490	Immunomodulatory effect	[168]
Non-histone chromosomal protein HMG-17	P05204	Antimicrobial activity	[169]
Non-secretory ribonuclease	P10153	Ribonuclease activity	[170]
Peptidase inhibitor 16	Q6UXB8	Protease inhibitor	[172]
Peptidoglycan recognition protein 1	O75594	Antimicrobial activity	[173]
Phospholipase B-like 1	Q6P4A8	Suggested antimicrobial activity	[175]

Pigment epithelium-derived factor	P36955	Protease inhibitor	[176]
Plasma kallikrein	P03952	Serine protease activity	[138]
Plasma serine protease inhibitor	P05154	Protease inhibitor	[177]
Plastin-2	P13796	Immunomodulatory effect	[178]
Poly(rC)-binding protein 1	Q15365	Antiviral effect	[179]
Poly(rC)-binding protein 2	Q15366	Antiviral effect	[180]
Pregnancy zone protein	P20742	Protease inhibitor	[181]
Pro-adrenomedullin	P35318	Antimicrobial activity<br>Immunomodulatory effect	[182,183]
Pro-cathepsin H	P09668	Endopeptidase activity	[60]
Procathepsin L	P07711	Endopeptidase activity	[60]
Progranulin	P28799	Immunomodulatory effect	[184]
Prolactin-inducible protein	P12273	Aspartic-type endopeptidase activity<br>Modulates the activity of Zn-α2 glycoprotein	[185,186]
Proline-rich protein 15	Q8IV56	Antimicrobial activity	[187]
Proline-rich protein 4	Q16378	Antimicrobial activity	[187]
Prolyl endopeptidase	P48147	Endopeptidase activity	[188]
Prosalusin	Q8N2E6	Antimicrobial activity	[190]
Prosaposin	P07602	Processed forms has antimicrobial effect	[191]
Prostasin	Q16651	Serine protease activity	[192]
Protein AMBP	P02760	Protease inhibitor	[193]
Protein FAM3A	P98173	Antifungal effect	[194]
Protein S100-A1	P23297	Immunomodulatory effect	[195]
Protein S100-A10	P60903	Immunomodulatory effect	[196]
Protein S100-A11	P31949	Immunomodulatory effect	[197]
Protein S100-A12	P80511	Immunomodulatory effect	[196]
Protein S100-A13	Q99584	Immunomodulatory effect	[198]
Protein S100-A14	Q9HCY8	Immunomodulatory effect	[199]
Protein S100-A2	P29034	Immunomodulatory effect	[200]
Protein S100-A4	P26447	Immunomodulatory effect	[196]
Protein S100-A6	P06703	Immunomodulatory effect	[201]
Protein S100-A7	P31151	Immunomodulatory effect	[196]
Protein S100-A8	P05109	Immunomodulatory effect	[196]
Protein S100-A9	P06702	Immunomodulatory effect	[196]
Protein S100-P	P25815	Immunomodulatory effect	[196]
Puromycin-sensitive aminopeptidase	P55786	Aminopeptidase activity	[203]
RelA-associated inhibitor	Q8WUF5	Antiviral effect<br>Immunomodulatory effect	[204]
Retroviral-like aspartic protease 1	Q53RT3	Aspartic-type endopeptidase activity	[205]
Ribonuclease 4	P34096	Ribonuclease activity	[170]
Ribonuclease 7	Q9H1E1	Ribonuclease activity	[170]

Ribonuclease K6	Q93091	Ribonuclease activity	[170]
Ribonuclease pancreatic	P07998	Ribonuclease activity	[170]
Ribonuclease T2	O00584	Ribonuclease activity	[170]
Secreted Ly-6/uPAR domain-containing protein 2	P0DP57	Immunomodulatory effect	[247]
Secreted Ly-6/uPAR-related protein 1	P55000	Immunomodulatory effect	[206]
Secretoglobin family 1D member 2	O95969	Immunomodulatory effect	[207]
Secretoglobin family 3A member 1	Q96QR1	Immunomodulatory effect	[208]
Semenogelin-1	P04279	Processed forms has antimicrobial activity	[209–211]
Semenogelin-2	Q02383	Processed forms has antimicrobial activity	[209–211]
Serine protease 1	P07477	Serine protease activity	[212]
Serine protease 23	O95084	Serine protease activity	[212]
Serine protease 27	Q9BQR3	Serine protease activity	[212]
Serine protease 53	Q2L4Q9	Serine protease activity	[212]
Serine protease 58	Q8IYP2	Serine protease activity	[212]
Serine protease HTRA1	Q92743	Serine protease activity	[212]
Serine protease HTRA2	O43464	Serine protease activity	[212]
Serine protease HTRA3	P83110	Serine protease activity	[212]
Serine protease inhibitor Kazal-type 1	P00995	Protease inhibitor	[213]
Serine protease inhibitor Kazal-type 2	P20155	Protease inhibitor	[213]
Serine protease inhibitor Kazal-type 5	Q9NQ38	Protease inhibitor	[213]
Serine protease inhibitor Kazal-type 7	P58062	Protease inhibitor	[213]
Serotransferrin	P02787	Iron sequestration	[214]
Serpin B10	P48595	Protease inhibitor	[215]
Serpin B12	Q96P63	Protease inhibitor	[215]
Serpin B13	Q9UIV8	Protease inhibitor	[215]
Serpin B3	P29508	Protease inhibitor	[215]
Serpin B4	P48594	Protease inhibitor	[215]
Serpin B5	P36952	Protease inhibitor	[215]
Serpin B6	P35237	Protease inhibitor	[215]
Serpin B7	O75635	Protease inhibitor	[215]
Serpin B8	P50452	Protease inhibitor	[215]
Serpin B9	P50453	Protease inhibitor	[215]
Serum amyloid A-1 protein	P0DJI8	Immunomodulatory effect	[216]
Serum amyloid A-2 protein	P0DJI9	Immunomodulatory effect	[216]
Serum amyloid A-4 protein	P35542	Immunomodulatory effect	[216]
Serum amyloid P-component	P02743	Antiviral effect	[217]
Sialomucin core protein 24	Q04900	Immunomodulatory effect	[218]
Small proline-rich protein 3	Q9UBC9	Antimicrobial effect	[187]
Syntenin-1	O00560	Immunomodulatory effect	[219]
T-cell immunomodulatory protein	Q8TB96	Immunomodulatory effect	[220]
Thioredoxin domain-containing protein 17	Q9BRA2	Immunomodulatory effect	[221]

Thymosin beta-10	P63313	Antimicrobial activity	[222]
Thymosin beta-4	P62328	Antimicrobial activity	[222]
Thyroxine-binding globulin	P05543	Protease inhibitor	[223]
Toll-interacting protein	Q9H0E2	Immunomodulatory effect	[224]
Transgelin	Q01995	Immunomodulatory effect	[225]
Transgelin-2	P37802	Immunomodulatory effect	[226]
Transmembrane protease serine 11A	Q6ZMR5	Serine protease activity	[227]
Transmembrane protease serine 11B	Q86T26	Serine protease activity	[248]
Transmembrane protease serine 11D	O60235	Serine protease activity	[228]
Transmembrane protease serine 11E	Q9UL52	Serine protease activity	[229]
Triokinase/FMN cyclase	Q3LXA3	Immunomodulatory effect	[230]
Tripeptidyl-peptidase 1	O14773	Serine protease activity	[231]
Tripeptidyl-peptidase 2	P29144	Serine protease activity	[232]
Trypsin-2	P07478	Serine protease activity	[233]
Trypsin-3	P35030	Serine protease activity	[234]
Tryptase alpha/beta-1	Q15661	Serine protease activity	[234]
Tryptase beta-2	P20231	Serine protease activity	[234]
Tryptase delta	Q9BZJ3	Serine protease activity	[234]
Tryptase gamma	Q9NRR2	Serine protease activity	[234]
Uromodulin	P07911	Antimicrobial activity	[236,237]
Uteroglobin	P11684	Immunomodulatory effect	[238]
Vitamin D-binding protein	P02774	Immunomodulatory effect	[239]
WAP four-disulfide core domain protein 1	Q9HC57	Protease inhibitor	[240]
WAP four-disulfide core domain protein 12	Q8WWY7	Protease inhibitor	[240]
WAP four-disulfide core domain protein 2	Q14508	Protease inhibitor	[240]
WAP, Kazal, immunoglobulin, Kunitz and NTR domain-containing protein 1	Q96NZ8	Protease inhibitor	[240]
WAP, Kazal, immunoglobulin, Kunitz and NTR domain-containing protein 2	Q8TEU8	Protease inhibitor	[240]
Xaa-Pro aminopeptidase 1	Q9NQW7	Aminopeptidase activity	[241]
Xaa-Pro aminopeptidase 2	O43895	Aminopeptidase activity	[241]
Xaa-Pro aminopeptidase 3	Q9NQH7	Aminopeptidase activity	[241]
Xaa-Pro dipeptidase	P12955	Carboxypeptidase activity	[242]
Zinc-alpha-2-glycoprotein	P25311	Immunomodulatory effect	[243]
Zymogen granule membrane protein 16	O60844	Antimicrobial activity	[244]
Zymogen granule protein 16 homolog B	Q96DA0	Antimicrobial activity	[245]

Table S7. Proteins involved in the first line of host defense in the cervicovaginal fluid.

Protein name	UniProt entry	Function	Reference
Alpha-1-acid glycoprotein 1	P02763	Immunomodulatory effect	[3]
Alpha-1-acid glycoprotein 2	P19652	Immunomodulatory effect	[3]
Alpha-1-antichymotrypsin	P01011	Protease inhibitor	[4]

Alpha-1-antitrypsin	P01009	Protease inhibitor	[5]
Alpha-1B-glycoprotein	P04217	Immunomodulatory effect	[6]
Alpha-2-antiplasmin	P08697	Protease inhibitor	[7]
Alpha-2-HS-glycoprotein	P02765	Anti-inflammatory effect	[8]
Alpha-2-macroglobulin	P01023	Protease inhibitor	[9]
Alpha-2-macroglobulin-like protein 1	A8K2U0	Protease inhibitor	[10]
Alpha-amylase 1A	P0DUB6	Regulation of biofilm formation	[11]
Alpha-amylase 1B	P0DTE7	Regulation of biofilm formation	[11]
Alpha-amylase 1C	P0DTE8	Regulation of biofilm formation	[11]
Alpha-amylase 2B	P19961	Regulation of biofilm formation	[11]
Aminopeptidase N	P15144	Exopeptidase activity	[13]
Angiogenin	P03950	Antimicrobial activity	[16]
Antileukoproteinase	P03973	Protease inhibitor<br>Immunomodulatory effect	[17,18]
Antithrombin-III	P01008	Protease inhibitor	[19]
Apolipoprotein A-I	P02647	Antimicrobial activity	[20]
Apolipoprotein A-II	P02652	Immunomodulatory effect	[21]
Apolipoprotein A-IV	P06727	Immunomodulatory effect	[22]
Apolipoprotein B-100	P04114	Antimicrobial activity	[23]
Apolipoprotein L1	O14791	Immunomodulatory effect	[28]
Arginase-1	P05089	Antifungal activity<br>Immunomodulatory effect	[30,31]
Azurocidin	P20160	Antimicrobial activity	[33]
Bactericidal permeability-increasing protein	P17213	Antimicrobial activity	[33,34]
Beta-2-glycoprotein 1	P02749	Immunomodulatory effect	[35]
Beta-2-microglobulin	P61769	Antimicrobial activity<br>Immunomodulatory effect	[36,37]
Beta-hexosaminidase subunit beta	P07686	Antimicrobial activity	[39]
BPI fold-containing family A member 1	Q9NP55	Antimicrobial activity	[41]
BPI fold-containing family B member 1	Q8TDL5	Antimicrobial activity	[44]
BPI fold-containing family B member 2	Q8N4F0	Antimicrobial activity	[44]
Calpain-1 catalytic subunit	P07384	Endopeptidase activity<br>Immunomodulatory effect	[49]
Calpastatin	P20810	Protease inhibitor	[50]
Carboxypeptidase A4	Q9UI42	Carboxypeptidase activity	[51]
Carboxypeptidase E	P16870	Carboxypeptidase activity	[53]
Carboxypeptidase M	P14384	Carboxypeptidase activity	[54]
Carcinoembryonic antigen-related cell adhesion molecule 1	P13688	Immunomodulatory effect	[57]
Catalase	P04040	Antimicrobial activity	[59]
Cathelicidin antimicrobial peptide	P49913	Antimicrobial activity	[33]

Cathepsin B	P07858	Endopeptidase activity	[60]
Cathepsin D	P07339	Endopeptidase activity	[60]
Cathepsin G	P08311	Endopeptidase activity	[60]
Cathepsin L2	O60911	Endopeptidase activity	[60]
Cathepsin S	P25774	Endopeptidase activity	[60]
Cathepsin Z	Q9UBR2	Endopeptidase activity	[60]
Ceruloplasmin	P00450	Cu^{2+} sequestering activity	[62]
Chitinase-3-like protein 1	P36222	Antimicrobial activity	[63]
Chitotriosidase-1	Q13231	Antifungal activity	[64]
Chromogranin-A	P10645	Processed forms have anti-microbial activity	[65]
Clusterin	P10909	Immunomodulatory effect	[66]
Collagen alpha-1(XII) chain	Q99715	Immunomodulatory effect	[67]
Core histone macro-H2A.1	O75367	Antimicrobial activity	[68]
Corticosteroid-binding globulin	P08185	Protease inhibitor	[69]
Cystatin-A	P01040	Protease inhibitor	[71]
Cystatin-B	P04080	Protease inhibitor	[71]
Cystatin-C	P01034	Protease inhibitor	[71]
Cystatin-M	Q15828	Protease inhibitor	[71]
Cystatin-S	P01036	Protease inhibitor	[71]
Cystatin-SN	P01037	Protease inhibitor	[71]
Cytosolic non-specific dipeptidase	Q96KP4	Carboxypeptidase activity	[75]
Deleted in malignant brain tumors 1 protein	Q9UGM3	Immunomodulatory effect<br>Antimicrobial activity	[76,77]
Dermcidin	P81605	Antimicrobial activity	[33]
Dipeptidyl peptidase 1	P53634	Carboxypeptidase activity	[81]
Dipeptidyl peptidase 2	Q9UHL4	Carboxypeptidase activity	[82]
Dipeptidyl peptidase 3	Q9NY33	Carboxypeptidase activity	[83]
Dipeptidyl peptidase 4	P27487	Carboxypeptidase activity	[84]
Drebrin-like protein	Q9UJU6	Immunomodulatory effect	[87]
Elafin	P19957	Protease inhibitor	[88]
Eosinophil cationic protein	P12724	Antimicrobial activity	[90]
Eosinophil peroxidase	P11678	Antimicrobial activity	[91]
Fatty acid-binding protein 4	P15090	Immunomodulatory effect	[93]
Fatty acid-binding protein 5	Q01469	Immunomodulatory effect	[94]
Fibrinogen alpha chain	P02671	Immunomodulatory effect	[95]
Fibrinogen beta chain	P02675	Immunomodulatory effect	[95]
Fibrinogen gamma chain	P02679	Immunomodulatory effect	[95]
Fibrocystin-L	Q86WI1	Immunomodulatory effect	[96]
Fibroleukin	Q14314	Immunomodulatory effect	[97]
Galectin-1	P09382	Immunomodulatory effect	[101]
Galectin-10	Q05315	Immunomodulatory effect	[102]
Galectin-3	P17931	Immunomodulatory effect	[103]
Galectin-3-binding protein	Q08380	Antimicrobial activity	[104,105]

		Immunomodulatory effect	
Galectin-7	P47929	Immunomodulatory effect	[106]
Gelsolin	P06396	Processed from has antimicrobial activity	[109]
Glucose-6-phosphate isomerase	P06744	Induces immunoglobulin secretion	[111]
Glutathione S-transferase omega-1	P78417	Immunomodulatory effect	[114]
Glutathione S-transferase P	P09211	Immunomodulatory effect	[115]
Glyceraldehyde-3-phosphate dehydrogenase	P04406	Immunomodulatory effect	[116]
Growth-regulated alpha protein	P09341	Antimicrobial activity	[118]
Guanylate-binding protein 2	P32456	Immunomodulatory effect	[120]
Haptoglobin	P00738	Immunomodulatory effect<br>Iron sequestrating	[122]
Haptoglobin-related protein	P00739	Anti-parasitic effect	[123]
Heme-binding protein 2	Q9Y5Z4	Heme/iron sequestration	[124]
Hemoglobin subunit alpha	P69905	Processed forms (hemocidins) have antimicrobial activity	[125]
Hemoglobin subunit beta	P68871	Processed forms (hemocidins) have antimicrobial activity	[125]
Hemopexin	P02790	Antibacterail effect<br>Anti-inflammatory effect	[126]
Heparin cofactor 2	P05546	Protease inhibitor	[127]
High mobility group protein B1	P09429	Immunomodulatory effect	[130]
High mobility group protein B2	P26583	Antimicrobial activity	[131]
Histidine-rich glycoprotein	P04196	Antimicrobial activity	[133]
Histone H1.0	P07305	Antimicrobial activity	[134]
Histone H1.1	Q02539	Antimicrobial activity	[134]
Histone H1.2	P16403	Antimicrobial activity	[134]
Histone H1.3	P16402	Antimicrobial activity	[134]
Histone H1.4	P10412	Antimicrobial activity	[134]
Histone H1.5	P16401	Antimicrobial activity	[134]
Histone H2A type 1-C	Q93077	Antimicrobial activity	[134]
Histone H2A type 2-C	Q16777	Antimicrobial activity	[134]
Histone H2A.V	Q71UI9	Antimicrobial activity	[134]
Histone H4	P62805	Antimicrobial activity	[134]
Inter-alpha-trypsin inhibitor heavy chain H1	P19827	Protease inhibitor	[135]
Inter-alpha-trypsin inhibitor heavy chain H2	P19823	Protease inhibitor	[135]
Inter-alpha-trypsin inhibitor heavy chain H4	Q14624	Protease inhibitor	[135]
Interferon-induced 35 kDa protein	P80217	Immunomodulatory effect	[136]
Kallikrein-10	O43240	Serine protease activity	[138]
Kallikrein-11	Q9UBX7	Serine protease activity	[138]
Kallikrein-12	Q9UKR0	Serine protease activity	[138]
Kallikrein-13	Q9UKR3	Serine protease activity	[138]

Kallikrein-3	P07288	Serine protease activity	[138]
Kallikrein-6	Q92876	Serine protease activity	[138]
Kallikrein-7	P49862	Serine protease activity<br>Processing the maturation of LL-37 cathelicidin	[138]
Kallikrein-8	O60259	Serine protease activity	[138]
Kininogen-1	P01042	Antimicrobial activity	[140,141]
Kunitz-type protease inhibitor 1	O43278	Protease inhibitor	[142]
Lactotransferrin	P02788	Antimicrobial activity<br>Iron sequestration	[33]
Legumain	Q99538	Endopeptidase activity	[144]
Leukocyte elastase inhibitor	P30740	Protease inhibitor	[145]
Lipocalin-2	P80188	Immunomodulatory effect<br>Iron sequestration	[146,147]
Lipopolysaccharide-binding protein	P18428	Immunomodulatory effect	[148]
Lysozyme C	P61626	Antimicrobial activity	[33]
Macrophage migration inhibitory factor	P14174	Antimicrobial activity	[151]
Major vault protein	Q14764	Immunomodulatory effect	[152]
Mammaglobin-B	O75556	Immunomodulatory effect	[153]
Matrix metalloproteinase-9	P14780	Metalloprotease activity	[155]
Metalloproteinase inhibitor 1	P01033	Protease Inhibitor	[157]
Metalloproteinase inhibitor 2	P16035	Protease Inhibitor	[157]
Moesin	P26038	Immunomodulatory effect	[159]
Mucin-1	P15941	Antimicrobial activity	[160]
Mucin-16	Q8WXI7	Antimicrobial activity	[160]
Mucin-17	Q685J3	Antimicrobial activity	[160]
Mucin-4	Q99102	Antimicrobial activity	[160]
Mucin-5AC	P98088	Antimicrobial activity	[160]
Mucin-5B	Q9HC84	Antimicrobial activity	[160]
Mucin-6	Q6W4X9	Antimicrobial activity	[160]
Myeloblastin	P24158	Serine protease activity	[161]
Myeloperoxidase	P05164	Antimicrobial activity	[162]
N-acetylmuramoyl-L-alanine amidase	Q96PD5	Antimicrobial activity	[164]
Neprilysin	P08473	Endopeptidase activity	[165]
Neutrophil collagenase	P22894	Endopeptidase activity<br>Immunomodulatory effect	[166]
Neutrophil defensin 3	P59666	Antimicrobial activity	[33]
Neutrophil defensin 4	P12838	Antimicrobial activity	[33]
Neutrophil elastase	P08246	Serine protease activity	[167]
Nicotinamide phosphoribosyltransferase	P43490	Immunomodulatory effect	[168]
Non-secretory ribonuclease	P10153	Ribonuclease activity	[170]
Peptidoglycan recognition protein 1	O75594	Antimicrobial activity	[173]
Phospholipase B-like 1	Q6P4A8	Suggested antimicrobial activity	[175]
Pigment epithelium-derived factor	P36955	Protease inhibitor	[176]

Plasma kallikrein	P03952	Serine protease activity	[138]
Plasma serine protease inhibitor	P05154	Protease inhibitor	[177]
Plastin-2	P13796	Immunomodulatory effect	[178]
Poly(rC)-binding protein 1	Q15365	Antiviral effect	[179]
Pregnancy zone protein	P20742	Protease inhibitor	[181]
Procathepsin L	P07711	Endopeptidase activity	[60]
Progranulin	P28799	Immunomodulatory effect	[184]
Prolactin-inducible protein	P12273	Aspartic-type endopeptidase activity<br>Modulates the activity of Zn-α2 glycoprotein	[185,186]
Prosaposin	P07602	Processed forms has antimicrobial effect	[191]
Prostasin	Q16651	Serine protease activity	[192]
Protein AMBP	P02760	Protease inhibitor	[193]
Protein S100-A10	P60903	Immunomodulatory effect	[196]
Protein S100-A11	P31949	Immunomodulatory effect	[197]
Protein S100-A12	P80511	Immunomodulatory effect	[196]
Protein S100-A13	Q99584	Immunomodulatory effect	[198]
Protein S100-A14	Q9HCY8	Immunomodulatory effect	[199]
Protein S100-A2	P29034	Immunomodulatory effect	[200]
Protein S100-A4	P26447	Immunomodulatory effect	[196]
Protein S100-A7	P31151	Immunomodulatory effect	[196]
Protein S100-A8	P05109	Immunomodulatory effect	[196]
Protein S100-A9	P06702	Immunomodulatory effect	[196]
Protein S100-P	P25815	Immunomodulatory effect	[196]
Puromycin-sensitive aminopeptidase	P55786	Aminopeptidase activity	[203]
RelA-associated inhibitor	Q8WUF5	Antimicrobial activity<br>Immunomodulatory effect	[204]
Retroviral-like aspartic protease 1	Q53RT3	Aspartic-type endopeptidase activity	[205]
Ribonuclease 7	Q9H1E1	Ribonuclease activity	[170]
Ribonuclease pancreatic	P07998	Ribonuclease activity	[170]
Ribonuclease T2	O00584	Ribonuclease activity	[170]
Secreted Ly-6/uPAR domain-containing protein 2	P0DP57	Immunomodulatory effect	[247]
Secreted Ly-6/uPAR-related protein 1	P55000	Immunomodulatory effect	[206]
Secretoglobin family 1D member 2	O95969	Immunomodulatory effect	[207]
Semenogelin-1	P04279	Processed forms has antimicrobial activity	[209–211]
Semenogelin-2	Q02383	Processed forms has antimicrobial activity	[209–211]
Serine protease 27	Q9BQR3	Serine protease activity	[212]
Serine protease 57	Q6UWY2	Serine protease activity	[212]
Serine protease HTRA1	Q92743	Serine protease activity	[212]
Serine protease inhibitor Kazal-type 5	Q9NQ38	Protease inhibitor	[213]

Serine protease inhibitor Kazal-type 7	P58062	Protease inhibitor	[213]
Serotransferrin	P02787	Iron sequestration	[214]
Serpin B10	P48595	Protease inhibitor	[215]
Serpin B12	Q96P63	Protease inhibitor	[215]
Serpin B13	Q9UIV8	Protease inhibitor	[215]
Serpin B3	P29508	Protease inhibitor	[215]
Serpin B4	P48594	Protease inhibitor	[215]
Serpin B5	P36952	Protease inhibitor	[215]
Serpin B6	P35237	Protease inhibitor	[215]
Serpin B7	O75635	Protease inhibitor	[215]
Serpin B8	P50452	Protease inhibitor	[215]
Serpin B9	P50453	Protease inhibitor	[215]
Serum amyloid A-1 protein	P0DJI8	Immunomodulatory effect	[216]
Serum amyloid P-component	P02743	Antiviral effect	[217]
Small proline-rich protein 3	Q9UBC9	Antimicrobial activity	[187]
Syntenin-1	O00560	Immunomodulatory effect	[219]
Thioredoxin domain-containing protein 17	Q9BRA2	Immunomodulatory effect	[221]
Thymosin beta-10	P63313	Antimicrobial activity	[222]
Thymosin beta-4	P62328	Antimicrobial activity	[222]
Thyroxine-binding globulin	P05543	Protease inhibitor	[223]
Toll-interacting protein	Q9H0E2	Immunomodulatory effect	[224]
Transgelin-2	P37802	Immunomodulatory effect	[226]
Transmembrane protease serine 11A	Q6ZMR5	Serine protease activity	[227]
Transmembrane protease serine 11B	Q86T26	Serine protease activity	[248]
Transmembrane protease serine 11D	O60235	Serine protease activity	[228]
Transmembrane protease serine 11E	Q9UL52	Serine protease activity	[229]
Triokinase/FMN cyclase	Q3LXA3	Immunomodulatory effect	[230]
Tripeptidyl-peptidase 1	O14773	Serine protease activity	[231]
Vitamin D-binding protein	P02774	Immunomodulatory effect	[239]
WAP four-disulfide core domain protein 2	Q14508	Protease inhibitor	[240]
Zinc-alpha-2-glycoprotein	P25311	Immunomodulatory effect	[243]

Table S8. Proteins involved in the first line of host defense in the seminal fluid.

Protein name	UniProt entry	Function	Reference
Acrosin	P10323	Serine protease activity	[1]
Alpha-1-acid glycoprotein 1	P02763	Immunomodulatory effect	[3]
Alpha-1-acid glycoprotein 2	P19652	Immunomodulatory effect	[3]
Alpha-1-antichymotrypsin	P01011	Protease inhibitor	[4]
Alpha-1-antitrypsin	P01009	Protease inhibitor	[5]
Alpha-1B-glycoprotein	P04217	Immunomodulatory effect	[6]
Alpha-2-antiplasmin	P08697	Protease inhibitor	[7]
Alpha-2-macroglobulin	P01023	Protease inhibitor	[9]
Alpha-2-macroglobulin-like protein 1	A8K2U0	Protease inhibitor	[10]

Alpha-amylase 1A	P0DUB6	Regulation of biofilm formation	[11]
Alpha-amylase 1B	P0DTE7	Regulation of biofilm formation	[11]
Alpha-amylase 1C	P0DTE8	Regulation of biofilm formation	[11]
Alpha-amylase 2B	P19961	Regulation of biofilm formation	[11]
Aminopeptidase B	Q9H4A4	Exopeptidase activity	[12]
Aminopeptidase N	P15144	Exopeptidase activity	[13]
Amyloid-beta precursor protein	P05067	Antimicrobial activity	[15]
Angiogenin	P03950	Antimicrobial activity	[16]
Antileukoproteinase	P03973	Protease inhibitor<br>Immunomodulatory effect	[17,18]
Antithrombin-III	P01008	Protease inhibitor	[19]
Apolipoprotein A-I	P02647	Antimicrobial activity	[20]
Apolipoprotein A-II	P02652	Immunomodulatory effect	[21]
Apolipoprotein A-IV	P06727	Immunomodulatory effect	[22]
Apolipoprotein B-100	P04114	Antimicrobial activity	[23]
Apolipoprotein D	P05090	Immunomodulatory effect	[26]
Apolipoprotein E	P02649	Immunomodulatory effect	[27]
Arginase-1	P05089	Antifungal activity<br>Immunomodulatory effect	[30,31]
Aspartyl aminopeptidase	Q9ULA0	Exopeptidase activity	[32]
Azurocidin	P20160	Antimicrobial activity	[33]
Bactericidal permeability-increasing protein	P17213	Antimicrobial activity	[33,34]
Beta-2-glycoprotein 1	P02749	Immunomodulatory effect	[35]
Beta-2-microglobulin	P61769	Antimicrobial activity<br>Immunomodulatory effect	[36,37]
Beta-defensin 105	Q8NG35	Antimicrobial activity	[33]
Beta-defensin 106	Q8N104	Antimicrobial activity	[33]
Beta-defensin 118	Q96PH6	Antimicrobial activity	[33]
Beta-defensin 129	Q9H1M3	Antimicrobial activity	[33]
Beta-defensin 131A	P59861	Antimicrobial activity	[33]
Beta-defensin 132	Q7Z7B7	Antimicrobial activity	[33]
Beta-hexosaminidase subunit alpha	P06865	Antimicrobial activity	[39]
Beta-hexosaminidase subunit beta	P07686	Antimicrobial activity	[39]
BPI fold-containing family A member 3	Q9BQP9	Antimicrobial activity	[43]
BPI fold-containing family B member 2	Q8N4F0	Antimicrobial activity	[44]
Brain-specific serine protease 4	Q9GZN4	Serine protease activity	[47]
Calpain-1 catalytic subunit	P07384	Endopeptidase activity<br>Immunomodulatory effect	[49]
Calpain-11	Q9UMQ6	Endopeptidase activity<br>Immunomodulatory effect	[49]
Calpain-2 catalytic subunit	P17655	Endopeptidase activity<br>Immunomodulatory effect	[49]

Calpain-5	O15484	Endopeptidase activity<br>Immunomodulatory effect	[49]
Calpain-7	Q9Y6W3	Endopeptidase activity<br>Immunomodulatory effect	[49]
Calpastatin	P20810	Protease inhibitor	[50]
Carboxypeptidase A5	Q8WXQ8	Carboxypeptidase activity	[51]
Carboxypeptidase B	P15086	Carboxypeptidase activity	[52]
Carboxypeptidase D	O75976	Carboxypeptidase activity	[53]
Carboxypeptidase E	P16870	Carboxypeptidase activity	[53]
Carboxypeptidase M	P14384	Carboxypeptidase activity	[54]
Carboxypeptidase Q	Q9Y646	Carboxypeptidase activity	[56]
Carboxypeptidase Z	Q66K79	Carboxypeptidase activity	[251]
Catalase	P04040	Antimicrobial activity	[59]
Cathelicidin antimicrobial peptide	P49913	Antimicrobial activity	[33]
Cathepsin B	P07858	Endopeptidase activity	[60]
Cathepsin D	P07339	Endopeptidase activity	[60]
Cathepsin F	Q9UBX1	Endopeptidase activity	[60]
Cathepsin G	P08311	Endopeptidase activity	[60]
Cathepsin L2	O60911	Endopeptidase activity	[60]
Cathepsin O	P43234	Endopeptidase activity	[60]
Cathepsin S	P25774	Endopeptidase activity	[60]
Cathepsin Z	Q9UBR2	Endopeptidase activity	[60]
Ceruloplasmin	P00450	Cu^{2+} sequestering activity	[62]
Chitinase-3-like protein 1	P36222	Antimicrobial activity	[63]
Chromogranin-A	P10645	Processed forms have anti-microbial activity	[65]
Clusterin	P10909	Immunomodulatory effect	[66]
Collagen alpha-1(XII) chain	Q99715	Immunomodulatory effect	[67]
Core histone macro-H2A.1	O75367	Antimicrobial activity	[68]
Corticosteroid-binding globulin	P08185	Protease inhibitor	[69]
C-reactive protein	P02741	Acute phase protein	[70]
Cystatin-A	P01040	Protease inhibitor	[71]
Cystatin-B	P04080	Protease inhibitor	[71]
Cystatin-C	P01034	Protease inhibitor	[71]
Cystatin-S	P01036	Protease inhibitor	[71]
Cystatin-SA	P09228	Protease inhibitor	[71]
Cystatin-SN	P01037	Protease inhibitor	[71]
Cytosol aminopeptidase	P28838	Aminopeptidase activity	[72]
Cytosolic carboxypeptidase 1	Q9UPW5	Carboxypeptidase activity	[73]
Cytosolic non-specific dipeptidase	Q96KP4	Carboxypeptidase activity	[75]
Deoxyribonuclease-1	P24855	Endonuclease activity	[78]
Dermcidin	P81605	Antimicrobial activity	[33]
Dipeptidase 1	P16444	Carboxypeptidase activity	[79]
Dipeptidase 2	Q9H4A9	Carboxypeptidase activity	[80]

Dipeptidase 3	Q9H4B8	Carboxypeptidase activity	[80]
Dipeptidyl peptidase 1	P53634	Carboxypeptidase activity	[81]
Dipeptidyl peptidase 2	Q9UHL4	Carboxypeptidase activity	[82]
Dipeptidyl peptidase 3	Q9NY33	Carboxypeptidase activity	[83]
Dipeptidyl peptidase 4	P27487	Carboxypeptidase activity	[84]
Dipeptidyl peptidase 8	Q6V1X1	Carboxypeptidase activity	[85]
Disintegrin and metalloproteinase domain-containing protein 10	O14672	Metalloendopeptidase activity	[86]
Disintegrin and metalloproteinase domain-containing protein 29	Q9UKF5	Metalloendopeptidase activity	[86]
Disintegrin and metalloproteinase domain-containing protein 30	Q9UKF2	Metalloendopeptidase activity	[86]
Disintegrin and metalloproteinase domain-containing protein 32	Q8TC27	Metalloendopeptidase activity	[86]
Disintegrin and metalloproteinase domain-containing protein 7	Q9H2U9	Metalloendopeptidase activity	[86]
Disintegrin and metalloproteinase domain-containing protein 9	Q13443	Metalloendopeptidase activity	[86]
Drebrin-like protein	Q9UJU6	Immunomodulatory effect	[87]
Endoplasmic reticulum aminopeptidase 1	Q9NZ08	Aminopeptidase activity	[89]
Eosinophil cationic protein	P12724	Antimicrobial activity	[90]
Eosinophil peroxidase	P11678	Antimicrobial activity	[91]
Fatty acid-binding protein 5	Q01469	Immunomodulatory effect	[94]
Galectin-1	P09382	Immunomodulatory effect	[101]
Galectin-3	P17931	Immunomodulatory effect	[103]
Galectin-3-binding protein	Q08380	Antimicrobial activity<br>Immunomodulatory effect	[104,105]
Galectin-7	P47929	Immunomodulatory effect	[106]
Gastricsin	P20142	Aspartic-type endopeptidase activity	[108]
Gelsolin	P06396	Processed from has antimicrobial activity	[109]
Glucose-6-phosphate isomerase	P06744	Induces immunoglobulin secretion	[111]
Glutamate carboxypeptidase 2	Q04609	Carboxypeptidase activity	[112]
Glutathione S-transferase omega-1	P78417	Immunomodulatory effect	[114]
Glutathione S-transferase P	P09211	Immunomodulatory effect	[115]
Glyceraldehyde-3-phosphate dehydrogenase	P04406	Immunomodulatory effect	[116]
Growth-regulated alpha protein	P09341	Antimicrobial activity	[118]
Haptoglobin	P00738	Immunomodulatory effect<br>Iron sequestrating	[122]
Heme-binding protein 1	Q9NRV9	Heme/iron sequestration	[124]
Heme-binding protein 2	Q9Y5Z4	Heme/iron sequestration	[124]
Hemoglobin subunit alpha	P69905	Processed forms (hemo-	[125]

		cidins) have antimicrobial activity	
Hemoglobin subunit beta	P68871	Processed forms (hemo-cidins) have antimicrobial activity	[125]
Hemopexin	P02790	Antibacterail effect<br>Anti-inflammatory effect	[126]
High mobility group protein B1	P09429	Immunomodulatory effect	[130]
High mobility group protein B2	P26583	Antimicrobial activity	[131]
High mobility group protein B4	Q8WW32	Immunomodulatory effect	[132]
Histidine-rich glycoprotein	P04196	Antimicrobial activity	[133]
Histone H1.1	Q02539	Antimicrobial activity	[134]
Histone H1.2	P16403	Antimicrobial activity	[134]
Histone H1.3	P16402	Antimicrobial activity	[134]
Histone H1.4	P10412	Antimicrobial activity	[134]
Histone H1.5	P16401	Antimicrobial activity	[134]
Histone H1t	P22492	Antimicrobial activity	[134]
Histone H2A type 1	P0C0S8	Antimicrobial activity	[134]
Histone H2A type 1-A	Q96QV6	Antimicrobial activity	[134]
Histone H2A type 1-B/E	P04908	Antimicrobial activity	[134]
Histone H2A type 1-C	Q93077	Antimicrobial activity	[134]
Histone H2A type 1-D	P20671	Antimicrobial activity	[134]
Histone H2A type 1-H	Q96KK5	Antimicrobial activity	[134]
Histone H2A type 1-J	Q99878	Antimicrobial activity	[134]
Histone H2A type 2-A	Q6FI13	Antimicrobial activity	[134]
Histone H2A type 2-B	Q8IUE6	Antimicrobial activity	[134]
Histone H2A type 2-C	Q16777	Antimicrobial activity	[134]
Histone H2A type 3	Q7L7L0	Antimicrobial activity	[134]
Histone H2A.J	Q9BTM1	Antimicrobial activity	[134]
Histone H2A.V	Q71UI9	Antimicrobial activity	[134]
Histone H2A.Z	P0C0S5	Antimicrobial activity	[134]
Histone H2A-Bbd type 2/3	P0C5Z0	Antimicrobial activity	[134]
Histone H2AX	P16104	Antimicrobial activity	[134]
Histone H2B type 1-A	Q96A08	Antimicrobial activity	[134]
Histone H2B type 1-B	P33778	Antimicrobial activity	[134]
Histone H2B type 1-C/E/F/G/I	P62807	Antimicrobial activity	[134]
Histone H2B type 1-D	P58876	Antimicrobial activity	[134]
Histone H2B type 1-H	Q93079	Antimicrobial activity	[134]
Histone H2B type 1-J	P06899	Antimicrobial activity	[134]
Histone H2B type 1-K	O60814	Antimicrobial activity	[134]
Histone H2B type 1-L	Q99880	Antimicrobial activity	[134]
Histone H2B type 1-M	Q99879	Antimicrobial activity	[134]
Histone H2B type 1-N	Q99877	Antimicrobial activity	[134]
Histone H2B type 1-O	P23527	Antimicrobial activity	[134]
Histone H2B type 2-E	Q16778	Antimicrobial activity	[134]

Histone H2B type 2-F	Q5QNW6	Antimicrobial activity	[134]
Histone H2B type F-S	P57053	Antimicrobial activity	[134]
Histone H3.1	P68431	Antimicrobial activity	[134]
Histone H3.2	Q71DI3	Antimicrobial activity	[134]
Histone H3.3	P84243	Antimicrobial activity	[134]
Histone H3.3C	Q6NXT2	Antimicrobial activity	[134]
Histone H3-7	Q5TEC6	Antimicrobial activity	[134]
Histone H4	P62805	Antimicrobial activity	[134]
Inter-alpha-trypsin inhibitor heavy chain H5	Q86UX2	Protease inhibitor	[135]
Kallikrein-11	Q9UBX7	Serine protease activity	[138]
Kallikrein-2	P20151	Serine protease activity	[138]
Kallikrein-3	P07288	Serine protease activity	[138]
Kunitz-type protease inhibitor 1	O43278	Protease inhibitor	[142]
Kunitz-type protease inhibitor 2	O43291	Protease inhibitor	[142]
Kunitz-type protease inhibitor 3	P49223	Protease inhibitor	[142]
Kunitz-type protease inhibitor 4	Q6UDR6	Protease inhibitor	[142]
Lactotransferrin	P02788	Antimicrobial activity<br>Iron sequestration	[33]
Legumain	Q99538	Endopeptidase activity	[144]
Leukocyte elastase inhibitor	P30740	Protease inhibitor	[145]
Lipocalin-1	P31025	Immunomodulatory effect<br>Iron sequestration	[146,147]
Lipocalin-2	P80188	Immunomodulatory effect<br>Iron sequestration	[146,147]
Lysozyme C	P61626	Antimicrobial activity	[33]
Macrophage migration inhibitory factor	P14174	Antimicrobial activity	[151]
Major vault protein	Q14764	Immunomodulatory effect	[152]
Mammaglobin-B	O75556	Immunomodulatory effect	[153]
Matrix metalloproteinase-9	P14780	Metalloprotease activity	[155]
Metalloproteinase inhibitor 1	P01033	Protease Inhibitor	[157]
Metalloproteinase inhibitor 2	P16035	Protease Inhibitor	[157]
Metalloproteinase inhibitor 3	P35625	Protease Inhibitor	[157]
Midkine	P21741	Immunomodulatory effect	[158]
Moesin	P26038	Immunomodulatory effect	[159]
Mucin-15	Q8N387	Antimicrobial activity	[160]
Mucin-4	Q99102	Antimicrobial activity	[160]
Mucin-5AC	P98088	Antimicrobial activity	[160]
Mucin-5B	Q9HC84	Antimicrobial activity	[160]
Mucin-6	Q6W4X9	Antimicrobial activity	[160]
Myeloblastin	P24158	Serine protease activity	[161]
Myeloperoxidase	P05164	Antimicrobial activity	[162]
Myoglobin	P02144	Processed forms (hemo-cidins) have antimicrobial activity	[163]
Neprilysin	P08473	Endopeptidase activity	[165]

Neutrophil collagenase	P22894	Endopeptidase activity<br>Immunomodulatory effect	[166]
Neutrophil defensin 1	P59665	Antimicrobial activity	[33]
Neutrophil defensin 3	P59666	Antimicrobial activity	[33]
Neutrophil elastase	P08246	Serine protease activity	[167]
Nicotinamide phosphoribosyltransferase	P43490	Immunomodulatory effect	[168]
Non-secretory ribonuclease	P10153	Ribonuclease activity	[170]
Peptidase inhibitor 16	Q6UXB8	Protease inhibitor	[172]
Phospholipase B-like 1	Q6P4A8	Suggested antimicrobial activity	[175]
Pigment epithelium-derived factor	P36955	Protease inhibitor	[176]
Plasma serine protease inhibitor	P05154	Protease inhibitor	[177]
Plastin-2	P13796	Immunomodulatory effect	[178]
Poly(rC)-binding protein 1	Q15365	Antiviral effect	[179]
Poly(rC)-binding protein 2	Q15366	Antiviral effect	[180]
Pro-cathepsin H	P09668	Endopeptidase activity	[60]
Procathepsin L	P07711	Endopeptidase activity	[60]
Progranulin	P28799	Immunomodulatory effect	[184]
Prolactin-inducible protein	P12273	Aspartic-type endopeptidase activity<br>Modulates the activity of Zn-α2 glycoprotein	[185,186]
Proline-rich protein 30	Q53SZ7	Antimicrobial activity	[187]
Prolyl endopeptidase	P48147	Endopeptidase activity	[188]
Prosaposin	P07602	Processed forms has antimicrobial effect	[191]
Prostasin	Q16651	Serine protease activity	[192]
Protein AMBP	P02760	Protease inhibitor	[193]
Protein S100-A10	P60903	Immunomodulatory effect	[196]
Protein S100-A11	P31949	Immunomodulatory effect	[197]
Protein S100-A12	P80511	Immunomodulatory effect	[196]
Protein S100-A14	Q9HCY8	Immunomodulatory effect	[199]
Protein S100-A2	P29034	Immunomodulatory effect	[200]
Protein S100-A7	P31151	Immunomodulatory effect	[196]
Protein S100-A8	P05109	Immunomodulatory effect	[196]
Protein S100-A9	P06702	Immunomodulatory effect	[196]
Protein S100-P	P25815	Immunomodulatory effect	[196]
Protein WFDC9	Q8NEX5	Protease inhibitor	[202]
Puromycin-sensitive aminopeptidase	P55786	Aminopeptidase activity	[203]
RelA-associated inhibitor	Q8WUF5	Antiviral effect<br>Immunomodulatory effect	[204]
Ribonuclease 4	P34096	Ribonuclease activity	[170]
Ribonuclease pancreatic	P07998	Ribonuclease activity	[170]
Ribonuclease T2	O00584	Ribonuclease activity	[170]
Secretoglobin family 1D member 2	O95969	Immunomodulatory effect	[207]

Semenogelin-1	P04279	Processed forms has antimicrobial activity	[209–211]
Semenogelin-2	Q02383	Processed forms has antimicrobial activity	[209–211]
Serine protease 1	P07477	Serine protease activity	[212]
Serine protease 23	O95084	Serine protease activity	[212]
Serine protease 55	Q6UWB4	Serine protease activity	[212]
Serine protease 58	Q8IYP2	Serine protease activity	[212]
Serine protease HTRA1	Q92743	Serine protease activity	[212]
Serine protease HTRA2	O43464	Serine protease activity	[212]
Serine protease inhibitor Kazal-type 2	P20155	Protease inhibitor	[213]
Serotransferrin	P02787	Iron sequestration	[214]
Serpin B10	P48595	Protease inhibitor	[215]
Serpin B12	Q96P63	Protease inhibitor	[215]
Serpin B3	P29508	Protease inhibitor	[215]
Serpin B4	P48594	Protease inhibitor	[215]
Serpin B5	P36952	Protease inhibitor	[215]
Serpin B6	P35237	Protease inhibitor	[215]
Serpin B7	O75635	Protease inhibitor	[215]
Serpin B8	P50452	Protease inhibitor	[215]
Serpin B9	P50453	Protease inhibitor	[215]
Serum amyloid A-1 protein	P0DJI8	Immunomodulatory effect	[216]
Serum amyloid A-2 protein	P0DJI9	Immunomodulatory effect	[216]
Serum amyloid P-component	P02743	Antiviral effect	[217]
Small proline-rich protein 3	Q9UBC9	Antimicrobial effect	[187]
Sperm-associated antigen 11B	Q08648	Antimicrobial activity	[255]
Syntenin-1	O00560	Immunomodulatory effect	[219]
T-cell immunomodulatory protein	Q8TB96	Immunomodulatory effect	[220]
Testis-specific H1 histone	Q75WM6	Antimicrobial activity	[134]
Thioredoxin domain-containing protein 17	Q9BRA2	Immunomodulatory effect	[221]
Thymosin beta-10	P63313	Antimicrobial activity	[222]
Thymosin beta-4	P62328	Antimicrobial activity	[222]
Thyroxine-binding globulin	P05543	Protease inhibitor	[223]
Toll-interacting protein	Q9H0E2	Immunomodulatory effect	[224]
Transgelin-2	P37802	Immunomodulatory effect	[226]
Triokinase/FMN cyclase	Q3LXA3	Immunomodulatory effect	[230]
Tripeptidyl-peptidase 1	O14773	Serine protease activity	[231]
Tripeptidyl-peptidase 2	P29144	Serine protease activity	[232]
Trypsin-3	P35030	Serine protease activity	[234]
Uromodulin	P07911	Antimicrobial activity	[236,237]
Vitamin D-binding protein	P02774	Immunomodulatory effect	[239]
WAP four-disulfide core domain protein 2	Q14508	Protease inhibitor	[240]
WAP four-disulfide core domain protein 8	Q8IUA0	Protease inhibitor	[240]
Xaa-Pro aminopeptidase 1	Q9NQW7	Aminopeptidase activity	[241]

Xaa-Pro aminopeptidase 3	Q9NQH7	Aminopeptidase activity	[241]
Xaa-Pro dipeptidase	P12955	Carboxypeptidase activity	[242]
Zinc-alpha-2-glycoprotein	P25311	Immunomodulatory effect	[243]
Zymogen granule membrane protein 16	O60844	Antimicrobial activity	[244]
Zymogen granule protein 16 homolog B	Q96DA0	Antimicrobial activity	[245]

Table S9. Proteins involved in the first line of host defense in CSF.

Protein name	UniProt entry	Function	Reference
ADAM DEC1	O15204	Immunomodulatory effect	[2]
Alpha-1-acid glycoprotein 1	P02763	Immunomodulatory effect	[3]
Alpha-1-acid glycoprotein 2	P19652	Immunomodulatory effect	[3]
Alpha-1-antichymotrypsin	P01011	Protease inhibitor	[4]
Alpha-1-antitrypsin	P01009	Protease inhibitor	[5]
Alpha-1B-glycoprotein	P04217	Immunomodulatory effect	[6]
Alpha-2-antiplasmin	P08697	Protease inhibitor	[7]
Alpha-2-HS-glycoprotein	P02765	Anti-inflammatory effect	[8]
Alpha-2-macroglobulin	P01023	Protease inhibitor	[9]
Alpha-amylase 1A	P0DUB6	Regulation of biofilm formation	[11]
Alpha-amylase 1B	P0DTE7	Regulation of biofilm formation	[11]
Alpha-amylase 1C	P0DTE8	Regulation of biofilm formation	[11]
Alpha-amylase 2B	P19961	Regulation of biofilm formation	[11]
Aminopeptidase B	Q9H4A4	Exopeptidase activity	[12]
Aminopeptidase N	P15144	Exopeptidase activity	[13]
Amyloid-beta precursor protein	P05067	Antimicrobial activity	[15]
Angiogenin	P03950	Antimicrobial activity	[16]
Antileukoproteinase	P03973	Protease inhibitor<br>Immunomodulatory effect	[17,18]
Antithrombin-III	P01008	Protease inhibitor	[19]
Apolipoprotein A-I	P02647	Antimicrobial activity	[20]
Apolipoprotein A-II	P02652	Immunomodulatory effect	[21]
Apolipoprotein A-IV	P06727	Immunomodulatory effect	[22]
Apolipoprotein B-100	P04114	Antimicrobial activity	[23]
Apolipoprotein C-III	P02656	Immunomodulatory effect	[24]
Apolipoprotein D	P05090	Immunomodulatory effect	[26]
Apolipoprotein E	P02649	Immunomodulatory effect	[27]
Apolipoprotein L1	O14791	Immunomodulatory effect	[28]
Apolipoprotein M	O95445	Immunomodulatory effect	[29]
Arginase-1	P05089	Antifungal activity<br>Immunomodulatory effect	[30,31]
Aspartyl aminopeptidase	Q9ULA0	Exopeptidase activity	[32]

Azurocidin	P20160	Antimicrobial activity	[33]
Bactericidal permeability-increasing protein	P17213	Antimicrobial activity	[33,34]
Beta-2-glycoprotein 1	P02749	Immunomodulatory effect	[35]
Beta-2-microglobulin	P61769	Antimicrobial activity<br>Immunomodulatory effect	[36,37]
Beta-Ala-His dipeptidase	Q96KN2	Carboxypeptidase activity	[38]
Beta-defensin 1	P60022	Antimicrobial activity	[33]
Beta-defensin 103	P81534	Antimicrobial activity	[33]
Beta-hexosaminidase subunit alpha	P06865	Antimicrobial activity	[39]
Beta-hexosaminidase subunit beta	P07686	Antimicrobial activity	[39]
Bone marrow stromal antigen 2	Q10589	Antiviral effect	[40]
Calcitonin gene-related peptide 1	P06881	Antimicrobial activity	[48]
Calpain-1 catalytic subunit	P07384	Endopeptidase activity<br>Immunomodulatory effect	[49]
Calpain-2 catalytic subunit	P17655	Endopeptidase activity<br>Immunomodulatory effect	[49]
Calpain-5	O15484	Endopeptidase activity<br>Immunomodulatory effect	[49]
Calpastatin	P20810	Protease inhibitor	[50]
Carboxypeptidase A1	P15085	Carboxypeptidase activity	[51]
Carboxypeptidase A2	P48052	Carboxypeptidase activity	[51]
Carboxypeptidase A4	Q9UI42	Carboxypeptidase activity	[51]
Carboxypeptidase A5	Q8WXQ8	Carboxypeptidase activity	[51]
Carboxypeptidase B	P15086	Carboxypeptidase activity	[52]
Carboxypeptidase B2	Q96IY4	Carboxypeptidase activity	[52]
Carboxypeptidase D	O75976	Carboxypeptidase activity	[53]
Carboxypeptidase E	P16870	Carboxypeptidase activity	[53]
Carboxypeptidase M	P14384	Carboxypeptidase activity	[54]
Carboxypeptidase N catalytic chain	P15169	Carboxypeptidase activity	[55]
Carboxypeptidase Q	Q9Y646	Carboxypeptidase activity	[56]
Carboxypeptidase Z	Q66K79	Carboxypeptidase activity	[251]
Carcinoembryonic antigen-related cell adhesion molecule 1	P13688	Immunomodulatory effect	[57]
Catalase	P04040	Antimicrobial activity	[59]
Cathelicidin antimicrobial peptide	P49913	Antimicrobial activity	[33]
Cathepsin B	P07858	Endopeptidase activity	[60]
Cathepsin D	P07339	Endopeptidase activity	[60]
Cathepsin F	Q9UBX1	Endopeptidase activity	[60]
Cathepsin G	P08311	Endopeptidase activity	[60]
Cathepsin L2	O60911	Endopeptidase activity	[60]
Cathepsin O	P43234	Endopeptidase activity	[60]
Cathepsin S	P25774	Endopeptidase activity	[60]
Cathepsin Z	Q9UBR2	Endopeptidase activity	[60]
Cell surface glycoprotein MUC18	P43121	Immunomodulatory effect	[61]
Ceruloplasmin	P00450	Cu^{2+} sequestering activity	[62]

Chitinase-3-like protein 1	P36222	Antimicrobial activity	[63]
Chitotriosidase-1	Q13231	Antifungal activity	[64]
Chromogranin-A	P10645	Processed forms have antimicrobial activity	[65]
Clusterin	P10909	Immunomodulatory effect	[66]
Collagen alpha-1(XII) chain	Q99715	Immunomodulatory effect	[67]
Core histone macro-H2A.1	O75367	Antimicrobial activity	[68]
Corticosteroid-binding globulin	P08185	Protease inhibitor	[69]
C-reactive protein	P02741	Acute phase protein	[70]
Cystatin-A	P01040	Protease inhibitor	[71]
Cystatin-B	P04080	Protease inhibitor	[71]
Cystatin-C	P01034	Protease inhibitor	[71]
Cystatin-F	O76096	Protease inhibitor	[71]
Cystatin-M	Q15828	Protease inhibitor	[71]
Cystatin-S	P01036	Protease inhibitor	[71]
Cystatin-SA	P09228	Protease inhibitor	[71]
Cystatin-SN	P01037	Protease inhibitor	[71]
Cytosol aminopeptidase	P28838	Aminopeptidase activity	[72]
Cytosolic non-specific dipeptidase	Q96KP4	Carboxypeptidase activity	[75]
Deoxyribonuclease-1	P24855	Endonuclease activity	[78]
Dermcidin	P81605	Antimicrobial activity	[33]
Dipeptidase 2	Q9H4A9	Carboxypeptidase activity	[80]
Dipeptidase 3	Q9H4B8	Carboxypeptidase activity	[80]
Dipeptidyl peptidase 1	P53634	Carboxypeptidase activity	[81]
Dipeptidyl peptidase 2	Q9UHL4	Carboxypeptidase activity	[82]
Dipeptidyl peptidase 3	Q9NY33	Carboxypeptidase activity	[83]
Dipeptidyl peptidase 4	P27487	Carboxypeptidase activity	[84]
Dipeptidyl peptidase 9	Q86TI2	Carboxypeptidase activity	[85]
Disintegrin and metalloproteinase domain-containing protein 10	O14672	Metalloendopeptidase activity	[86]
Disintegrin and metalloproteinase domain-containing protein 11	O75078	Metalloendopeptidase activity	[86]
Disintegrin and metalloproteinase domain-containing protein 12	O43184	Metalloendopeptidase activity	[86]
Disintegrin and metalloproteinase domain-containing protein 15	Q13444	Metalloendopeptidase activity	[86]
Disintegrin and metalloproteinase domain-containing protein 17	P78536	Metalloendopeptidase activity	[86]
Disintegrin and metalloproteinase domain-containing protein 22	Q9P0K1	Metalloendopeptidase activity	[86]
Disintegrin and metalloproteinase domain-containing protein 23	O75077	Metalloendopeptidase activity	[86]
Disintegrin and metalloproteinase domain-containing protein 28	Q9UKQ2	Metalloendopeptidase activity	[86]

Disintegrin and metalloproteinase domain-containing protein 9	Q13443	Metalloendopeptidase activity	[86]
Drebrin-like protein	Q9UJU6	Immunomodulatory effect	[87]
Elafin	P19957	Protease inhibitor	[88]
Endoplasmic reticulum aminopeptidase 1	Q9NZ08	Aminopeptidase activity	[89]
Endoplasmic reticulum aminopeptidase 2	Q6P179	Aminopeptidase activity	[89]
Eosinophil cationic protein	P12724	Antimicrobial activity	[90]
Extracellular glycoprotein lacritin	Q9GZZ8	Antimicrobial activity	[92]
Fatty acid-binding protein 4	P15090	Immunomodulatory effect	[93]
Fatty acid-binding protein 5	Q01469	Immunomodulatory effect	[94]
Fibrinogen alpha chain	P02671	Immunomodulatory effect	[95]
Fibrinogen beta chain	P02675	Immunomodulatory effect	[95]
Fibrinogen gamma chain	P02679	Immunomodulatory effect	[95]
Fibroleukin	Q14314	Immunomodulatory effect	[97]
Folliculin-interacting protein 1	Q8TF40	Immunomodulatory effect	[98]
Furin	P09958	Serine protease activity	[99]
Galectin-1	P09382	Immunomodulatory effect	[101]
Galectin-3	P17931	Immunomodulatory effect	[103]
Galectin-3-binding protein	Q08380	Antimicrobial activity<br>Immunomodulatory effect	[104,105]
Galectin-7	P47929	Immunomodulatory effect	[106]
Gelsolin	P06396	Processed from has antimicrobial activity	[109]
Glia-derived nexin	P07093	Protease inhibitor	[110]
Glucose-6-phosphate isomerase	P06744	Induces immunoglobulin secretion	[111]
Glutamate carboxypeptidase 2	Q04609	Carboxypeptidase activity	[112]
Glutamyl aminopeptidase	Q07075	Aminopeptidase activity	[113]
Glutathione S-transferase omega-1	P78417	Immunomodulatory effect	[114]
Glutathione S-transferase P	P09211	Immunomodulatory effect	[115]
Glyceraldehyde-3-phosphate dehydrogenase	P04406	Immunomodulatory effect	[116]
Growth-regulated alpha protein	P09341	Antimicrobial activity	[118]
Guanylate-binding protein 1	P32455	Immunomodulatory effect	[119]
Haptoglobin	P00738	Immunomodulatory effect<br>Iron sequestrating	[122]
Haptoglobin-related protein	P00739	Anti-parasitic effect	[123]
Heme-binding protein 1	Q9NRV9	Heme/iron sequestration	[124]
Heme-binding protein 2	Q9Y5Z4	Heme/iron sequestration	[124]
Hemoglobin subunit alpha	P69905	Processed forms (hemocidins) have antimicrobial activity	[125]
Hemoglobin subunit beta	P68871	Processed forms (hemocidins) have antimicrobial activity	[125]
Hemopexin	P02790	Antibacterail effect	[126]

Heparin cofactor 2	P05546	Anti-inflammatory effect<br>Protease inhibitor	[127]
Hepcidin	P81172	Antimicrobial activity<br>Iron sequestration	[128,129]
High mobility group protein B1	P09429	Immunomodulatory effect	[130]
High mobility group protein B2	P26583	Antimicrobial activity	[131]
High mobility group protein B3	O15347	Immunomodulatory effect	[132]
Histidine-rich glycoprotein	P04196	Antimicrobial activity	[133]
Histone H1.0	P07305	Antimicrobial activity	[134]
Histone H1.1	Q02539	Antimicrobial activity	[134]
Histone H1.10	Q92522	Antimicrobial activity	[134]
Histone H1.2	P16403	Antimicrobial activity	[134]
Histone H1.3	P16402	Antimicrobial activity	[134]
Histone H1.4	P10412	Antimicrobial activity	[134]
Histone H1.5	P16401	Antimicrobial activity	[134]
Histone H2A type 1	P0C0S8	Antimicrobial activity	[134]
Histone H2A type 1-B/E	P04908	Antimicrobial activity	[134]
Histone H2A type 1-C	Q93077	Antimicrobial activity	[134]
Histone H2A type 1-D	P20671	Antimicrobial activity	[134]
Histone H2A type 1-H	Q96KK5	Antimicrobial activity	[134]
Histone H2A type 1-J	Q99878	Antimicrobial activity	[134]
Histone H2A type 2-A	Q6FI13	Antimicrobial activity	[134]
Histone H2A type 2-B	Q8IUE6	Antimicrobial activity	[134]
Histone H2A type 2-C	Q16777	Antimicrobial activity	[134]
Histone H2A type 3	Q7L7L0	Antimicrobial activity	[134]
Histone H2A.J	Q9BTM1	Antimicrobial activity	[134]
Histone H2A.V	Q71UI9	Antimicrobial activity	[134]
Histone H2A.Z	P0C0S5	Antimicrobial activity	[134]
Histone H2AX	P16104	Antimicrobial activity	[134]
Histone H2B type 1-A	Q96A08	Antimicrobial activity	[134]
Histone H2B type 1-B	P33778	Antimicrobial activity	[134]
Histone H2B type 1-C/E/F/G/I	P62807	Antimicrobial activity	[134]
Histone H2B type 1-D	P58876	Antimicrobial activity	[134]
Histone H2B type 1-H	Q93079	Antimicrobial activity	[134]
Histone H2B type 1-J	P06899	Antimicrobial activity	[134]
Histone H2B type 1-K	O60814	Antimicrobial activity	[134]
Histone H2B type 1-L	Q99880	Antimicrobial activity	[134]
Histone H2B type 1-M	Q99879	Antimicrobial activity	[134]
Histone H2B type 1-N	Q99877	Antimicrobial activity	[134]
Histone H2B type 1-O	P23527	Antimicrobial activity	[134]
Histone H2B type 2-E	Q16778	Antimicrobial activity	[134]
Histone H2B type 2-F	Q5QNW6	Antimicrobial activity	[134]
Histone H2B type 3-B	Q8N257	Antimicrobial activity	[134]
Histone H2B type F-S	P57053	Antimicrobial activity	[134]

Histone H3.1	P68431	Antimicrobial activity	[134]
Histone H3.1t	Q16695	Antimicrobial activity	[134]
Histone H3.2	Q71DI3	Antimicrobial activity	[134]
Histone H3.3	P84243	Antimicrobial activity	[134]
Histone H4	P62805	Antimicrobial activity	[134]
Inter-alpha-trypsin inhibitor heavy chain H1	P19827	Protease inhibitor	[135]
Inter-alpha-trypsin inhibitor heavy chain H2	P19823	Protease inhibitor	[135]
Inter-alpha-trypsin inhibitor heavy chain H3	Q06033	Protease inhibitor	[135]
Inter-alpha-trypsin inhibitor heavy chain H4	Q14624	Protease inhibitor	[135]
Inter-alpha-trypsin inhibitor heavy chain H5	Q86UX2	Protease inhibitor	[135]
Kallikrein-10	O43240	Serine protease activity	[138]
Kallikrein-11	Q9UBX7	Serine protease activity	[138]
Kallikrein-13	Q9UKR3	Serine protease activity	[138]
Kallikrein-3	P07288	Serine protease activity	[138]
Kallikrein-6	Q92876	Serine protease activity	[138]
Kallikrein-7	P49862	Serine protease activity<br>Processing the maturation of LL-37 cathelicidin	[138]
Kallikrein-8	O60259	Serine protease activity	[138]
Kininogen-1	P01042	Antimicrobial activity	[140,141]
Kunitz-type protease inhibitor 1	O43278	Protease inhibitor	[142]
Kunitz-type protease inhibitor 2	O43291	Protease inhibitor	[142]
Lactotransferrin	P02788	Antimicrobial activity<br>Iron sequestration	[33]
Legumain	Q99538	Endopeptidase activity	[144]
Leukocyte elastase inhibitor	P30740	Protease inhibitor	[145]
Lipocalin-1	P31025	Immunomodulatory effect<br>Iron sequestration	[146,147]
Lipocalin-2	P80188	Immunomodulatory effect<br>Iron sequestration	[146,147]
Lipopolysaccharide-binding protein	P18428	Immunomodulatory effect	[148]
Liver-expressed antimicrobial peptide 2	Q969E1	Antimicrobial activity	[149]
Lymphotactin	P47992	Antimicrobial activity	[150]
Lysozyme C	P61626	Antimicrobial activity	[33]
Macrophage migration inhibitory factor	P14174	Antimicrobial activity	[151]
Mammaglobin-B	O75556	Immunomodulatory effect	[153]
Matrix metalloproteinase-9	P14780	Metalloprotease activity	[155]
Melanotransferrin	P08582	Iron sequestration	[156]
Metalloproteinase inhibitor 1	P01033	Protease Inhibitor	[157]
Metalloproteinase inhibitor 2	P16035	Protease Inhibitor	[157]
Metalloproteinase inhibitor 3	P35625	Protease Inhibitor	[157]
Metalloproteinase inhibitor 4	Q99727	Protease Inhibitor	[157]
Midkine	P21741	Immunomodulatory effect	[158]
Moesin	P26038	Immunomodulatory effect	[159]

Mucin-16	Q8WXI7	Antimicrobial activity	[160]
Mucin-3A	Q02505	Antimicrobial activity	[160]
Myeloperoxidase	P05164	Antimicrobial activity	[162]
Myoglobin	P02144	Processed forms (hemo-cidins) have antimicrobial activity	[163]
N-acetylmuramoyl-L-alanine amidase	Q96PD5	Antimicrobial activity	[164]
Neprilysin	P08473	Endopeptidase activity	[165]
Neutrophil collagenase	P22894	Endopeptidase activity<br>Immunomodulatory effect	[166]
Neutrophil defensin 1	P59665	Antimicrobial activity	[33]
Neutrophil defensin 3	P59666	Antimicrobial activity	[33]
Neutrophil elastase	P08246	Serine protease activity	[167]
Nicotinamide phosphoribosyltransferase	P43490	Immunomodulatory effect	[168]
Non-histone chromosomal protein HMG-17	P05204	Antimicrobial activity	[169]
Non-secretory ribonuclease	P10153	Ribonuclease activity	[170]
Peptidase inhibitor 16	Q6UXB8	Protease inhibitor	[172]
Peptidoglycan recognition protein 1	O75594	Antimicrobial activity	[173]
Phospholipase B-like 1	Q6P4A8	Suggested antimicrobial activity	[175]
Pigment epithelium-derived factor	P36955	Protease inhibitor	[176]
Plasma kallikrein	P03952	Serine protease activity	[138]
Plasma serine protease inhibitor	P05154	Protease inhibitor	[177]
Plastin-2	P13796	Immunomodulatory effect	[178]
Poly(rC)-binding protein 1	Q15365	Antiviral effect	[179]
Poly(rC)-binding protein 2	Q15366	Antiviral effect	[180]
Pregnancy zone protein	P20742	Protease inhibitor	[181]
Pro-adrenomedullin	P35318	Antimicrobial activity<br>Immunomodulatory effect	[182,183]
Pro-cathepsin H	P09668	Endopeptidase activity	[60]
Procathepsin L	P07711	Endopeptidase activity	[60]
Progranulin	P28799	Immunomodulatory effect	[184]
Prolactin-inducible protein	P12273	Aspartic-type endopepti-dase activity<br>Modulates the activity of Zn-α2 glycoprotein	[185,186]
Proline-rich protein 14	Q9BWN1	Antimicrobial activity	[187]
Proline-rich protein 15	Q8IV56	Antimicrobial activity	[187]
Proline-rich protein 20A	P86496	Antimicrobial activity	[187]
Proline-rich protein 20B	P86481	Antimicrobial activity	[187]
Proline-rich protein 20C	P86479	Antimicrobial activity	[187]
Proline-rich protein 20D	P86480	Antimicrobial activity	[187]
Proline-rich protein 20E	P86478	Antimicrobial activity	[187]
Prolyl endopeptidase	P48147	Endopeptidase activity	[188]
Pro-opiomelanocortin	P01189	Antimicrobial activity	[189]
Prosaposin	P07602	Processed forms has antimi-	[191]

		crobial effect	
Prostasin	Q16651	Serine protease activity	[192]
Protachykinin-1	P20366	Antimicrobial activity	[254]
Protein AMBP	P02760	Protease inhibitor	[193]
Protein FAM3A	P98173	Antifungal effect	[194]
Protein S100-A1	P23297	Immunomodulatory effect	[195]
Protein S100-A10	P60903	Immunomodulatory effect	[196]
Protein S100-A11	P31949	Immunomodulatory effect	[197]
Protein S100-A12	P80511	Immunomodulatory effect	[196]
Protein S100-A13	Q99584	Immunomodulatory effect	[198]
Protein S100-A14	Q9HCY8	Immunomodulatory effect	[199]
Protein S100-A4	P26447	Immunomodulatory effect	[196]
Protein S100-A6	P06703	Immunomodulatory effect	[201]
Protein S100-A7	P31151	Immunomodulatory effect	[196]
Protein S100-A8	P05109	Immunomodulatory effect	[196]
Protein S100-A9	P06702	Immunomodulatory effect	[196]
Protein S100-B	P04271	Immunomodulatory effect	[196]
Protein S100-P	P25815	Immunomodulatory effect	[196]
Puromycin-sensitive aminopeptidase	P55786	Aminopeptidase activity	[203]
Retroviral-like aspartic protease 1	Q53RT3	Aspartic-type endopeptidase activity	[205]
Ribonuclease 4	P34096	Ribonuclease activity	[170]
Ribonuclease 7	Q9H1E1	Ribonuclease activity	[170]
Ribonuclease K6	Q93091	Ribonuclease activity	[170]
Ribonuclease pancreatic	P07998	Ribonuclease activity	[170]
Ribonuclease T2	O00584	Ribonuclease activity	[170]
Secreted Ly-6/uPAR domain-containing protein 2	P0DP57	Immunomodulatory effect	[247]
Secreted Ly-6/uPAR-related protein 1	P55000	Immunomodulatory effect	[206]
Semenogelin-1	P04279	Processed forms has antimicrobial activity	[209–211]
Serine protease 1	P07477	Serine protease activity	[212]
Serine protease 23	O95084	Serine protease activity	[212]
Serine protease 27	Q9BQR3	Serine protease activity	[212]
Serine protease HTRA1	Q92743	Serine protease activity	[212]
Serine protease HTRA2	O43464	Serine protease activity	[212]
Serine protease HTRA3	P83110	Serine protease activity	[212]
Serine protease inhibitor Kazal-type 1	P00995	Protease inhibitor	[213]
Serine protease inhibitor Kazal-type 2	P20155	Protease inhibitor	[213]
Serine protease inhibitor Kazal-type 5	Q9NQ38	Protease inhibitor	[213]
Serine protease inhibitor Kazal-type 6	Q6UWN8	Protease inhibitor	[213]
Serine protease inhibitor Kazal-type 7	P58062	Protease inhibitor	[213]
Serotransferrin	P02787	Iron sequestration	[214]
Serpin B10	P48595	Protease inhibitor	[215]

Serpin B11	Q96P15	Protease inhibitor	[215]
Serpin B3	P29508	Protease inhibitor	[215]
Serpin B4	P48594	Protease inhibitor	[215]
Serpin B6	P35237	Protease inhibitor	[215]
Serpin B8	P50452	Protease inhibitor	[215]
Serpin B9	P50453	Protease inhibitor	[215]
Serum amyloid A-1 protein	P0DJI8	Immunomodulatory effect	[216]
Serum amyloid A-2 protein	P0DJI9	Immunomodulatory effect	[216]
Serum amyloid A-4 protein	P35542	Immunomodulatory effect	[216]
Serum amyloid P-component	P02743	Antiviral effect	[217]
Small proline-rich protein 3	Q9UBC9	Antimicrobial activity	[187]
Sperm-associated antigen 11B	Q08648	Antimicrobial activity	[255]
Syntenin-1	O00560	Immunomodulatory effect	[219]
T-cell immunomodulatory protein	Q8TB96	Immunomodulatory effect	[220]
Thioredoxin domain-containing protein 17	Q9BRA2	Immunomodulatory effect	[221]
Thymosin beta-10	P63313	Antimicrobial activity	[222]
Thymosin beta-4	P62328	Antimicrobial activity	[222]
Thyroxine-binding globulin	P05543	Protease inhibitor	[223]
Toll-interacting protein	Q9H0E2	Immunomodulatory effect	[224]
Transgelin	Q01995	Immunomodulatory effect	[225]
Transgelin-2	P37802	Immunomodulatory effect	[226]
Triokinase/FMN cyclase	Q3LXA3	Immunomodulatory effect	[230]
Tripeptidyl-peptidase 1	O14773	Serine protease activity	[231]
Trypsin-2	P07478	Serine protease activity	[233]
Trypsin-3	P35030	Serine protease activity	[234]
Tryptase alpha/beta-1	Q15661	Serine protease activity	[234]
Tryptase beta-2	P20231	Serine protease activity	[234]
Uromodulin	P07911	Antimicrobial activity	[236,237]
Uteroglobin	P11684	Immunomodulatory effect	[238]
Vitamin D-binding protein	P02774	Immunomodulatory effect	[239]
WAP four-disulfide core domain protein 1	Q9HC57	Protease inhibitor	[240]
WAP four-disulfide core domain protein 2	Q14508	Protease inhibitor	[240]
WAP, Kazal, immunoglobulin, Kunitz and NTR domain-containing protein 2	Q8TEU8	Protease inhibitor	[240]
Xaa-Pro aminopeptidase 1	Q9NQW7	Aminopeptidase activity	[241]
Xaa-Pro dipeptidase	P12955	Carboxypeptidase activity	[242]
Zinc-alpha-2-glycoprotein	P25311	Immunomodulatory effect	[243]
Zymogen granule membrane protein 16	O60844	Antimicrobial activity	[244]
Zymogen granule protein 16 homolog B	Q96DA0	Antimicrobial activity	[245]

References

1. Moreno, R.D.; Laserre, A.A.; Barros, C. Protease activity involvement in the passage of mammalian sperm through the zona pellucida. *Biol. Res.* **2011**, *44*, 145–150, doi:10.4067/S0716-97602011000200006.

2. Kumagai, T.; Fan, S.; Smith, A.M. <p>ADAMDEC1 and Its Role in Inflammatory Disease and Cancer</p>. *Met. Med.* **2020**, *7*, 15–28, doi:10.2147/MNM.S263813.

3. Ceciliani, F.; Lecchi, C. The Immune Functions of α 1 Acid Glycoprotein. *Curr. Protein Pept. Sci.* **2019**, *20*, 505–524, doi:10.2174/1389203720666190405101138.

4. Dimberg, J.; Ström, K.; Löfgren, S.; Zar, N.; Hugander, A.; Matussek, A. Expression of the serine protease inhibitor serpinA3 in human colorectal adenocarcinomas. *Oncol. Lett.* **2011**, *2*, 413, doi:10.3892/OL.2011.280.

5. Janciauskiene, S.; Wrenger, S.; Immenschuh, S.; Olejnicka, B.; Greulich, T.; Welte, T.; Chorostowska-Wynimko, J. The multifaceted effects of Alpha1-Antitrypsin on neutrophil functions. *Front. Pharmacol.* **2018**, *9*, 341, doi:10.3389/FPHAR.2018.00341/BIBTEX.

6. Cederfur, C.; Salomonsson, E.; Nilsson, J.; Halim, A.; Öberg, C.T.; Larson, G.; Nilsson, U.J.; Leffler, H. Different affinity of galectins for human serum glycoproteins: galectin-3 binds many protease inhibitors and acute phase proteins. *Glycobiology* **2008**, *18*, 384–394, doi:10.1093/GLYCOB/CWN015.

7. Singh, S.; Saleem, S.; Reed, G.L. Alpha2-Antiplasmin: The Devil You Don't Know in Cerebrovascular and Cardiovascular Disease. *Front. Cardiovasc. Med.* **2020**, *7*, 363, doi:10.3389/FCVM.2020.608899/BIBTEX.

8. Wang, H.; E. Sama, A. Anti-inflammatory role of fetuin-A in injury and infection. *Curr. Mol. Med.* **2012**, *12*, 625–633, doi:10.2174/156652412800620039.

9. Vandooren, J.; Itoh, Y. Alpha-2-Macroglobulin in Inflammation, Immunity and Infections. *Front. Immunol.* **2021**, *12*, 5411, doi:10.3389/FIMMU.2021.803244/BIBTEX.

10. Harwood, S.L.; Nielsen, N.S.; Jensen, K.T.; Nielsen, P.K.; Thøgersen, I.B.; Enghild, J.J. α 2-Macroglobulin-like protein 1 can conjugate and inhibit proteases through their hydroxyl groups, because of an enhanced reactivity of its thiol ester. *J. Biol. Chem.* **2020**, *295*, 16732–16742, doi:10.1074/JBC.RA120.015694.

11. Lahiri, D.; Nag, M.; Banerjee, R.; Mukherjee, D.; Garai, S.; Sarkar, T.; Dey, A.; Sheikh, H.I.; Pathak, S.K.; Edinur, H.A.; et al. Amylases: Biofilm Inducer or Biofilm Inhibitor? *Front. Cell. Infect. Microbiol.* **2021**, *11*, 355, doi:10.3389/FCIMB.2021.660048/BIBTEX.

12. Cadel, S.; Piesse, C.; Pham, V.L.; Pernier, J.; Hanquez, C.; Gouzy-Darmon, C.A.; Foulon, T. Aminopeptidase B. *Handb. Proteolytic Enzym.* **2013**, *1*, 473–479, doi:10.1016/B978-0-12-382219-2.00097-1.

13. Turner, A.J. Aminopeptidase N. *Handb. Proteolytic Enzym.* **2013**, *1*, 397, doi:10.1016/B978-0-12-382219-2.00079-X.

14. Díaz-Perales, A.; Quesada, V.; Sánchez, L.M.; Ugalde, A.P.; Suárez, M.F.; Fueyo, A.; López-Otín, C. Identification of Human Aminopeptidase O, a Novel Metalloprotease with Structural Similarity to Aminopeptidase B and Leukotriene A4 Hydrolase *. *J. Biol. Chem.* **2005**, *280*, 14310–14317, doi:10.1074/JBC.M413222200.

15. Soscia, S.J.; Kirby, J.E.; Washicosky, K.J.; Tucker, S.M.; Ingelsson, M.; Hyman, B.; Burton, M.A.; Goldstein, L.E.; Duong, S.; Tanzi, R.E.; et al. The Alzheimer's Disease-Associated Amyloid β-Protein Is an Antimicrobial Peptide. *PLoS One* **2010**, *5*, e9505, doi:10.1371/JOURNAL.PONE.0009505.

16. Hooper, L. V.; Stappenbeck, T.S.; Hong, C. V.; Gordon, J.I. Angiogenins: a new class of microbicidal proteins involved in innate immunity. *Nat. Immunol. 2003 43* **2003**, *4*, 269–273, doi:10.1038/ni888.

17. Mulligan, M.S.; Lentsch, A.B.; Huber-Lang, M.; Guo, R.F.; Sarma, V.; Wright, C.D.; Ulich, T.R.; Ward, P.A. Anti-inflammatory effects of mutant forms of secretory leukocyte protease inhibitor. *Am. J. Pathol.* **2000**, *156*, 1033–1039, doi:10.1016/S0002-9440(10)64971-1.

18. Vandooren, J.; Goeminne, P.; Boon, L.; Ugarte-Berzal, E.; Rybakin, V.; Proost, P.; Abu El-Asrar, A.M.; Opdenakker, G. Neutrophils and activated macrophages control mucosal immunity by proteolytic cleavage of antileukoproteinase. *Front. Immunol.* **2018**, *9*, 28, doi:10.3389/FIMMU.2018.01154/FULL.

19. Roemisch, J.; Gray, E.; Hoffmann, J.N.; Wiedermann, C.J.; Kalina, U. Antithrombin: a new look at the actions of a serine protease inhibitor. *Blood Coagul. Fibrinolysis* **2002**, *13*, 657–670, doi:10.1097/00001721-200212000-00001.

20. Tada, N.; Sakamoto, T.; Kagami, A.; Mochizuki, K.; Kurosaka, K. Antimicrobial activity of lipoprotein particles containing

apolipoprotein Al. *Mol. Cell. Biochem.* **1993**, *119*, 171–178, doi:10.1007/BF00926868.
21. Thompson, P.A.; Berbée, J.F.P.; Rensen, P.C.N.; Kitchens, R.L. Apolipoprotein A-II augments monocyte responses to LPS by suppressing the inhibitory activity of LPS-binding protein. *Innate Immun.* **2008**, *14*, 365–374, doi:10.1177/1753425908099171.
22. Recalde, D.; Ostos, M.A.; Badell, E.; Garcia-Otin, A.L.; Pidoux, J.; Castro, G.; Zakin, M.M.; Scott-Algara, D. Human apolipoprotein A-IV reduces secretion of proinflammatory cytokines and atherosclerotic effects of a chronic infection mimicked by lipopolysaccharide. *Arterioscler. Thromb. Vasc. Biol.* **2004**, *24*, 756–761, doi:10.1161/01.ATV.0000119353.03690.22.
23. Gaglione, R.; Cesaro, A.; Dell'Olmo, E.; Della Ventura, B.; Casillo, A.; Di Girolamo, R.; Velotta, R.; Notomista, E.; Veldhuizen, E.J.A.; Corsaro, M.M.; et al. Effects of human antimicrobial cryptides identified in apolipoprotein B depend on specific features of bacterial strains. *Sci. Reports 2019 91* **2019**, *9*, 1–13, doi:10.1038/s41598-019-43063-3.
24. Zewinger, S.; Reiser, J.; Jankowski, V.; Alansary, D.; Hahm, E.; Triem, S.; Klug, M.; Schunk, S.J.; Schmit, D.; Kramann, R.; et al. Apolipoprotein C3 induces inflammation and organ damage by alternative inflammasome activation. *Nat. Immunol. 2019 211* **2019**, *21*, 30–41, doi:10.1038/s41590-019-0548-1.
25. Mak, P.A.; Laffitte, B.A.; Desrumaux, C.; Joseph, S.B.; Curtiss, L.K.; Mangelsdorf, D.J.; Tontonoz, P.; Edwards, P.A. Regulated expression of the apolipoprotein E/C-I/C-IV/C-II gene cluster in murine and human macrophages. A critical role for nuclear liver X receptors alpha and beta. *J. Biol. Chem.* **2002**, *277*, 31900–31908, doi:10.1074/JBC.M202993200.
26. Crespo-Sanjuán, J.; Zamora-Gonzalez, N.; DoloresCalvo-Nieves, M.; Andres-Ledesma, C. Apolipoprotein D. *Adv. Lipoprotein Res.* **2017**, doi:10.5772/66626.
27. Zhang, H.; Wu, L.M.; Wu, J. Cross-talk between apolipoprotein E and cytokines. *Mediators Inflamm.* **2011**, *2011*, doi:10.1155/2011/949072.
28. Fang, J.; Yao, X.; Hou, M.; Duan, M.; Xing, L.; Huang, J.; Wang, Y.; Zhu, B.; Chen, Q.; Wang, H. ApoL1 induces kidney inflammation through RIG-I/NF-κB activation. *Biochem. Biophys. Res. Commun.* **2020**, *527*, 466–473, doi:10.1016/J.BBRC.2020.04.054.
29. Wang, M.; Luo, G.H.; Liu, H.; Zhang, Y.P.; Wang, B.; Di, D.M.; Zhan, X.H.; Yu, Y.; Yao, S.; Zhang, X.Y.; et al. Apolipoprotein M induces inhibition of inflammatory responses via the S1PR1 and DHCR24 pathways. *Mol. Med. Rep.* **2019**, *19*, 1272–1283, doi:10.3892/MMR.2018.9747.
30. Oberlies, J.; Watzl, C.; Giese, T.; Luckner, C.; Kropf, P.; Müller, I.; Ho, A.D.; Munder, M. Regulation of NK Cell Function by Human Granulocyte Arginase. *J. Immunol.* **2009**, *182*, 5259–5267, doi:10.4049/JIMMUNOL.0803523.
31. Munder, M.; Mollinedo, F.; Calafat, J.; Canchado, J.; Gil-Lamaignere, C.; Fuentes, J.M.; Luckner, C.; Doschko, G.; Soler, G.; Eichmann, K.; et al. Arginase I is constitutively expressed in human granulocytes and participates in fungicidal activity. *Blood* **2005**, *105*, 2549–2556, doi:10.1182/BLOOD-2004-07-2521.
32. Chaikuad, A.; Pilka, E.S.; De Riso, A.; Von Delft, F.; Kavanagh, K.L.; Vénien-Bryan, C.; Oppermann, U.; Yue, W.W. Structure of human aspartyl aminopeptidase complexed with substrate analogue: insight into catalytic mechanism, substrate specificity and M18 peptidase family. *BMC Struct. Biol.* **2012**, *12*, 14, doi:10.1186/1472-6807-12-14.
33. Wiesner, J.; Vilcinskas, A. Antimicrobial peptides: the ancient arm of the human immune system. *Virulence* **2010**, *1*, 440–464, doi:10.4161/viru.1.5.12983.
34. Canny, G.; Levy, O. Bactericidal/permeability-increasing protein (BPI) and BPI homologs at mucosal sites. *Trends Immunol.* **2008**, *29*, 541–547, doi:10.1016/J.IT.2008.07.012.
35. Serrano, M.; Morán, L.; Martinez-Flores, J.A.; Mancebo, E.; Pleguezuelo, D.; Cabrera-Marante, O.; Delgado, J.; Serrano, A. Immune Complexes of Beta-2-Glycoprotein I and IgA Antiphospholipid Antibodies Identify Patients With Elevated Risk of Thrombosis and Early Mortality After Heart Transplantation. *Front. Immunol.* **2019**, *10*, 2891, doi:10.3389/FIMMU.2019.02891/BIBTEX.
36. Xie, J.; Yi, Q.; Uchanska-Ziegler, B.; Ziegler, A. β2-microglobulin as a potential initiator of inflammatory responses. *Trends Immunol.* **2003**, *24*, 228–229, doi:10.1016/S1471-4906(03)00076-0.
37. Chiou, S.J.; Ko, H.J.; Hwang, C.C.; Hong, Y.R. The Double-Edged Sword of Beta2-Microglobulin in Antibacterial Properties

and Amyloid Fibril-Mediated Cytotoxicity. *Int. J. Mol. Sci. 2021, Vol. 22, Page 6330* **2021**, *22*, 6330, doi:10.3390/IJMS22126330.

38. Veiga-da-Cunha, M.; Chevalier, N.; Stroobant, V.; Vertommen, D.; Van Schaftingen, E. Metabolite Proofreading in Carnosine and Homocarnosine Synthesis: MOLECULAR IDENTIFICATION OF PM20D2 AS β-ALANYL-LYSINE DIPEPTIDASE*. *J. Biol. Chem.* **2014**, *289*, 19726, doi:10.1074/JBC.M114.576579.
39. Koo, I.C.; Ohol, Y.M.; Wu, P.; Morisaki, J.H.; Cox, J.S.; Brown, E.J. Role for lysosomal enzyme β-hexosaminidase in the control of mycobacteria infection. *Proc. Natl. Acad. Sci. U. S. A.* **2008**, *105*, 710, doi:10.1073/PNAS.0708110105.
40. Jouvenet, N.; Neil, S.J.D.; Zhadina, M.; Zang, T.; Kratovac, Z.; Lee, Y.; McNatt, M.; Hatziioannou, T.; Bieniasz, P.D. Broad-Spectrum Inhibition of Retroviral and Filoviral Particle Release by Tetherin. *J. Virol.* **2009**, *83*, 1837–1844, doi:10.1128/JVI.02211-08/ASSET/0B2B503A-4881-4944-AD19-C39D684B829A/ASSETS/GRAPHIC/ZJV0040915210006.JPEG.
41. Liu, Y.; Bartlett, J.A.; Di, M.E.; Bomberger, J.M.; Chan, Y.R.; Gakhar, L.; Mallampalli, R.K.; McCray, P.B.; Di, Y.P. SPLUNC1/BPIFA1 Contributes to Pulmonary Host Defense against Klebsiella pneumoniae Respiratory Infection. *Am. J. Pathol.* **2013**, *182*, 1519–1531, doi:10.1016/J.AJPATH.2013.01.050.
42. Prokopovic, V.; Popovic, M.; Andjelkovic, U.; Marsavelski, A.; Raskovic, B.; Gavrovic-Jankulovic, M.; Polovic, N. Isolation, biochemical characterization and anti-bacterial activity of BPIFA2 protein. *Arch. Oral Biol.* **2014**, *59*, 302–309, doi:10.1016/J.ARCHORALBIO.2013.12.005.
43. Ebersole, J.L.; Kirakodu, S.; Nguyen, L.; Gonzalez, O.A. Gingival Transcriptome of Innate Antimicrobial Factors and the Oral Microbiome With Aging and Periodontitis. *Front. oral Heal.* **2022**, *3*, doi:10.3389/FROH.2022.817249.
44. Huang, Y.; Wang, M.; Hong, Y.; Bu, X.; Luan, G.; Wang, Y.; Li, Y.; Lou, H.; Wang, C.; Zhang, L. Reduced Expression of Antimicrobial Protein Secretory Leukoprotease Inhibitor and Clusterin in Chronic Rhinosinusitis with Nasal Polyps. *J. Immunol. Res.* **2021**, *2021*, doi:10.1155/2021/1057186.
45. Delorme-Axford, E.; Morosky, S.; Bomberger, J.; Stolz, D.B.; Jackson, W.T.; Coyne, C.B. BPIFB3 Regulates Autophagy and Coxsackievirus B Replication through a Noncanonical Pathway Independent of the Core Initiation Machinery. *MBio* **2014**, *5*, doi:10.1128/MBIO.02147-14/SUPPL_FILE/MBO006142080SF7.TIF.
46. Ciaglia, E.; Montella, F.; Lopardo, V.; Scala, P.; Ferrario, A.; Cattaneo, M.; Carrizzo, A.; Malovini, A.; Madeddu, P.; Vecchione, C.; et al. Circulating BPIFB4 Levels Associate With and Influence the Abundance of Reparative Monocytes and Macrophages in Long Living Individuals. *Front. Immunol.* **2020**, *11*, 1034, doi:10.3389/FIMMU.2020.01034/BIBTEX.
47. Wong, G.W.; Yasuda, S.; Madhusudhan, M.S.; Li, L.; Yang, Y.; Krilis, S.A.; Šali, A.; Stevens, R.L. Human tryptase epsilon (PRSS22), a new member of the chromosome 16p13.3 family of human serine proteases expressed in airway epithelial cells. *J. Biol. Chem.* **2001**, *276*, 49169–49182, doi:10.1074/JBC.M108677200.
48. El Karim, I.A.; Linden, G.J.; Orr, D.F.; Lundy, F.T. Antimicrobial activity of neuropeptides against a range of micro-organisms from skin, oral, respiratory and gastrointestinal tract sites. *J. Neuroimmunol.* **2008**, *200*, 11–16, doi:10.1016/J.JNEUROIM.2008.05.014.
49. Ji, J.; Su, L.; Liu, Z. Critical role of calpain in inflammation. *Biomed. Reports* **2016**, *5*, 647–652, doi:10.3892/BR.2016.785/HTML.
50. Luo, Y.; Sellitti, D.F.; Suzuki, K. The Calpain Proteolytic System. *Encycl. Cell Biol.* **2016**, *1*, 670–680, doi:10.1016/B978-0-12-394447-4.10075-6.
51. Morrison, H. Carboxypeptidase A. *Enzym. Act. Sites their React. Mech.* **2021**, 37–40, doi:10.1016/B978-0-12-821067-3.00008-8.
52. Avilés, F.X.; Vendrell, J. Carboxypeptidase B. *Handb. Proteolytic Enzym.* **2013**, *1*, 1324–1329, doi:10.1016/B978-0-12-382219-2.00297-0.
53. Fricker, L.D. Carboxypeptidases E and D. *Handb. Biol. Act. Pept.* **2013**, 1715–1720, doi:10.1016/B978-0-12-385095-9.00235-9.
54. Skidgel, R.A. Carboxypeptidase M. *Handb. Proteolytic Enzym. Second Ed.* **2004**, *1*, 851–854, doi:10.1016/B978-0-12-079611-3.50260-3.
55. Matthews, K.W.; Mueller-Ortiz, S.L.; Wetsel, R.A. Carboxypeptidase N: a pleiotropic regulator of inflammation. *Mol. Immunol.* **2004**, *40*, 785–793, doi:10.1016/J.MOLIMM.2003.10.002.
56. Lee, J.-H.; Cho, H.-S.; Lee, J.-J.; Jun, S.Y.; Ahn, J.-H.; Min, J.-S.; Yoon, J.-Y.; Choi, M.-H.; Jeon, S.-J.; Lim, J.H.; et al. Plasma

glutamate carboxypeptidase is a negative regulator in liver cancer metastasis. *Oncotarget* **2016**, *7*, 79774–79786, doi:10.18632/ONCOTARGET.12967.

57. Hosomi, S.; Chen, Z.; Baker, K.; Chen, L.; Huang, Y.H.; Olszak, T.; Zeissig, S.; Wang, J.H.; Mandelboim, O.; Beauchemin, N.; et al. CEACAM1 on activated NK cells inhibits NKG2D-mediated cytolytic function and signaling. *Eur. J. Immunol.* **2013**, *43*, 2473–2483, doi:10.1002/EJI.201242676.
58. Pils, S.; Gerrard, D.T.; Meyer, A.; Hauck, C.R. CEACAM3: An innate immune receptor directed against human-restricted bacterial pathogens. *Int. J. Med. Microbiol.* **2008**, *298*, 553–560, doi:10.1016/J.IJMM.2008.04.005.
59. Kono, Y. Apparent antibacterial activity of catalase: role of lipid hydroperoxide contamination. *J. Biochem.* **1995**, *117*, 42–46, doi:10.1093/OXFORDJOURNALS.JBCHEM.A124718.
60. Patel, S.; Homaei, A.; El-Seedi, H.R.; Akhtar, N. Cathepsins: Proteases that are vital for survival but can also be fatal. *Biomed. Pharmacother.* **2018**, *105*, 526, doi:10.1016/J.BIOPHA.2018.05.148.
61. Colomb, F.; Wang, W.; Simpson, D.; Zafar, M.; Beynon, R.; Rhodes, J.M.; Yu, L.G. Galectin-3 interacts with the cell-surface glycoprotein CD146 (MCAM, MUC18) and induces secretion of metastasis-promoting cytokines from vascular endothelial cells. *J. Biol. Chem.* **2017**, *292*, 8381–8389, doi:10.1074/JBC.M117.783431.
62. Linder, M.C. Ceruloplasmin and other copper binding components of blood plasma and their functions: an update. *Metallomics* **2016**, *8*, 887–905, doi:10.1039/C6MT00103C.
63. Dela Cruz, C.S.; Liu, W.; He, C.H.; Jacoby, A.; Gornitzky, A.; Ma, B.; Flavell, R.; Lee, C.G.; Elias, J.A. Chitinase 3-like-1 promotes Streptococcus pneumoniae killing and augments host tolerance to lung antibacterial responses. *Cell Host Microbe* **2012**, *12*, 34–46, doi:10.1016/J.CHOM.2012.05.017.
64. Hall, A.J.; Quinnell, R.J.; Raiko, A.; Lagog, M.; Siba, P.; Morroll, S.; Falcone, F.H. Chitotriosidase deficiency is not associated with human hookworm infection in a Papua New Guinean population. *Infect. Genet. Evol.* **2007**, *7*, 743–747, doi:10.1016/J.MEEGID.2007.07.010.
65. Briolat, J.; Wu, S.D.; Mahata, S.K.; Gonthier, B.; Bagnard, D.; Chasserot-Golaz, S.; Helle, K.B.; Aunis, D.; Metz-Boutigue, M.H. New antimicrobial activity for the catecholamine release-inhibitory peptide from chromogranin A. *Cell. Mol. Life Sci.* **2005**, *62*, 377–385, doi:10.1007/S00018-004-4461-9.
66. Jeong, S.; Ledee, D.R.; Gordon, G.M.; Itakura, T.; Patel, N.; Martin, A.; Fini, M.E. Interaction of clusterin and matrix metalloproteinase-9 and its implication for epithelial homeostasis and inflammation. *Am. J. Pathol.* **2012**, *180*, 2028–39, doi:10.1016/j.ajpath.2012.01.025.
67. Verdijk, P.; van Veelen, P.A.; de Ru, A.H.; Hensbergen, P.J.; Mizuno, K.; Koerten, H.K.; Koning, F.; Tensen, C.P.; Mommaas, A.M. Morphological changes during dendritic cell maturation correlate with cofilin activation and translocation to the cell membrane. *Eur. J. Immunol.* **2004**, *34*, 156–164, doi:10.1002/EJI.200324241.
68. Hoeksema, M.; Van Eijk, M.; Haagsman, H.P.; Hartshorn, K.L. Histones as mediators of host defense, inflammation and thrombosis. *http://dx.doi.org/10.2217/fmb.15.151* **2016**, *11*, 441–453, doi:10.2217/FMB.15.151.
69. Hill, L.A.; Vassiliadi, D.A.; Dimopoulou, I.; Anderson, A.J.; Boyle, L.D.; Kilgour, A.H.M.; Stimson, R.H.; Machado, Y.; Overall, C.M.; Walker, B.R.; et al. Neutrophil elastase-cleaved corticosteroid-binding globulin is absent in human plasma. *J. Endocrinol.* **2019**, *240*, 27–39, doi:10.1530/JOE-18-0479.
70. Sproston, N.R.; Ashworth, J.J. Role of C-reactive protein at sites of inflammation and infection. *Front. Immunol.* **2018**, *9*, 754, doi:10.3389/FIMMU.2018.00754/BIBTEX.
71. Zavasnik-Bergant, T. Cystatin protease inhibitors and immune functions. *Front. Biosci.* **2008**, *13*, 4625–4637, doi:10.2741/3028.
72. Matsushima, M.; Takahashi, T.; Ichinose, M.; Miki, K.; Kurokawa, K.; Takahashi, K. Structural and immunological evidence for the identity of prolyl aminopeptidase with leucyl aminopeptidase. *Biochem. Biophys. Res. Commun.* **1991**, *178*, 1459–1464, doi:10.1016/0006-291X(91)91057-J.
73. Berezniuk, I.; Vu, H.T.; Lyons, P.J.; Sironi, J.J.; Xiao, H.; Burd, B.; Setou, M.; Angeletti, R.H.; Ikegami, K.; Fricker, L.D.

Cytosolic carboxypeptidase 1 is involved in processing α- and β-tubulin. *J. Biol. Chem.* **2012**, *287*, 6503–6517, doi:10.1074/JBC.M111.309138.

74. Tort, O.; Tanco, S.; Rocha, C.; Bièche, I.; Seixas, C.; Bosc, C.; Andrieux, A.; Moutin, M.J.; Avilés, F.X.; Lorenzo, J.; et al. The cytosolic carboxypeptidases CCP2 and CCP3 catalyze posttranslational removal of acidic amino acids. *Mol. Biol. Cell* **2014**, *25*, 3017, doi:10.1091/MBC.E14-06-1072.
75. Bauer, K. Cytosol nonspecific dipeptidase. *Handb. Proteolytic Enzym. Second Ed.* **2004**, *1*, 1020–1022, doi:10.1016/B978-0-12-079611-3.50324-4.
76. Li, J.; Metruccio, M.M.E.; Evans, D.J.; Fleiszig, S.M.J. Mucosal fluid glycoprotein DMBT1 suppresses twitching motility and virulence of the opportunistic pathogen Pseudomonas aeruginosa. *PLoS Pathog.* **2017**, *13*, doi:10.1371/JOURNAL.PPAT.1006392.
77. Rosenstiel, P.; Sina, C.; End, C.; Renner, M.; Lyer, S.; Till, A.; Hellmig, S.; Nikolaus, S.; Fölsch, U.R.; Helmke, B.; et al. Regulation of DMBT1 via NOD2 and TLR4 in intestinal epithelial cells modulates bacterial recognition and invasion. *J. Immunol.* **2007**, *178*, 8203–8211, doi:10.4049/JIMMUNOL.178.12.8203.
78. Jiménez-Alcázar, M.; Rangaswamy, C.; Panda, R.; Bitterling, J.; Simsek, Y.J.; Long, A.T.; Bilyy, R.; Krenn, V.; Renné, C.; Renné, T.; et al. Host DNases prevent vascular occlusion by neutrophil extracellular traps. *Science* **2017**, *358*, 1202–1206, doi:10.1126/SCIENCE.AAM8897.
79. Liao, R.Z.; Himo, F.; Yu, J.G.; Liu, R.Z. Dipeptide hydrolysis by the dinuclear zinc enzyme human renal dipeptidase: mechanistic insights from DFT calculations. *J. Inorg. Biochem.* **2010**, *104*, 37–46, doi:10.1016/J.JINORGBIO.2009.09.025.
80. Habib, G.M.; Shi, Z.Z.; Cuevas, A.A.; Lieberman, M.W. Identification of two additional members of the membrane-bound dipeptidase family. *FASEB J.* **2003**, *17*, 1313–1315, doi:10.1096/FJ.02-0899FJE.
81. Turk, B.; Turk, D.; Dolenc, I.; Turk, V. Dipeptidyl-Peptidase I. *Handb. Proteolytic Enzym.* **2013**, *2*, 1968–1974, doi:10.1016/B978-0-12-382219-2.00447-6.
82. De Meester, I. Dipeptidyl-Peptidase II. *Handb. Proteolytic Enzym.* **2013**, *3*, 3432–3438, doi:10.1016/B978-0-12-382219-2.00759-6.
83. Prajapati, S.C.; Chauhan, S.S. Dipeptidyl peptidase III: a multifaceted oligopeptide N-end cutter. *FEBS J.* **2011**, *278*, 3256–3276, doi:10.1111/J.1742-4658.2011.08275.X.
84. Misumi, Y.; Ikehara, Y. Dipeptidyl-peptidase IV. *Handb. Proteolytic Enzym.* **2013**, *3*, 3374–3379, doi:10.1016/B978-0-12-382219-2.00745-6.
85. Bjelke, J.R.; Christensen, J.; Nielsen, P.F.; Branner, S.; Kanstrup, A.B.; Wagtmann, N.; Rasmussen, H.B. Dipeptidyl peptidases 8 and 9: specificity and molecular characterization compared with dipeptidyl peptidase IV. *Biochem. J.* **2006**, *396*, 391, doi:10.1042/BJ20060079.
86. Edwards, D.R.; Handsley, M.M.; Pennington, C.J. The ADAM metalloproteinases. *Mol. Aspects Med.* **2008**, *29*, 258, doi:10.1016/J.MAM.2008.08.001.
87. Rocha-Perugini, V.; Gordon-Alonso, M.; Sánchez-Madrid, F. Role of drebrin at the immunological synapse. *Adv. Exp. Med. Biol.* **2017**, *1006*, 271, doi:10.1007/978-4-431-56550-5_15.
88. Caruso, J.A.; Akli, S.; Pageon, L.; Hunt, K.K.; Keyomarsi, K. The serine protease inhibitor elafin maintains normal growth control by opposing the mitogenic effects of neutrophil elastase. *Oncogene 2015 3427* **2014**, *34*, 3556–3567, doi:10.1038/onc.2014.284.
89. Haroon, N.; Inman, R.D. Endoplasmic reticulum aminopeptidases: biology and pathogenic potential. *Nat. Rev. Rheumatol. 2010 68* **2010**, *6*, 461–467, doi:10.1038/nrrheum.2010.85.
90. Torrent, M.; de la Torre, B.G.; Nogués, V.M.; Andreu, D.; Boix, E. Bactericidal and membrane disruption activities of the eosinophil cationic protein are largely retained in an N-terminal fragment. *Biochem. J.* **2009**, *421*, 425–434, doi:10.1042/BJ20082330.
91. Borelli, V.; Vita, F.; Shankar, S.; Soranzo, M.R.; Banfi, E.; Scialino, G.; Brochetta, C.; Zabucchi, G. Human eosinophil

peroxidase induces surface alteration, killing, and lysis of Mycobacterium tuberculosis. *Infect. Immun.* **2003**, *71*, 605–613, doi:10.1128/IAI.71.2.605-613.2003.

92. McKown, R.L.; Coleman Frazier, E. V; Zadrozny, K.K.; Deleault, A.M.; Raab, R.W.; Ryan, D.S.; Sia, R.K.; Lee, J.K.; Laurie, G.W. A cleavage-potentiated fragment of tear lacritin is bactericidal. *J. Biol. Chem.* **2014**, *289*, 22172–82, doi:10.1074/jbc.M114.570143.

93. Gong, Y.; Yu, Z.; Gao, Y.; Deng, L.; Wang, M.; Chen, Y.; Li, J.; Cheng, B. FABP4 inhibitors suppress inflammation and oxidative stress in murine and cell models of acute lung injury. *Biochem. Biophys. Res. Commun.* **2018**, *496*, 1115–1121, doi:10.1016/J.BBRC.2018.01.150.

94. Suojalehto, H.; Kinaret, P.; Kilpeläinen, M.; Toskala, E.; Ahonen, N.; Wolff, H.; Alenius, H.; Puustinen, A. Level of Fatty Acid Binding Protein 5 (FABP5) Is Increased in Sputum of Allergic Asthmatics and Links to Airway Remodeling and Inflammation. *PLoS One* **2015**, *10*, e0127003, doi:10.1371/JOURNAL.PONE.0127003.

95. Tollin, M.; Bergman, P.; Svenberg, T.; Jörnvall, H.; Gudmundsson, G.H.; Agerberth, B. Antimicrobial peptides in the first line defence of human colon mucosa. *Peptides* **2003**, *24*, 523–530, doi:10.1016/S0196-9781(03)00114-1.

96. Hogan, M.C.; Griffin, M.D.; Rossetti, S.; Torres, V.E.; Ward, C.J.; Harris, P.C. PKHDL1, a homolog of the autosomal recessive polycystic kidney disease gene, encodes a receptor with inducible T lymphocyte expression. *Hum. Mol. Genet.* **2003**, *12*, 685–698, doi:10.1093/HMG/DDG068.

97. Hu, J.; Yan, J.; Rao, G.; Latha, K.; Overwijk, W.W.; Heimberger, A.B.; Li, S. THE DUALITY OF FGL2 - SECRETED IMMUNE CHECKPOINT REGULATOR VERSUS MEMBRANE-ASSOCIATED PROCOAGULANT: THERAPEUTIC POTENTIAL AND IMPLICATIONS. *Int. Rev. Immunol.* **2016**, *35*, 325, doi:10.3109/08830185.2014.956360.

98. Park, H.; Staehling, K.; Tsang, M.; Appleby, M.W.; Brunkow, M.E.; Margineantu, D.; Hockenbery, D.M.; Habib, T.; Liggitt, H.D.; Carlson, G.; et al. Disruption of Fnip1 reveals a metabolic checkpoint controlling B lymphocyte development. *Immunity* **2012**, *36*, 769–781, doi:10.1016/J.IMMUNI.2012.02.019.

99. Vankadari, N. Structure of Furin Protease Binding to SARS-CoV-2 Spike Glycoprotein and Implications for Potential Targets and Virulence. *J. Phys. Chem. Lett.* **2020**, *11*, 6655–6663, doi:10.1021/ACS.JPCLETT.0C01698/ASSET/IMAGES/LARGE/JZ0C01698_0004.JPEG.

100. Da Silva, A.J.; Li, Z.; De Vera, C.; Canto, E.; Findell, P.; Rudd, C.E. Cloning of a novel T-cell protein FYB that binds FYN and SH2-domain-containing leukocyte protein 76 and modulates interleukin 2 production. *Proc. Natl. Acad. Sci. U. S. A.* **1997**, *94*, 7493–7498, doi:10.1073/PNAS.94.14.7493/ASSET/66521F97-7EE2-46E5-BD69-13D692DFFC9C/ASSETS/GRAPHIC/PQ1471400005.JPEG.

101. Matsuda, A.; Suzuki, Y.; Honda, G.; Muramatsu, S.; Matsuzaki, O.; Nagano, Y.; Doi, T.; Shimotohno, K.; Harada, T.; Nishida, E.; et al. Large-scale identification and characterization of human genes that activate NF-kappaB and MAPK signaling pathways. *Oncogene* **2003**, *22*, 3307–3318, doi:10.1038/SJ.ONC.1206406.

102. Kubach, J.; Lutter, P.; Bopp, T.; Stoll, S.; Becker, C.; Huter, E.; Richter, C.; Weingarten, P.; Warger, T.; Knop, J.; et al. Human CD4+CD25+ regulatory T cells: proteome analysis identifies galectin-10 as a novel marker essential for their anergy and suppressive function. *Blood* **2007**, *110*, 1550–1558, doi:10.1182/BLOOD-2007-01-069229.

103. Henderson, N.C.; Sethi, T. The regulation of inflammation by galectin-3. *Immunol. Rev.* **2009**, *230*, 160–171, doi:10.1111/J.1600-065X.2009.00794.X.

104. Ullrich, A.; Sures, I.; D'Egidio, M.; Jallal, B.; Powell, T.J.; Herbst, R.; Dreps, A.; Azam, M.; Rubinstein, M.; Natoli, C.; et al. The secreted tumor-associated antigen 90K is a potent immune stimulator. *J. Biol. Chem.* **1994**, *269*, 18401–18407.

105. Loimaranta, V.; Hepojoki, J.; Laaksoaho, O.; Pulliainen, A.T. Galectin-3-binding protein: A multitask glycoprotein with innate immunity functions in viral and bacterial infections. *J. Leukoc. Biol.* **2018**, *104*, 777–786, doi:10.1002/JLB.3VMR0118-036R.

106. Sewgobind, N. V.; Albers, S.; Pieters, R.J. Functions and Inhibition of Galectin-7, an Emerging Target in Cellular Pathophysiology. *Biomol. 2021, Vol. 11, Page 1720* **2021**, *11*, 1720, doi:10.3390/BIOM11111720.

107. Dai, S.-Y.; Nakagawa, R.; Itoh, A.; Murakami, H.; Kashio, Y.; Abe, H.; Katoh, S.; Kontani, K.; Kihara, M.; Zhang, S.-L.; et al. Galectin-9 Induces Maturation of Human Monocyte-Derived Dendritic Cells. *J. Immunol.* **2005**, *175*, 2974–2981, doi:10.4049/JIMMUNOL.175.5.2974.
108. Tang, J. Gastricsin. *Handb. Proteolytic Enzym.* **2013**, *1*, 49–54, doi:10.1016/B978-0-12-382219-2.00007-7.
109. Bucki, R.; Janmey, P.A. Interaction of the gelsolin-derived antibacterial PBP 10 peptide with lipid bilayers and cell membranes. *Antimicrob. Agents Chemother.* **2006**, *50*, 2932–2940, doi:10.1128/AAC.00134-06/ASSET/3D501A8A-20C0-47B3-89F7-E2014C50620B/ASSETS/GRAPHIC/ZAC0090659790006.JPEG.
110. Koistinen, H.; Koistinen, R.; Zhang, W.M.; Valmu, L.; Stenman, U.H. Nexin-1 inhibits the activity of human brain trypsin. *Neuroscience* **2009**, *160*, 97–102, doi:10.1016/J.NEUROSCIENCE.2009.02.042.
111. Gurney, M.E.; Apatoff, B.R.; Spear, G.T.; Baumel, M.J.; Antel, J.P.; Bania, M.B.; Reder, A.T. Neuroleukin: a lymphokine product of lectin-stimulated T cells. *Science* **1986**, *234*, 574–581, doi:10.1126/SCIENCE.3020690.
112. Slusher, B.S.; Rojas, C.; Coyle, J.T. Glutamate Carboxypeptidase II. *Handb. Proteolytic Enzym.* **2013**, *2*, 1620–1627, doi:10.1016/B978-0-12-382219-2.00368-9.
113. O-Wang, J.; Cooper, M.D.; Iturrioz, X.; Llorens-Cortes, C. Glutamyl Aminopeptidase. *Handb. Proteolytic Enzym.* **2013**, *1*, 410–414, doi:10.1016/B978-0-12-382219-2.00082-X.
114. Hughes, M.M.; McGettrick, A.F.; O'Neill, L.A.J. Glutathione and Glutathione Transferase Omega 1 as Key Posttranslational Regulators in Macrophages. *Microbiol. Spectr.* **2017**, *5*, doi:10.1128/MICROBIOLSPEC.MCHD-0044-2016.
115. Wu, Y.; Fan, Y.; Xue, B.; Luo, L.; Shen, J.; Zhang, S.; Jiang, Y.; Yin, Z. Human glutathione S-transferase P1-1 interacts with TRAF2 and regulates TRAF2–ASK1 signals. *Oncogene 2006 2542* **2006**, *25*, 5787–5800, doi:10.1038/sj.onc.1209576.
116. Gao, X.; Wang, X.; Pham, T.H.; Feuerbacher, L.A.; Lubos, M.L.; Huang, M.; Olsen, R.; Mushegian, A.; Slawson, C.; Hardwidge, P.R. NleB, a bacterial effector with glycosyltransferase activity, targets GAPDH function to inhibit NF-κB activation. *Cell Host Microbe* **2013**, *13*, 87–99, doi:10.1016/J.CHOM.2012.11.010.
117. Trapani, J.A. Granzymes: a family of lymphocyte granule serine proteases. *Genome Biol.* **2001**, *2*, reviews3014.1, doi:10.1186/GB-2001-2-12-REVIEWS3014.
118. Yang, D.; Chen, Q.; Hoover, D.M.; Staley, P.; Tucker, K.D.; Lubkowski, J.; Oppenheim, J.J. Many chemokines including CCL20/MIP-3α display antimicrobial activity. *J. Leukoc. Biol.* **2003**, *74*, 448–455, doi:10.1189/JLB.0103024.
119. Tripal, P.; Bauer, M.; Naschberger, E.; Mörtinger, T.; Hohenadl, C.; Cornali, E.; Thurau, M.; Stürzl, M. Unique features of different members of the human guanylate-binding protein family. *J. Interferon Cytokine Res.* **2007**, *27*, 44–52, doi:10.1089/JIR.2007.0086.
120. Yu, P.; Li, Y.; Li, Y.; Miao, Z.; Peppelenbosch, M.P.; Pan, Q. Guanylate-binding protein 2 orchestrates innate immune responses against murine norovirus and is antagonized by the viral protein NS7. *J. Biol. Chem.* **2020**, *295*, 8036–8047, doi:10.1074/JBC.RA120.013544.
121. Shenoy, A.R.; Wellington, D.A.; Kumar, P.; Kassa, H.; Booth, C.J.; Cresswell, P.; MacMicking, J.D. GBP5 promotes NLRP3 inflammasome assembly and immunity in mammals. *Science* **2012**, *336*, 481–485, doi:10.1126/SCIENCE.1217141.
122. MacKellar, M.; Vigerust, D.J. Role of Haptoglobin in Health and Disease: A Focus on Diabetes. *Clin. Diabetes* **2016**, *34*, 148, doi:10.2337/DIACLIN.34.3.148.
123. Drain, J.; Bishop, J.R.; Hajduk, S.L. Haptoglobin-related protein mediates trypanosome lytic factor binding to trypanosomes. *J. Biol. Chem.* **2001**, *276*, 30254–30260, doi:10.1074/JBC.M010198200.
124. Parrow, N.L.; Fleming, R.E.; Minnick, M.F. Sequestration and scavenging of iron in infection. *Infect. Immun.* **2013**, *81*, 3503–3514, doi:10.1128/IAI.00602-13.
125. A. Parish, C.; Jiang, H.; Tokiwa, Y.; Berova, N.; Nakanishi, K.; McCabe, D.; Zuckerman, W.; Ming Xia, M.; E. Gabay, J. Broad-spectrum antimicrobial activity of hemoglobin. *Bioorg. Med. Chem.* **2001**, *9*, 377–382, doi:10.1016/S0968-0896(00)00263-7.

126. Yin, X.; Li, X.; Chen, N.; Mu, L.; Wu, H.; Yang, Y.; Han, K.; Huang, Y.; Wang, B.; Jian, J.; et al. Hemopexin as an acute phase protein regulates the inflammatory response against bacterial infection of Nile tilapia (Oreochromis niloticus). *Int. J. Biol. Macromol.* **2021**, *187*, 166–178, doi:10.1016/J.IJBIOMAC.2021.07.109.

127. He, L.; Vicente, C.P.; Westrick, R.J.; Eitzman, D.T.; Tollefsen, D.M. Heparin cofactor II inhibits arterial thrombosis after endothelial injury. *J. Clin. Invest.* **2002**, *109*, 213, doi:10.1172/JCI13432.

128. Nemeth, E.; Tuttle, M.S.; Powelson, J.; Vaughn, M.D.; Donovan, A.; Ward, D.M.V.; Ganz, T.; Kaplan, J. Hepcidin regulates cellular iron efflux by binding to ferroportin and inducing its internalization. *Science* **2004**, *306*, 2090–2093, doi:10.1126/SCIENCE.1104742.

129. Park, C.H.; Valore, E. V.; Waring, A.J.; Ganz, T. Hepcidin, a urinary antimicrobial peptide synthesized in the liver. *J. Biol. Chem.* **2001**, *276*, 7806–7810, doi:10.1074/JBC.M008922200.

130. Wild, C.A.; Bergmann, C.; Fritz, G.; Schuler, P.; Hoffmann, T.K.; Lotfi, R.; Westendorf, A.; Brandau, S.; Lang, S. HMGB1 conveys immunosuppressive characteristics on regulatory and conventional T cells. *Int. Immunol.* **2012**, *24*, 485–494, doi:10.1093/INTIMM/DXS051.

131. Küchler, R.; Schroeder, B.O.; Jaeger, S.U.; Stange, E.F.; Wehkamp, J. Antimicrobial activity of high-mobility-group box 2: A new function to a well-known protein. *Antimicrob. Agents Chemother.* **2013**, *57*, 4782–4793, doi:10.1128/AAC.00805-13/SUPPL_FILE/ZAC010132184SO1.PDF.

132. Wen, B.; Wei, Y. ting; Zhao, K. The role of high mobility group protein B3 (HMGB3) in tumor proliferation and drug resistance. *Mol. Cell. Biochem.* **2021**, *476*, 1729–1739, doi:10.1007/S11010-020-04015-Y.

133. Rydengård, V.; Olsson, A.K.; Mörgelin, M.; Schmidtchen, A. Histidine-rich glycoprotein exerts antibacterial activity. *FEBS J.* **2007**, *274*, 377–389, doi:10.1111/J.1742-4658.2006.05586.X.

134. Hoeksema, M.; Van Eijk, M.; Haagsman, H.P.; Hartshorn, K.L. Histones as mediators of host defense, inflammation and thrombosis. *http://dx.doi.org/10.2217/fmb.15.151* **2016**, *11*, 441–453, doi:10.2217/FMB.15.151.

135. Zhuo, L.; Kimata, K. Structure and function of inter-alpha-trypsin inhibitor heavy chains. *Connect. Tissue Res.* **2008**, *49*, 311–320, doi:10.1080/03008200802325458.

136. Zhou, X.; Liao, J.; Meyerdierks, A.; Feng, L.; Naumovski, L.; Böttger, E.C.; Omary, M.B. Interferon-alpha induces nmi-IFP35 heterodimeric complex formation that is affected by the phosphorylation of IFP35. *J. Biol. Chem.* **2000**, *275*, 21364–21371, doi:10.1074/JBC.M003177200.

137. Zhou, Z.; Wang, N.; Woodson, S.E.; Dong, Q.; Wang, J.; Liang, Y.; Rijnbrand, R.; Wei, L.; Nichols, J.E.; Guo, J.T.; et al. Antiviral activities of ISG20 in positive-strand RNA virus infections. *Virology* **2011**, *409*, 175–188, doi:10.1016/J.VIROL.2010.10.008.

138. Kalinska, M.; Meyer-Hoffert, U.; Kantyka, T.; Potempa, J. Kallikreins - the melting pot of activity and function. *Biochimie* **2016**, *122*, 270, doi:10.1016/J.BIOCHI.2015.09.023.

139. Morizane, S.; Yamasaki, K.; Kabigting, F.D.; Gallo, R.L. Kallikrein expression and cathelicidin processing are independently controlled in keratinocytes by calcium, vitamin D(3), and retinoic acid. *J. Invest. Dermatol.* **2010**, *130*, 1297–1306, doi:10.1038/JID.2009.435.

140. Rapala-Kozik, M.; Karkowska, J.; Jacher, A.; Golda, A.; Barbasz, A.; Guevara-Lora, I.; Kozik, A. Kininogen adsorption to the cell surface of Candida spp. *Int. Immunopharmacol.* **2008**, *8*, 237–241, doi:10.1016/J.INTIMP.2007.07.005.

141. Ben Nasr, A.; Herwald, H.; Muller-Esterl, W.; Bjorck, L. Human kininogens interact with M protein, a bacterial surface protein and virulence determinant. *Biochem. J.* **1995**, *305*, 173–180, doi:10.1042/BJ3050173.

142. Smith, D.; Tikhonova, I.G.; Jewhurst, H.L.; Drysdale, O.C.; Dvořák, J.; Robinson, M.W.; Cwiklinski, K.; Dalton, J.P. Unexpected Activity of a Novel Kunitz-type Inhibitor: INHIBITION OF CYSTEINE PROTEASES BUT NOT SERINE PROTEASES. *J. Biol. Chem.* **2016**, *291*, 19220, doi:10.1074/JBC.M116.724344.

143. Wijkstrom-Frei, C.; El-Chemaly, S.; Ali-Rachedi, R.; Gerson, C.; Cobas, M.A.; Forteza, R.; Salathe, M.; Conner, G.E. Lactoperoxidase and human airway host defense. *Am. J. Respir. Cell Mol. Biol.* **2003**, *29*, 206–212,

doi:10.1165/RCMB.2002-0152OC.

144. Dall, E.; Brandstetter, H. Structure and function of legumain in health and disease. *Biochimie* **2016**, *122*, 126–150, doi:10.1016/J.BIOCHI.2015.09.022.

145. Torriglia, A.; Martin, E.; Jaadane, I. The hidden side of SERPINB1/Leukocyte Elastase Inhibitor. *Semin. Cell Dev. Biol.* **2017**, *62*, 178–186, doi:10.1016/J.SEMCDB.2016.07.010.

146. Flo, T.H.; Smith, K.D.; Sato, S.; Rodriguez, D.J.; Holmes, M.A.; Strong, R.K.; Akira, S.; Aderem, A. Lipocalin 2 mediates an innate immune response to bacterial infection by sequestrating iron. *Nature* **2004**, *432*, 917–21, doi:10.1038/nature03104.

147. Yang, J.; Goetz, D.; Li, J.Y.; Wang, W.; Mori, K.; Setlik, D.; Du, T.; Erdjument-Bromage, H.; Tempst, P.; Strong, R.; et al. An iron delivery pathway mediated by a lipocalin. *Mol. Cell* **2002**, *10*, 1045–56, doi:10.1016/s1097-2765(02)00710-4.

148. Meng, L.; Song, Z.; Liu, A.; Dahmen, U.; Yang, X.; Fang, H. Effects of Lipopolysaccharide-Binding Protein (LBP) Single Nucleotide Polymorphism (SNP) in Infections, Inflammatory Diseases, Metabolic Disorders and Cancers. *Front. Immunol.* **2021**, *12*, 2469, doi:10.3389/FIMMU.2021.681810/BIBTEX.

149. Krause, A.; Sillard, R.; Kleemeier, B.; Klüver, E.; Maronde, E.; Ramon Conejo-García, J.; Forssmann, W.G.; Schulz-Knappe, P.; Nehls, M.C.; Wattler, F.; et al. Isolation and biochemical characterization of LEAP-2, a novel blood peptide expressed in the liver. *Protein Sci.* **2003**, *12*, 143–152, doi:10.1110/PS.0213603.

150. Yang, D.; Chen, Q.; Hoover, D.M.; Staley, P.; Tucker, K.D.; Lubkowski, J.; Oppenheim, J.J. Many chemokines including CCL20/MIP-3α display antimicrobial activity. *J. Leukoc. Biol.* **2003**, *74*, 448–455, doi:10.1189/JLB.0103024.

151. Oddo, M.; Calandra, T.; Bucala, R.; Meylan, P.R.A. Macrophage migration inhibitory factor reduces the growth of virulent Mycobacterium tuberculosis in human macrophages. *Infect. Immun.* **2005**, *73*, 3783–3786, doi:10.1128/IAI.73.6.3783-3786.2005.

152. Steiner, E.; Holzmann, K.; Pirker, C.; Elbling, L.; Micksche, M.; Sutterlüty, H.; Berger, W. The major vault protein is responsive to and interferes with interferon-gamma-mediated STAT1 signals. *J. Cell Sci.* **2006**, *119*, 459–469, doi:10.1242/JCS.02773.

153. Hassan, E.M.; Willmore, W.G.; McKay, B.C.; DeRosa, M.C. In vitro selections of mammaglobin A and mammaglobin B aptamers for the recognition of circulating breast tumor cells. *Sci. Rep.* **2017**, *7*, doi:10.1038/S41598-017-13751-Z.

154. Pejler, G.; Knight, S.D.; Henningsson, F.; Wernersson, S. Novel insights into the biological function of mast cell carboxypeptidase A. *Trends Immunol.* **2009**, *30*, 401–408, doi:10.1016/J.IT.2009.04.008.

155. Hong, J.-S.; Greenlee, K.J.; Pitchumani, R.; Lee, S.-H.; Song, L.; Shan, M.; Chang, S.H.; Park, P.W.; Dong, C.; Werb, Z.; et al. Dual protective mechanisms of matrix metalloproteinases 2 and 9 in immune defense against Streptococcus pneumoniae. *J. Immunol.* **2011**, *186*, 6427–6436, doi:10.4049/JIMMUNOL.1003449.

156. Kennard, M.L.; Richardson, D.R.; Gabathuler, R.; Ponka, P.; Jefferies, W.A. A novel iron uptake mechanism mediated by GPI-anchored human p97. *EMBO J.* **1995**, *14*, 4178, doi:10.1002/J.1460-2075.1995.TB00091.X.

157. Wojtowicz-Praga, S.M.; Dickson, R.B.; Hawkins, M.J. Matrix metalloproteinase inhibitors. *Invest. New Drugs* **1997**, *15*, 61–75, doi:10.1023/A:1005722729132.

158. Sonobe, Y.; Li, H.; Jin, S.; Kishida, S.; Kadomatsu, K.; Takeuchi, H.; Mizuno, T.; Suzumura, A. Midkine Inhibits Inducible Regulatory T Cell Differentiation by Suppressing the Development of Tolerogenic Dendritic Cells. *J. Immunol.* **2012**, *188*, 2602–2611, doi:10.4049/JIMMUNOL.1102346.

159. Serrador, J.M.; Nieto, M.; Alonso-Lebrero, J.L.; del Pozo, M.A.; Calvo, J.; Furthmayr, H.; Schwartz-Albiez, R.; Lozano, F.; González-Amaro, R.; Sánchez-Mateos, P.; et al. CD43 Interacts With Moesin and Ezrin and Regulates Its Redistribution to the Uropods of T Lymphocytes at the Cell-Cell Contacts. *Blood* **1998**, *91*, 4632–4644, doi:10.1182/BLOOD.V91.12.4632.

160. Linden, S.K.; Sutton, P.; Karlsson, N.G.; Korolik, V.; McGuckin, M.A. Mucins in the mucosal barrier to infection. *Mucosal Immunol. 2008 13* **2008**, *1*, 183–197, doi:10.1038/mi.2008.5.

161. Crisford, H.; Sapey, E.; Stockley, R.A. Proteinase 3; a potential target in chronic obstructive pulmonary disease and other chronic inflammatory diseases. *Respir. Res. 2018 191* **2018**, *19*, 1–10, doi:10.1186/S12931-018-0883-Z.

162. Nauseef, W.M. Myeloperoxidase in human neutrophil host defence. *Cell. Microbiol.* **2014**, *16*, 1146–1155, doi:10.1111/CMI.12312.
163. Mak, P.; Wójcik, K.; Silberring, J.; Dubin, A. Antimicrobial peptides derived from heme-containing proteins: hemocidins. *Antonie Van Leeuwenhoek* **2000**, *77*, 197–207, doi:10.1023/A:1002081605784.
164. Liu, C.; Xu, Z.; Gupta, D.; Dziarski, R. Peptidoglycan recognition proteins: a novel family of four human innate immunity pattern recognition molecules. *J. Biol. Chem.* **2001**, *276*, 34686–34694, doi:10.1074/JBC.M105566200.
165. Nalivaeva, N.N.; Turner, A.J. Neprilysin. *Handb. Proteolytic Enzym.* **2013**, *1*, 612–619, doi:10.1016/B978-0-12-382219-2.00127-7.
166. Manicone, A.M.; McGuire, J.K. Matrix metalloproteinases as modulators of inflammation. *Semin. Cell Dev. Biol.* **2008**, *19*, 34–41, doi:10.1016/J.SEMCDB.2007.07.003.
167. Korkmaz, B.; Gauthier, F. Elastase-2/Leukocyte Elastase. *Handb. Proteolytic Enzym.* **2013**, *3*, 2653–2661, doi:10.1016/B978-0-12-382219-2.00587-1.
168. Romacho, T.; Villalobos, L.A.; Cercas, E.; Carraro, R.; Sánchez-Ferrer, C.F.; Peiró, C. Visfatin as a Novel Mediator Released by Inflamed Human Endothelial Cells. *PLoS One* **2013**, *8*, e78283, doi:10.1371/JOURNAL.PONE.0078283.
169. Feng, Y.; Huang, N.; Wu, Q.; Wang, B. HMGN2: a novel antimicrobial effector molecule of human mononuclear leukocytes? *J. Leukoc. Biol.* **2005**, *78*, 1136–1141, doi:10.1189/JLB.0505280.
170. Sorrentino, S. The eight human "canonical" ribonucleases: Molecular diversity, catalytic properties, and special biological actions of the enzyme proteins. *FEBS Lett.* **2010**, *584*, 2194–2200, doi:10.1016/J.FEBSLET.2010.04.018.
171. Wisner, A.; Dufour, E.; Messaoudi, M.; Nejdi, A.; Marcel, A.; Ungeheuer, M.N.; Rougeot, C. Human Opiorphin, a natural antinociceptive modulator of opioid-dependent pathways. *Proc. Natl. Acad. Sci. U. S. A.* **2006**, *103*, 17979–17984, doi:10.1073/PNAS.0605865103.
172. Regn, M.; Laggerbauer, B.; Jentzsch, C.; Ramanujam, D.; Ahles, A.; Sichler, S.; Calzada-Wack, J.; Koenen, R.R.; Braun, A.; Nieswandt, B.; et al. Peptidase inhibitor 16 is a membrane-tethered regulator of chemerin processing in the myocardium. *J. Mol. Cell. Cardiol.* **2016**, *99*, 57–64, doi:10.1016/J.YJMCC.2016.08.010.
173. Kang, D.; Liu, G.; Lundström, A.; Gelius, E.; Steiner, H. A peptidoglycan recognition protein in innate immunity conserved from insects to humans. *Proc. Natl. Acad. Sci. U. S. A.* **1998**, *95*, 10078–10082, doi:10.1073/PNAS.95.17.10078.
174. Ando, K.; Hiroishi, K.; Kaneko, T.; Moriyama, T.; Muto, Y.; Kayagaki, N.; Yagita, H.; Okumura, K.; Imawari, M. Perforin, Fas/Fas ligand, and TNF-alpha pathways as specific and bystander killing mechanisms of hepatitis C virus-specific human CTL. *J. Immunol.* **1997**, *158*.
175. Xu, S.; Zhao, L.; Larsson, A.; Venge, P. The identification of a phospholipase B precursor in human neutrophils. *FEBS J.* **2009**, *276*, 175–186, doi:10.1111/J.1742-4658.2008.06771.X.
176. Franco-Chuaire, M.L.; Ramírez-Clavijo, S.; Chuaire-Noack, L. Pigment epithelium-derived factor: clinical significance in estrogen-dependent tissues and its potential in cancer therapy. *Iran. J. Basic Med. Sci.* **2015**, *18*, 837.
177. Meijers, J.C.M.; Herwald, H. Protein C inhibitor. *Semin. Thromb. Hemost.* **2011**, *37*, 349–354, doi:10.1055/S-0031-1276583.
178. Lu, X.; Kugadas, A.; Smith-Page, K.; Lamb, J.; Lin, T.; Ru, Y.; Morley, S.C.; Fichorova, R.; Mittal, S.K.; Chauhan, S.K.; et al. Neutrophil L-Plastin Controls Ocular Paucibacteriality and Susceptibility to Keratitis. *Front. Immunol.* **2020**, *11*, 547, doi:10.3389/FIMMU.2020.00547/BIBTEX.
179. Cousineau, S.E.; Rheault, M.; Sagan, S.M. Poly(rC)-Binding Protein 1 Limits Hepatitis C Virus Virion Assembly and Secretion. *Viruses 2022, Vol. 14, Page 291* **2022**, *14*, 291, doi:10.3390/V14020291.
180. Zell, R.; Ihle, Y.; Seitz, S.; Gündel, U.; Wutzler, P.; Görlach, M. Poly(rC)-binding protein 2 interacts with the oligo(rC) tract of coxsackievirus B3. *Biochem. Biophys. Res. Commun.* **2008**, *366*, 917–921, doi:10.1016/J.BBRC.2007.12.038.
181. JENSEN, P.E.H.; STIGBRAND, T. Differences in the proteinase inhibition mechanism of human alpha 2-macroglobulin and pregnancy zone protein. *Eur. J. Biochem.* **1992**, *210*, 1071–1077, doi:10.1111/J.1432-1033.1992.TB17513.X.
182. Wong, L.Y.F.; Cheung, B.M.Y.; Li, Y.Y.; Tang, F. Adrenomedullin is both proinflammatory and antiinflammatory: its effects

on gene expression and secretion of cytokines and macrophage migration inhibitory factor in NR8383 macrophage cell line. *Endocrinology* **2005**, *146*, 1321–1327, doi:10.1210/EN.2004-1080.

183. Allaker, R.P.; Grosvenor, P.W.; McAnerney, D.C.; Sheehan, B.E.; Srikanta, B.H.; Pell, K.; Kapas, S. Mechanisms of adrenomedullin antimicrobial action. *Peptides* **2006**, *27*, 661–666, doi:10.1016/J.PEPTIDES.2005.09.003.

184. Van Damme, P.; Van Hoecke, A.; Lambrechts, D.; Vanacker, P.; Bogaert, E.; Van Swieten, J.; Carmeliet, P.; Van Den Bosch, L.; Robberecht, W. Progranulin functions as a neurotrophic factor to regulate neurite outgrowth and enhance neuronal survival. *J. Cell Biol.* **2008**, *181*, 37–41, doi:10.1083/JCB.200712039.

185. Caputo, E.; Camarca, A.; Moharram, R.; Tornatore, P.; Thatcher, B.; Guardiola, J.; Martin, B.M. Structural study of GCDFP-15/gp17 in disease versus physiological conditions using a proteomic approach. *Biochemistry* **2003**, *42*, 6169–78, doi:10.1021/bi034038a.

186. Hassan, M.I.; Waheed, A.; Yadav, S.; Singh, T.P.; Ahmad, F. Prolactin inducible protein in cancer, fertility and immunoregulation: structure, function and its clinical implications. *Cell. Mol. Life Sci.* **2009**, *66*, 447–59, doi:10.1007/s00018-008-8463-x.

187. Russell, M.W.; Bobek, L.A.; Brock, J.H.; Hajishengallis, G.; Tenovuo, J. Innate Humoral Defense Factors. *Mucosal Immunol.* **2005**, 73, doi:10.1016/B978-012491543-5/50009-7.

188. Männistö, P.T.; García-Horsman, J.A. Mechanism of action of Prolyl oligopeptidase (PREP) in degenerative brain diseases: Has peptidase activity only a modulatory role on the interactions of PREP with proteins? *Front. Aging Neurosci.* **2017**, *9*, 27, doi:10.3389/FNAGI.2017.00027/BIBTEX.

189. Cutuli, M.; Cristiani, S.; Lipton, J.M.; Catania, A. Antimicrobial effects of α-MSH peptides. *J. Leukoc. Biol.* **2000**, *67*, 233–239, doi:10.1002/JLB.67.2.233.

190. Kimura, M.; Shindo, M.; Moriizumi, T.; Tagawa, N.; Fujinami, A.; Kato, I.; Uchida, Y. Salusin-β, an antimicrobially active peptide against Gram-positive bacteria. *Chem. Pharm. Bull. (Tokyo).* **2014**, *62*, 586–590, doi:10.1248/CPB.C14-00103.

191. Darmoise, A.; Maschmeyer, P.; Winau, F. The Immunological Functions of Saposins. *Adv. Immunol.* **2010**, *105*, 25, doi:10.1016/S0065-2776(10)05002-9.

192. Tong, Z.; Illek, B.; Bhagwandin, V.J.; Verghese, G.M.; Caughey, G.H. Prostasin, a membrane-anchored serine peptidase, regulates sodium currents in JME/CF15 cells, a cystic fibrosis airway epithelial cell line. *Am. J. Physiol. Lung Cell. Mol. Physiol.* **2004**, *287*, doi:10.1152/AJPLUNG.00160.2004.

193. Lord, M.S.; Melrose, J.; Day, A.J.; Whitelock, J.M. The Inter-α-Trypsin Inhibitor Family: Versatile Molecules in Biology and Pathology. *J. Histochem. Cytochem.* **2020**, *68*, 907–927, doi:10.1369/0022155420940067.

194. Simon, A.; Kullberg, B.J.; Tripet, B.; Boerman, O.C.; Zeeuwen, P.; Van Der Ven-Jongekrijg, J.; Verweij, P.; Schalkwijk, J.; Hodges, R.; Van Der Meer, J.W.M.; et al. Drosomycin-like defensin, a human homologue of Drosophila melanogaster drosomycin with antifungal activity. *Antimicrob. Agents Chemother.* **2008**, *52*, 1407–1412, doi:10.1128/AAC.00155-07.

195. Yu, J.; Lu, Y.; Li, Y.; Xiao, L.; Xing, Y.; Li, Y.; Wu, L. Role of S100A1 in hypoxia-induced inflammatory response in cardiomyocytes via TLR4/ROS/NF-κB pathway. *J. Pharm. Pharmacol.* **2015**, *67*, 1240–1250, doi:10.1111/JPHP.12415.

196. Xia, C.; Braunstein, Z.; Toomey, A.C.; Zhong, J.; Rao, X. S100 proteins as an important regulator of macrophage inflammation. *Front. Immunol.* **2018**, *8*, 1908, doi:10.3389/FIMMU.2017.01908/BIBTEX.

197. Zhang, L.; Zhu, T.; Miao, H.; Liang, B. The Calcium Binding Protein S100A11 and Its Roles in Diseases. *Front. cell Dev. Biol.* **2021**, *9*, doi:10.3389/FCELL.2021.693262.

198. Carreira, C.M.; LaVallee, T.M.; Tarantini, F.; Jackson, A.; Lathrop, J.T.; Hampton, B.; Burgess, W.H.; Maciag, T. S100A13 is involved in the regulation of fibroblast growth factor-1 and p40 synaptotagmin-1 release in vitro. *J. Biol. Chem.* **1998**, *273*, 22224–22231, doi:10.1074/JBC.273.35.22224.

199. Colón, K.; Speicher, D.W.; Smith, P.; Taylor, M.; Metzger, D.S.; Montaner, L.J.; Tomescu, C. S100A14 is increased in activated NK cells and plasma of HIV-Exposed seronegative people who inject drugs and promotes monocyte-NK crosstalk. *J. Acquir. Immune Defic. Syndr.* **2019**, *80*, 234–241, doi:10.1097/QAI.0000000000001911.

200. Donato, R. Intracellular and extracellular roles of S100 proteins. *Microsc. Res. Tech.* **2003**, *60*, 540–551, doi:10.1002/JEMT.10296.

201. Wu, Y. yuan; Li, X. feng; Wu, S.; Niu, X. ni; Yin, S. qin; Huang, C.; Li, J. Role of the S100 protein family in rheumatoid arthritis. *Arthritis Res. Ther.* **2022**, *24*, doi:10.1186/S13075-022-02727-8.

202. Clauss, A.; Persson, M.; Lilja, H.; Lundwall, Å. Three genes expressing Kunitz domains in the epididymis are related to genes of WFDC-type protease inhibitors and semen coagulum proteins in spite of lacking similarity between their protein products. *BMC Biochem.* **2011**, *12*, 1–13, doi:10.1186/1471-2091-12-55/TABLES/4.

203. Constam, D.B.; Tobler, A.R.; Rensing-Ehl, A.; Kemler, I.; Hersh, L.B.; Fontana, A. Puromycin-sensitive Aminopeptidase: SEQUENCE ANALYSIS, EXPRESSION, AND FUNCTIONAL CHARACTERIZATION. *J. Biol. Chem.* **1995**, *270*, 26931–26939, doi:10.1074/JBC.270.45.26931.

204. Takada, N.; Sanda, T.; Okamoto, H.; Yang, J.-P.; Asamitsu, K.; Sarol, L.; Kimura, G.; Uranishi, H.; Tetsuka, T.; Okamoto, T. RelA-Associated Inhibitor Blocks Transcription of Human Immunodeficiency Virus Type 1 by Inhibiting NF-κB and Sp1 Actions. *J. Virol.* **2002**, *76*, 8019–8030, doi:10.1128/JVI.76.16.8019-8030.2002/ASSET/7564AA0A-E691-4631-8477-58145AFD16A2/ASSETS/GRAPHIC/JV1620176006.JPEG.

205. Golda, M.; Mótyán, J.A.; Nagy, K.; Matúz, K.; Nagy, T.; Tőzsér, J. Biochemical Characterization of Human Retroviral-Like Aspartic Protease 1 (ASPRV1). *Biomolecules* **2020**, *10*, 1–26, doi:10.3390/BIOM10071004.

206. Campbell, G.; Swamynathan, S.; Tiwari, A.; Swamynathan, S.K. The secreted Ly-6/uPAR related protein-1 (SLURP1) stabilizes epithelial cell junctions and suppresses TNF-α-induced cytokine production. *Biochem. Biophys. Res. Commun.* **2019**, *517*, 729–734, doi:10.1016/J.BBRC.2019.07.123.

207. Lu, X.; Wang, N.; Long, X.B.; You, X.J.; Cui, Y.H.; Liu, Z. The cytokine-driven regulation of secretoglobins in normal human upper airway and their expression, particularly that of uteroglobin-related protein 1, in chronic rhinosinusitis. *Respir. Res.* **2011**, *12*, 1–10, doi:10.1186/1465-9921-12-28/TABLES/3.

208. Yamada, A.; Suzuki, D.; Miyazono, A.; Oshima, K.; Kamiya, A.; Zhao, B.; Takami, M.; Donnelly, R.P.; Itabe, H.; Yamamoto, M.; et al. IFN-gamma down-regulates Secretoglobin 3A1 gene expression. *Biochem. Biophys. Res. Commun.* **2009**, *379*, 964–968, doi:10.1016/J.BBRC.2008.12.187.

209. De Lamirande, E. Semenogelin, the main protein of the human semen coagulum, regulates sperm function. *Semin. Thromb. Hemost.* **2007**, *33*, 60–68, doi:10.1055/S-2006-958463.

210. Zhao, H.; Lee, W.H.; Shen, J.H.; Li, H.; Zhang, Y. Identification of novel semenogelin I-derived antimicrobial peptide from liquefied human seminal plasma. *Peptides* **2008**, *29*, 505–511, doi:10.1016/J.PEPTIDES.2008.01.009.

211. Bourgeon, F.; Evrard, B.; Brillard-Bourdet, M.; Colleu, D.; Jégou, B.; Pineau, C. Involvement of semenogelin-derived peptides in the antibacterial activity of human seminal plasma. *Biol. Reprod.* **2004**, *70*, 768–774, doi:10.1095/BIOLREPROD.103.022533.

212. Owen, C.A. SERINE PROTEINASES. *Encycl. Respir. Med. Four-Volume Set* **2006**, 1–10, doi:10.1016/B0-12-370879-6/00264-7.

213. Liao, C.; Wang, Q.; An, J.; Zhang, M.; Chen, J.; Li, X.; Xiao, L.; Wang, J.; Long, Q.; Liu, J.; et al. SPINKs in Tumors: Potential Therapeutic Targets. *Front. Oncol.* **2022**, *12*, 225, doi:10.3389/FONC.2022.833741/BIBTEX.

214. Von Bonsdorff, L.; Sahlstedt, L.; Ebeling, F.; Ruutu, T.; Parkkinen, J. Apotransferrin administration prevents growth of Staphylococcus epidermidis in serum of stem cell transplant patients by binding of free iron. *FEMS Immunol. Med. Microbiol.* **2003**, *37*, 45–51, doi:10.1016/S0928-8244(03)00109-3.

215. Law, R.H.P.; Zhang, Q.; McGowan, S.; Buckle, A.M.; Silverman, G.A.; Wong, W.; Rosado, C.J.; Langendorf, C.G.; Pike, R.N.; Bird, P.I.; et al. An overview of the serpin superfamily. *Genome Biol.* **2006**, *7*, 1–11, doi:10.1186/GB-2006-7-5-216/FIGURES/3.

216. Gatt, M.E.; Urieli-Shoval, S.; Preciado-Patt, L.; Fridkin, M.; Calco, S.; Azar, Y.; Matzner, Y. Effect of serum amyloid A on selected in vitro functions of isolated human neutrophils. *J. Lab. Clin. Med.* **1998**, *132*, 414–420, doi:10.1016/S0022-2143(98)90112-3.

217. Job, E.R.; Bottazzi, B.; Gilbertson, B.; Edenborough, K.M.; Brown, L.E.; Mantovani, A.; Brooks, A.G.; Reading, P.C. Serum amyloid P is a sialylated glycoprotein inhibitor of influenza A viruses. *PLoS One* **2013**, *8*, doi:10.1371/JOURNAL.PONE.0059623.

218. Zannettino, A.C.W.; Bühring, H.-J.; Niutta, S.; Watt, S.M.; Benton, M.A.; Simmons, P.J. The Sialomucin CD164 (MGC-24v) Is an Adhesive Glycoprotein Expressed by Human Hematopoietic Progenitors and Bone Marrow Stromal Cells That Serves as a Potent Negative Regulator of Hematopoiesis. *Blood* **1998**, *92*, 2613–2628, doi:10.1182/BLOOD.V92.8.2613.

219. Philley, J. V.; Kannan, A.; Dasgupta, S. MDA-9/Syntenin Control. *J. Cell. Physiol.* **2016**, *231*, 545–550, doi:10.1002/JCP.25136.

220. Fiscella, M.; Perry, J.W.; Teng, B.; Bloom, M.; Zhang, C.; Leung, K.; Pukac, L.; Florence, K.; Concepcion, A.; Liu, B.; et al. TIP, a T-cell factor identified using high-throughput screening increases survival in a graft-versus-host disease model. *Nat. Biotechnol.* **2003**, *21*, 302–307, doi:10.1038/NBT797.

221. Jeong, W.; Chang, T.S.; Boja, E.S.; Fales, H.M.; Rhee, S.G. Roles of TRP14, a thioredoxin-related protein in tumor necrosis factor-alpha signaling pathways. *J. Biol. Chem.* **2004**, *279*, 3151–3159, doi:10.1074/JBC.M307959200.

222. Carion, T.W.; Ebrahim, A.S.; Alluri, S.; Ebrahim, T.; Parker, T.; Burns, J.; Sosne, G.; Berger, E.A. Antimicrobial Effects of Thymosin Beta-4 and Ciprofloxacin Adjunctive Therapy in Pseudomonas aeruginosa Induced Keratitis. *Int. J. Mol. Sci. 2020, Vol. 21, Page 6840* **2020**, *21*, 6840, doi:10.3390/IJMS21186840.

223. Jirasakuldech, B.; Schussler, G.C.; Yap, M.G.; Drew, H.; Josephson, A.; Michl, J. A characteristic serpin cleavage product of thyroxine-binding globulin appears in sepsis sera. *J. Clin. Endocrinol. Metab.* **2000**, *85*, 3996–3999, doi:10.1210/JCEM.85.11.6966.

224. Zhang, G.; Ghosh, S. Negative regulation of toll-like receptor-mediated signaling by Tollip. *J. Biol. Chem.* **2002**, *277*, 7059–7065, doi:10.1074/JBC.M109537200.

225. Nicorescu, I.; Timmers, N.; Stroes, E.; Bernelot, S.; Bahjat, M.; Abduzhamalova, N.; Tereschenko, A.; Masenko, V.; Merkulov, E.; Naumov, V. Transgelin as a promising marker for the study of immune status in atherosclerosis. *Atherosclerosis* **2018**, *275*, e109–e110, doi:10.1016/J.ATHEROSCLEROSIS.2018.06.306.

226. Kim, H.R.; Park, J.S.; Karabulut, H.; Yasmin, F.; Jun, C.D. Transgelin-2: A Double-Edged Sword in Immunity and Cancer Metastasis. *Front. Cell Dev. Biol.* **2021**, *9*, 825, doi:10.3389/FCELL.2021.606149/BIBTEX.

227. Fernandez, C.; Burgos, A.; Morales, D.; Rosales-Rojas, R.; Canelo, J.; Vergara-Jaque, A.; Vieira, G.V.; da Silva, R.A.A.; Sales, K.U.; Conboy, M.J.; et al. TMPRSS11a is a novel age-altered, tissue specific regulator of migration and wound healing. *FASEB J.* **2021**, *35*, doi:10.1096/FJ.202002253RRR.

228. Menou, A.; Duitman, J.; Flajolet, P.; Sallenave, J.M.; Mailleux, A.A.; Crestani, B. Human airway trypsin-like protease, a serine protease involved in respiratory diseases. *Am. J. Physiol. Lung Cell. Mol. Physiol.* **2017**, *312*, L657–L668, doi:10.1152/AJPLUNG.00509.2016.

229. Viloria, C.G.; Peinado, J.R.; Astudillo, A.; García-Suárez, O.; González, M. V.; Suárez, C.; Cal, S. Human DESC1 serine protease confers tumorigenic properties to MDCK cells and it is upregulated in tumours of different origin. *Br. J. Cancer 2007 972* **2007**, *97*, 201–209, doi:10.1038/sj.bjc.6603856.

230. Diao, F.; Li, S.; Tian, Y.; Zhang, M.; Xu, L.G.; Zhang, Y.; Wang, R.P.; Chen, D.; Zhai, Z.; Zhong, B.; et al. Negative regulation of MDA5- but not RIG-I-mediated innate antiviral signaling by the dihydroxyacetone kinase. *Proc. Natl. Acad. Sci. U. S. A.* **2007**, *104*, 11706–11711, doi:10.1073/PNAS.0700544104/SUPPL_FILE/00544FIG8.PDF.

231. Sohar, I.; Sleat, D.E.; Lobel, P. Tripeptidyl Peptidase I. *Handb. Proteolytic Enzym.* **2013**, *3*, 3350–3356, doi:10.1016/B978-0-12-382219-2.00740-7.

232. Tomkinson, B. Tripeptidyl-peptidase II: Update on an oldie that still counts. *Biochimie* **2019**, *166*, 27–37, doi:10.1016/J.BIOCHI.2019.05.012.

233. Ghosh, D.; Porter, E.; Shen, B.; Lee, S.K.; Wilk, D.; Drazba, J.; Yadav, S.P.; Crabb, J.W.; Ganz, T.; Bevins, C.L. Paneth cell trypsin is the processing enzyme for human defensin-5. *Nat. Immunol.* **2002**, *3*, 583–590, doi:10.1038/NI797.

234. Katona, G.; Berglund, G.I.; Hajdu, J.; Gráf, L.; Szilágyi, L. Crystal structure reveals basis for the inhibitor resistance of

human brain trypsin. *J. Mol. Biol.* **2002**, *315*, 1209–1218, doi:10.1006/JMBI.2001.5305.

235. Simmons, M.A. Tryptase. *xPharm Compr. Pharmacol. Ref.* **2007**, 1–3, doi:10.1016/B978-008055232-3.62820-X.

236. Devuyst, O.; Olinger, E.; Rampoldi, L. Uromodulin: from physiology to rare and complex kidney disorders. *Nat. Rev. Nephrol. 2017 139* **2017**, *13*, 525–544, doi:10.1038/nrneph.2017.101.

237. Kukulski, W. A glycoprotein in urine binds bacteria and blocks infections. *Science (80-.).* **2020**, *369*, 917–918, doi:10.1126/SCIENCE.ABD7124/ASSET/9EF19817-5E70-44B0-935D-17AFF4C91727/ASSETS/GRAPHIC/369_917_F1.JPEG.

238. Janicova, A.; Becker, N.; Xu, B.; Wutzler, S.; Vollrath, J.T.; Hildebrand, F.; Ehnert, S.; Marzi, I.; Störmann, P.; Relja, B. Endogenous Uteroglobin as Intrinsic Anti-inflammatory Signal Modulates Monocyte and Macrophage Subsets Distribution Upon Sepsis Induced Lung Injury. *Front. Immunol.* **2019**, *10*, doi:10.3389/FIMMU.2019.02276.

239. Kew, R.R. The Vitamin D binding protein and inflammatory injury: A mediator or sentinel of tissue damage? *Front. Endocrinol. (Lausanne).* **2019**, *10*, 470, doi:10.3389/FENDO.2019.00470/BIBTEX.

240. Lundwall, Å.; Clauss, A. Genes encoding WFDC- and Kunitz-type protease inhibitor domains: are they related? *Biochem. Soc. Trans.* **2011**, *39*, 1398–1402, doi:10.1042/BST0391398.

241. Ito, K.; Nakajima, Y.; Yoshimoto, T. Prolyl Aminopeptidase. *Handb. Proteolytic Enzym.* **2013**, *3*, 3438–3443, doi:10.1016/B978-0-12-382219-2.00760-2.

242. Kodama, H. Xaa-Pro Dipeptidase (Eukaryotes). *Handb. Proteolytic Enzym.* **2013**, *2*, 1507–1514, doi:10.1016/B978-0-12-382219-2.00339-2.

243. Hassan, M.I.; Waheed, A.; Yadav, S.; Singh, T.P.; Ahmad, F. Zinc alpha 2-glycoprotein: a multidisciplinary protein. *Mol. Cancer Res.* **2008**, *6*, 892–906, doi:10.1158/1541-7786.MCR-07-2195.

244. Bergström, J.H.; Birchenough, G.M.H.; Katona, G.; Schroeder, B.O.; Schütte, A.; Ermund, A.; Johansson, M.E.V.; Hansson, G.C. Gram-positive bacteria are held at a distance in the colon mucus by the lectin-like protein ZG16. *Proc. Natl. Acad. Sci. U. S. A.* **2016**, *113*, 13833–13838, doi:10.1073/PNAS.1611400113/SUPPL_FILE/PNAS.1611400113.SM07.AVI.

245. Costa-da-Silva, A.C.; Aure, M.H.; Dodge, J.; Martin, D.; Dhamala, S.; Cho, M.; Rose, J.J.; Bassim, C.W.; Ambatipudi, K.; Hakim, F.T.; et al. Salivary ZG16B expression loss follows exocrine gland dysfunction related to oral chronic graft-versus-host disease. *iScience* **2021**, *25*, doi:10.1016/J.ISCI.2021.103592.

246. Oppenheim, F.G.; Xu, T.; McMillian, F.M.; Levitz, S.M.; Diamond, R.D.; Offner, G.D.; Troxler, R.F. Histatins, a novel family of histidine-rich proteins in human parotid secretion. Isolation, characterization, primary structure, and fungistatic effects on Candida albicans. *J. Biol. Chem.* **1988**, *263*, 7472–7477, doi:10.1016/S0021-9258(18)68522-9.

247. Chernyavsky, A.I.; Galitovskiy, V.; Shchepotin, I.B.; Grando, S.A. Anti-inflammatory effects of the nicotinergic peptides SLURP-1 and SLURP-2 on human intestinal epithelial cells and immunocytes. *Biomed Res. Int.* **2014**, *2014*, doi:10.1155/2014/609086.

248. Updegraff, B.L.; Zhou, X.; Guo, Y.; Padanad, M.S.; Chen, P.H.; Yang, C.; Sudderth, J.; Rodriguez-Tirado, C.; Girard, L.; Minna, J.D.; et al. Transmembrane Protease TMPRSS11B Promotes Lung Cancer Growth by Enhancing Lactate Export and Glycolytic Metabolism. *Cell Rep.* **2018**, *25*, 2223, doi:10.1016/J.CELREP.2018.10.100.

249. Orysiak, J.; Lenczowska, M.J.; Multanowski, B.M. Expression of SCGB1C1 gene as a potential marker of susceptibility to upper respiratory tract infections in elite athletes - a pilot study. *Biol. Sport* **2016**, *33*, 107–110, doi:10.5604/20831862.1196510.

250. Atzei, P.; Gargan, S.; Curran, N.; Moynagh, P.N. Cactin targets the MHC class III protein IkappaB-like (IkappaBL) and inhibits NF-kappaB and interferon-regulatory factor signaling pathways. *J. Biol. Chem.* **2010**, *285*, 36804–36817, doi:10.1074/JBC.M110.139113.

251. Fricker, L.D. Carboxypeptidase Z. *Handb. Proteolytic Enzym. Second Ed.* **2004**, *1*, 844–845, doi:10.1016/B978-0-12-079611-3.50257-3.

252. Tasco´n, J.D.; Adrian, J.; Kopp, K.; Scholz, P.; Tschan, M.P.; Kuespert, K.; Hauck, C.R. The granulocyte orphan receptor CEACAM4 is able to trigger phagocytosis of bacteria. *J. Leukoc. Biol.* **2015**, *97*, 521–531, doi:10.1189/JLB.2AB0813-449RR.

253. Sturm, A.; Lensch, M.; André, S.; Kaltner, H.; Wiedenmann, B.; Rosewicz, S.; Dignass, A.U.; Gabius, H.-J. Human Galectin-2:

Novel Inducer of T Cell Apoptosis with Distinct Profile of Caspase Activation. *J. Immunol.* **2004**, *173*, 3825–3837, doi:10.4049/JIMMUNOL.173.6.3825.

254. El Karim, I.A.; Linden, G.J.; Orr, D.F.; Lundy, F.T. Antimicrobial activity of neuropeptides against a range of micro-organisms from skin, oral, respiratory and gastrointestinal tract sites. *J. Neuroimmunol.* **2008**, *200*, 11–16, doi:10.1016/J.JNEUROIM.2008.05.014.

255. Yenugu, S.; Hamil, K.G.; French, F.S.; Hall, S.H. Antimicrobial actions of human and macaque sperm associated antigen (SPAG) 11 isoforms: influence of the N-terminal peptide. *Mol. Cell. Biochem.* **2006**, *284*, 25–37, doi:10.1007/S11010-005-9009-2.
