# Supplementary figures and images for: Chemical Barrier Proteins in Human Body Fluids
Source: Biomedicines. 2022 Jun 22;10(7):1472. doi: 10.3390/biomedicines10071472 (PMC9312486; doi:10.3390/biomedicines10071472)

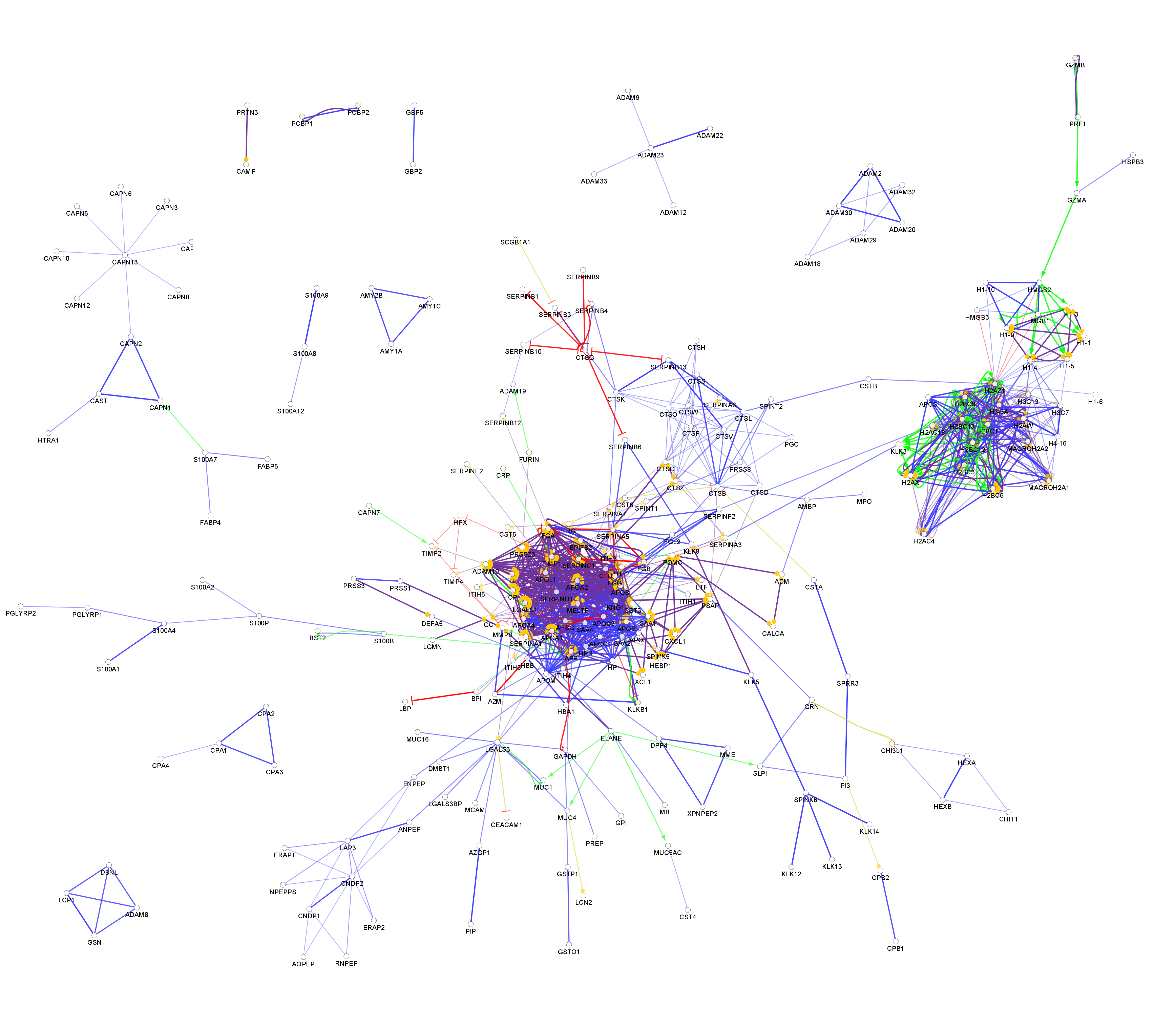

Supplement: Supplementary file 1 [file biomedicines-10-01472-s001.zip › biomedicines-1773728-supplementary/Supplementary/Figure S1.png]

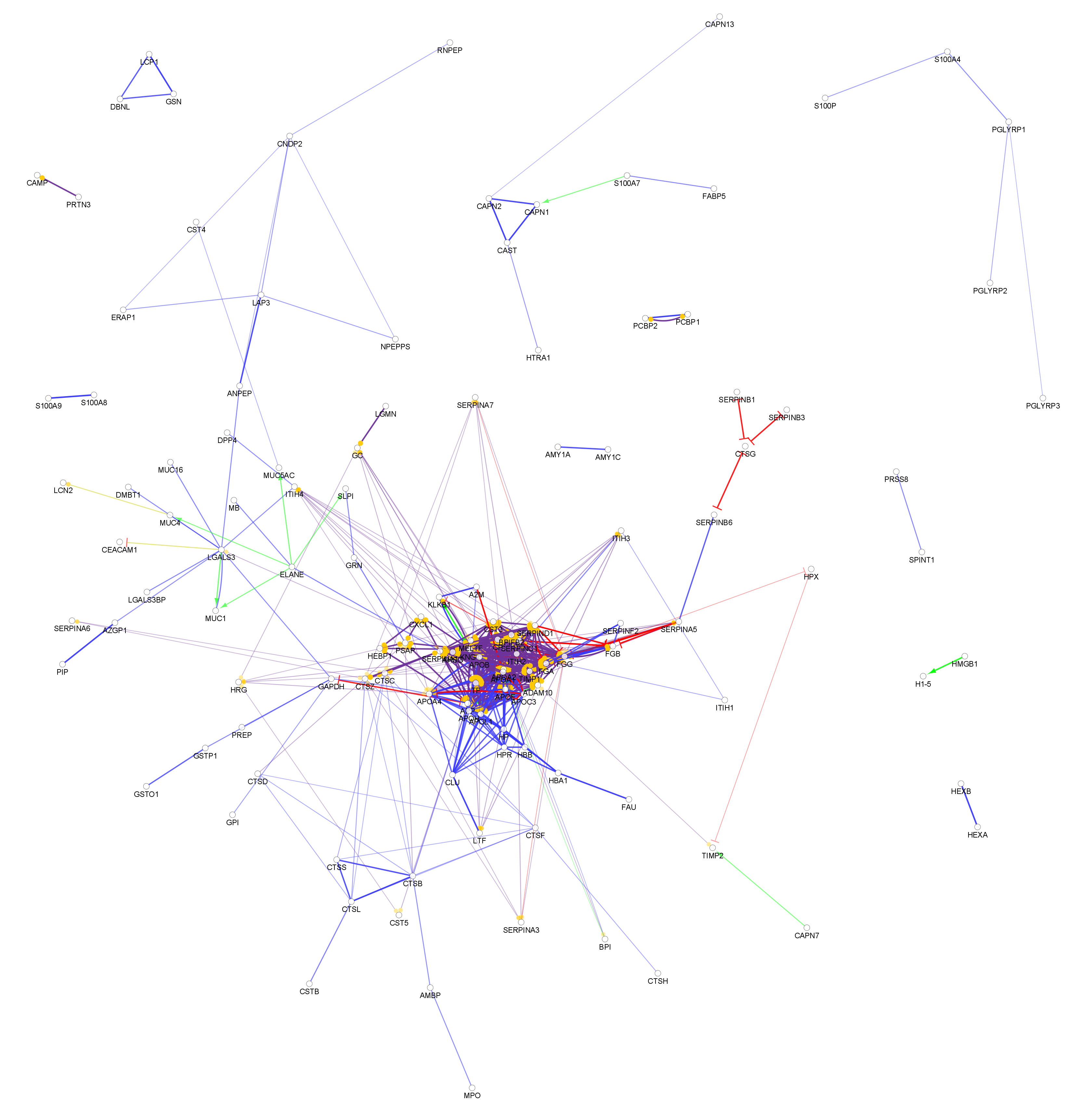

Supplement: Supplementary file 1 [file biomedicines-10-01472-s001.zip › biomedicines-1773728-supplementary/Supplementary/Figure S2.png]

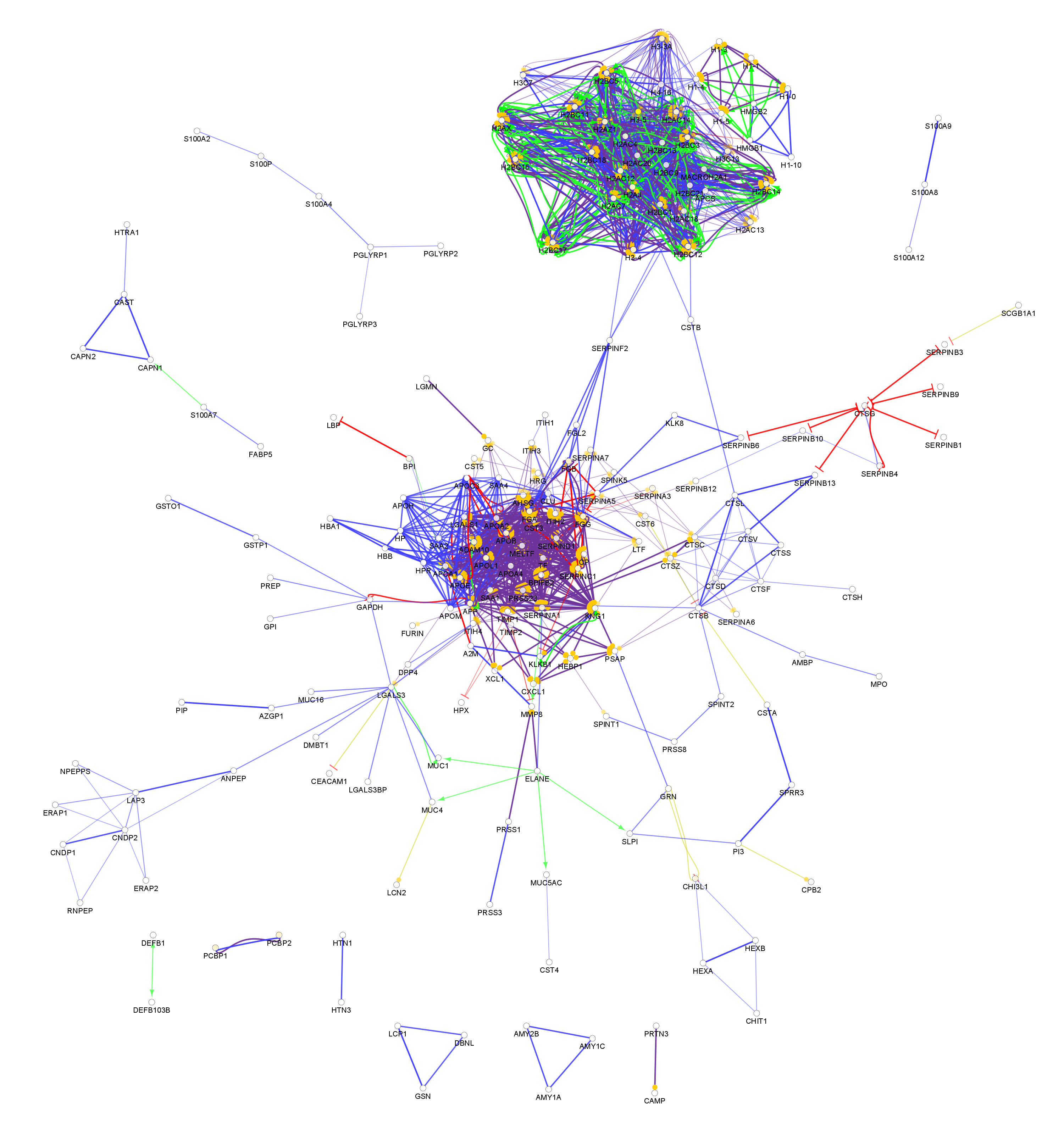

Supplement: Supplementary file 1 [file biomedicines-10-01472-s001.zip › biomedicines-1773728-supplementary/Supplementary/Figure S3.png]

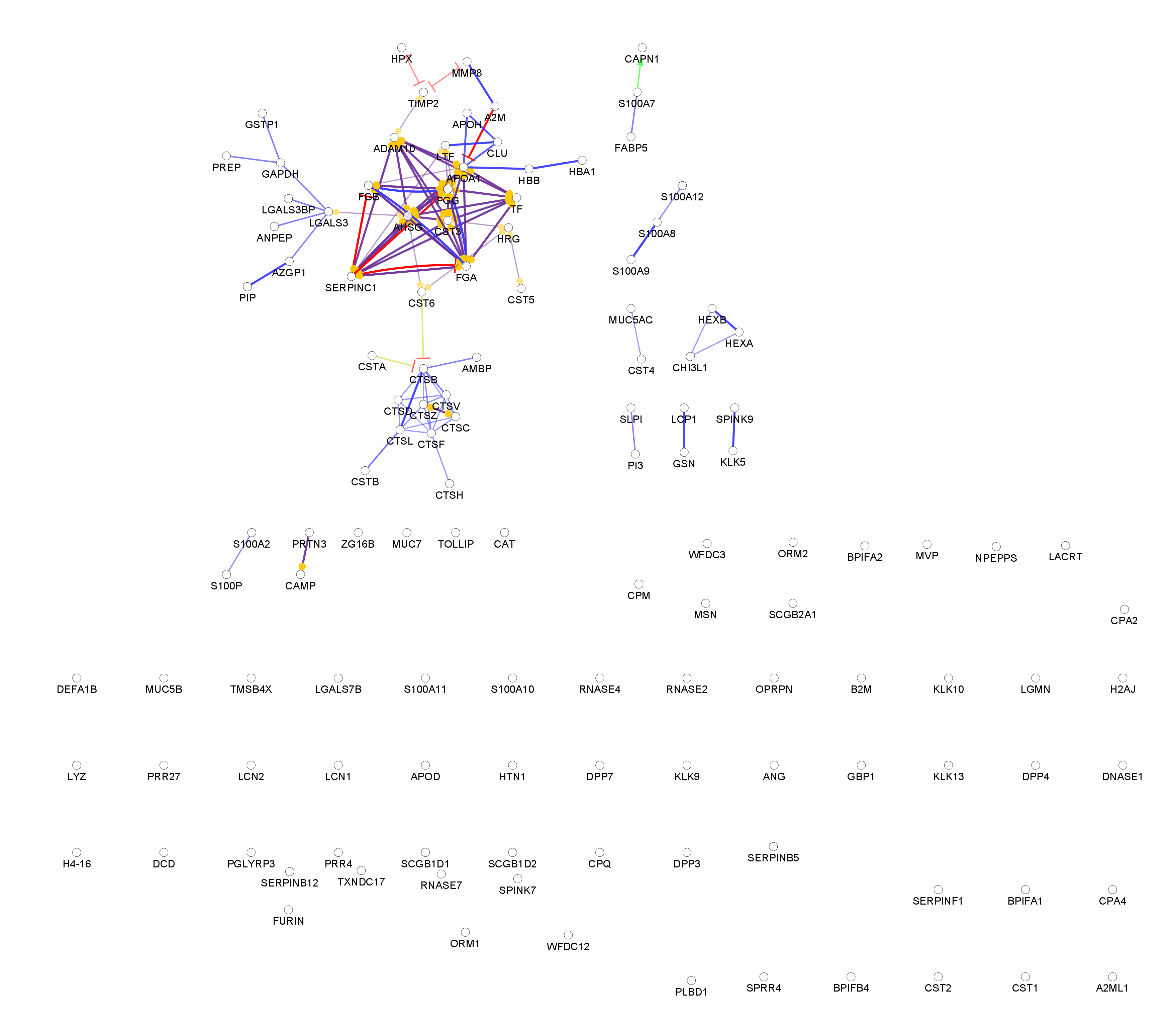

Supplement: Supplementary file 1 [file biomedicines-10-01472-s001.zip › biomedicines-1773728-supplementary/Supplementary/Figure S4.png]

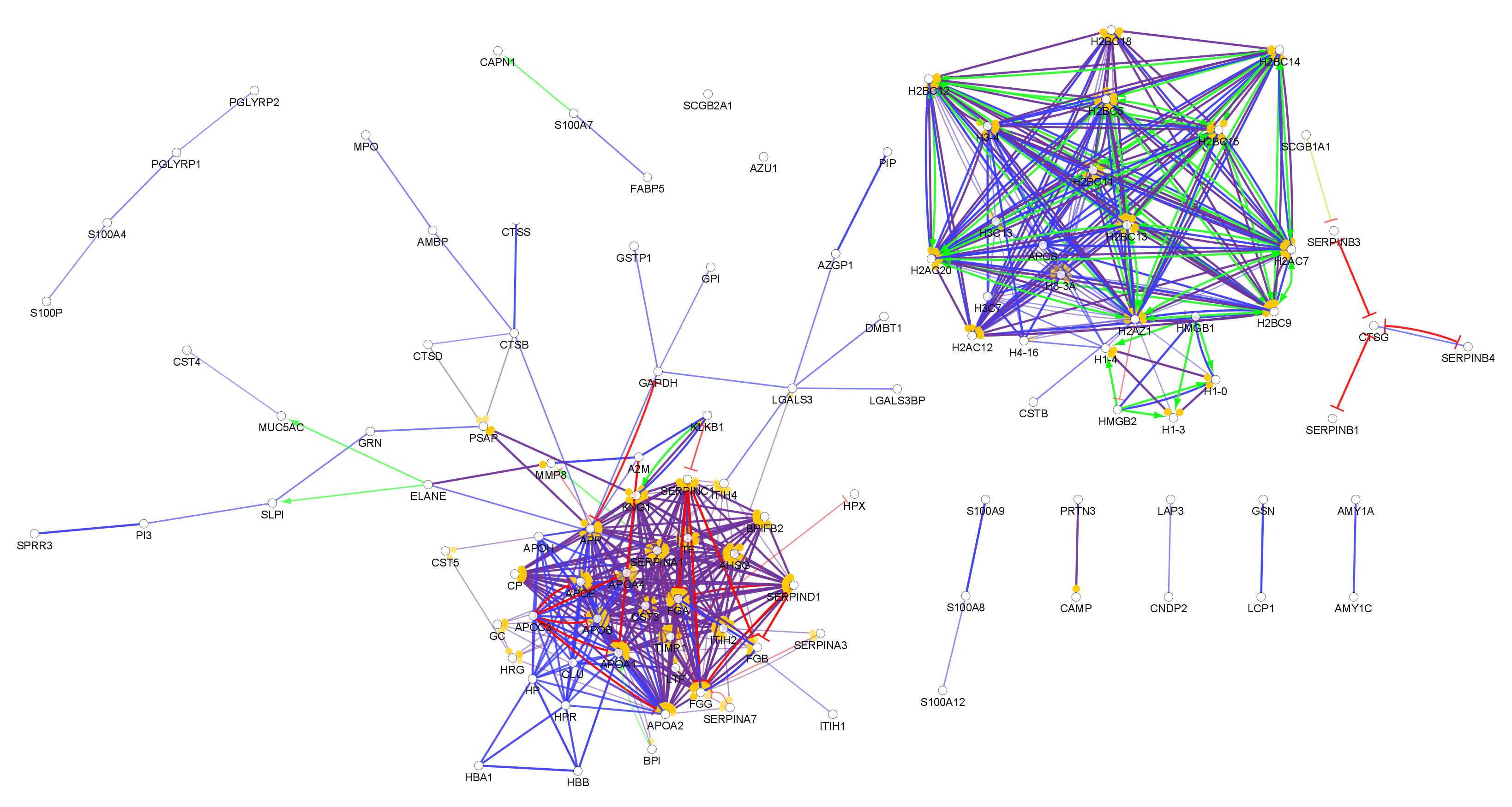

Supplement: Supplementary file 1 [file biomedicines-10-01472-s001.zip › biomedicines-1773728-supplementary/Supplementary/Figure S5.png]

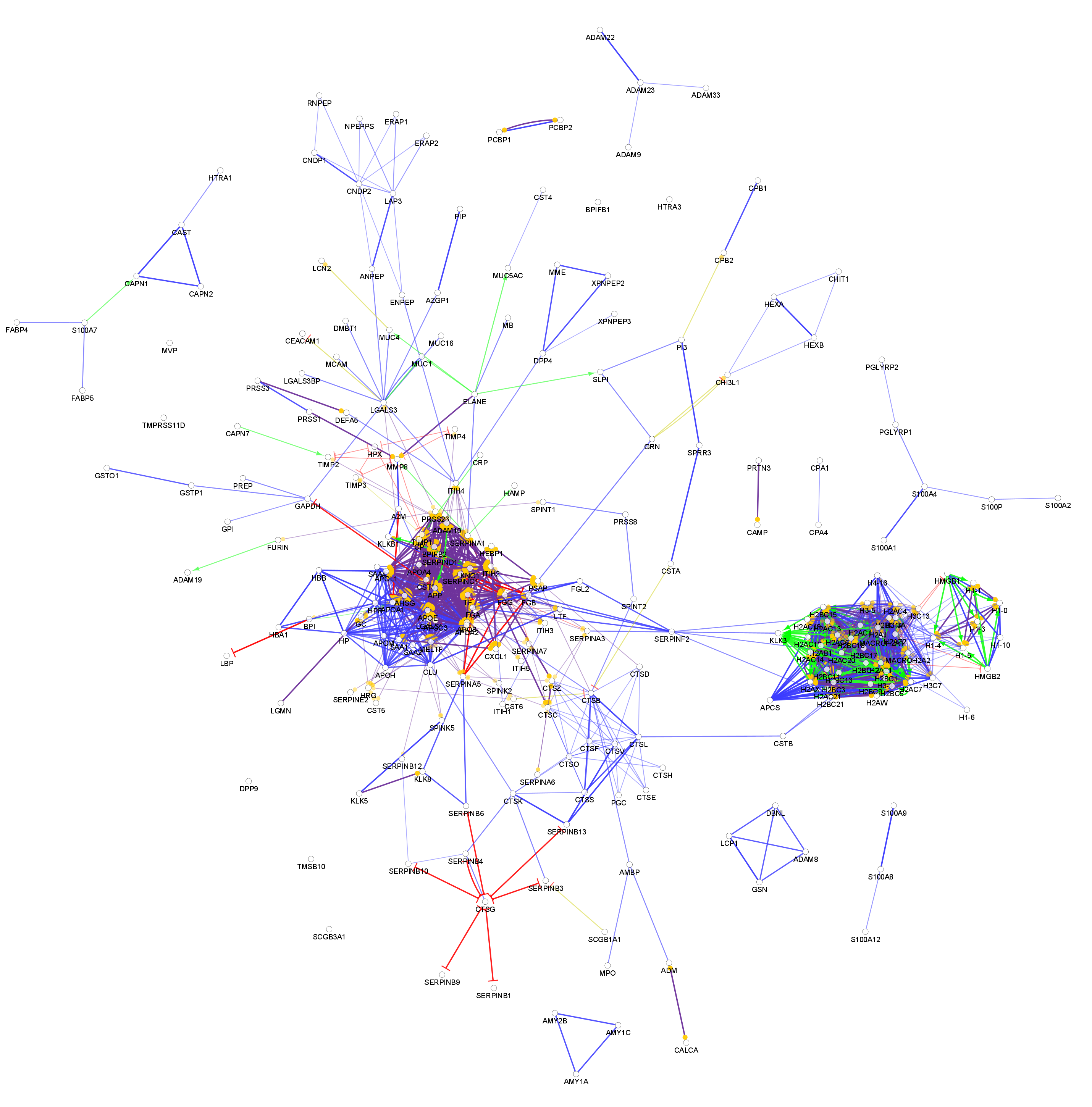

Supplement: Supplementary file 1 [file biomedicines-10-01472-s001.zip › biomedicines-1773728-supplementary/Supplementary/Figure S6.png]

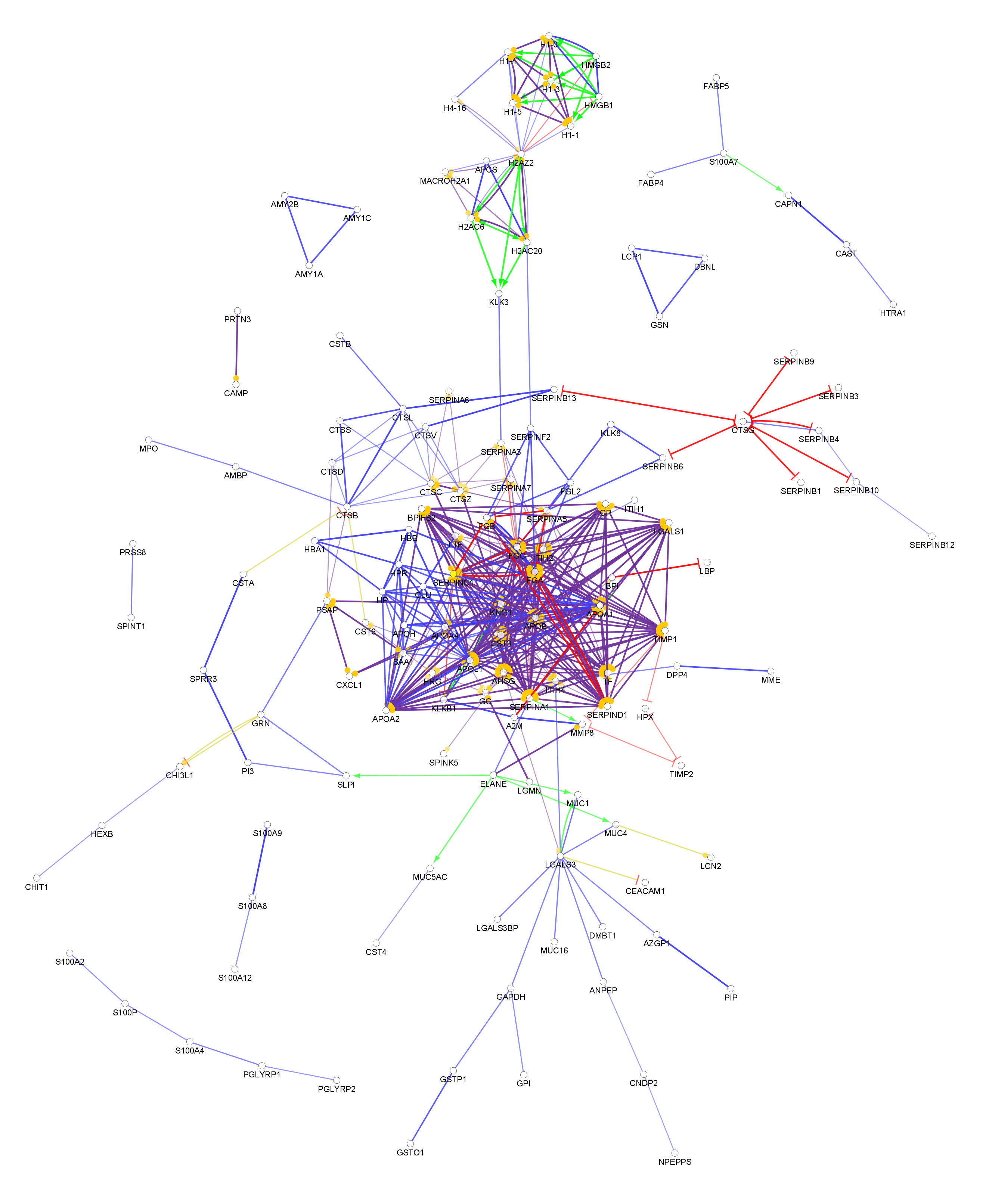

Supplement: Supplementary file 1 [file biomedicines-10-01472-s001.zip › biomedicines-1773728-supplementary/Supplementary/Figure S7.png]

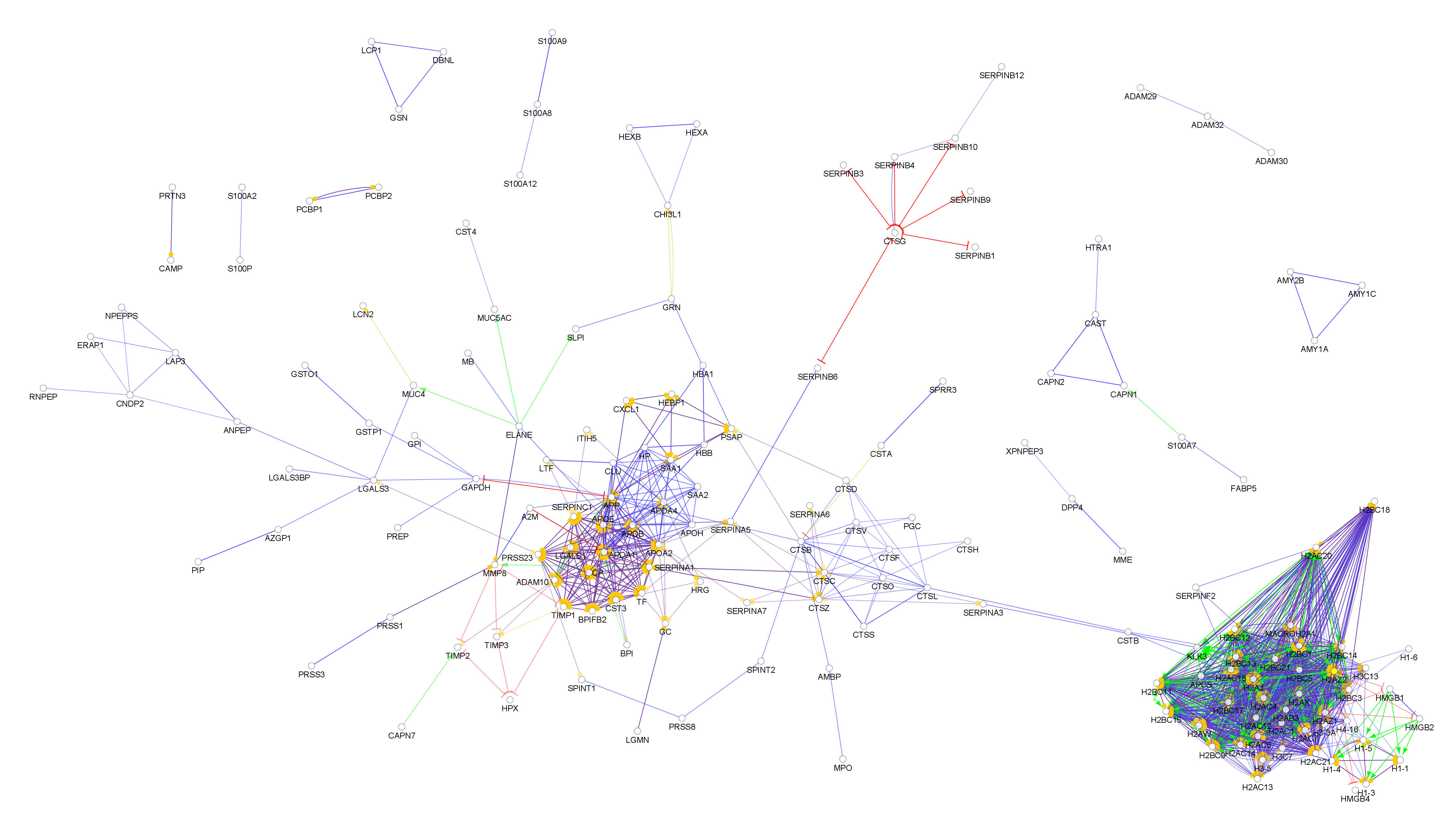

Supplement: Supplementary file 1 [file biomedicines-10-01472-s001.zip › biomedicines-1773728-supplementary/Supplementary/Figure S8.png]

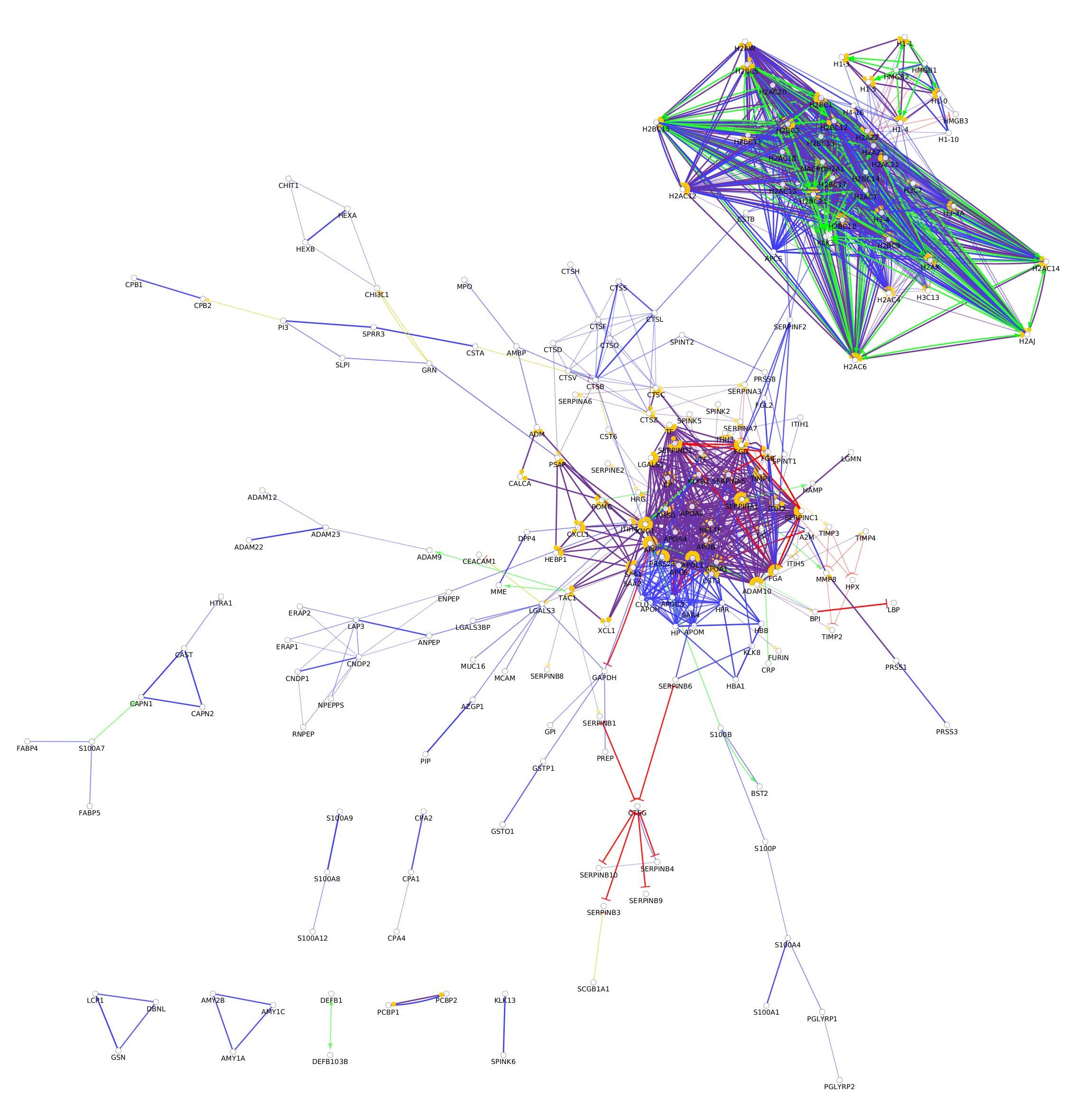

Supplement: Supplementary file 1 [file biomedicines-10-01472-s001.zip › biomedicines-1773728-supplementary/Supplementary/Figure S9.png]
